# An *in vitro* and *in vivo* study on the properties of hollow polycaprolactone cell-delivery particles

**DOI:** 10.1371/journal.pone.0198248

**Published:** 2018-07-03

**Authors:** Barend Andre Stander, Fiona A. van Vollenstee, Karlien Kallmeyer, Marnie Potgieter, Annie Joubert, Andri Swanepoel, Lara Kotze, Sean Moolman, Michael S. Pepper

**Affiliations:** 1 Department of Physiology, Faculty of Medicine, University of Pretoria, Pretoria, South Africa; 2 Institute for Cellular and Molecular Medicine, Department of Immunology, and SAMRC Extramural Unit for Stem Cell Research and Therapy, Faculty of Medicine, University of Pretoria, Pretoria, South Africa; 3 Polymers and Composites Competency Area, Materials Science and Manufacturing, Council for Scientific and Industrial Research, Pretoria, South Africa; Wayne State University, UNITED STATES

## Abstract

The field of dermal fillers is evolving rapidly and numerous products are currently on the market. Biodegradable polymers such as polycaprolactone (PCL) have been found to be compatible with several body tissues, and this makes them an ideal material for dermal filling purposes. Hollow PCL spheres were developed by the Council for Scientific and Industrial Research (CSIR) to serve both as an anchor point and a “tissue harbour” for cells. Particles were tested for cytotoxicity and cell adherence using mouse embryo fibroblasts (MEF). MEFs adhered to the particles and no significant toxic effects were observed based on morphology, cell growth, cell viability and cell cycle analysis, suggesting that the particles are suitable candidates for cell delivery systems in an *in vivo* setting. The objective of providing a “tissue harbour” was however not realized, as cells did not preferentially migrate into the ported particles. *In vivo* studies were conducted in BALB/c mice into whom particles were introduced at the level of the hypodermis. Mice injected with PCL particles (ported and non-ported; with or without MEFs) showed evidence of local inflammation and increased adipogenesis at the site of injection, as well as a systemic inflammatory response. These effects were also observed in mice that received apparently inert (polystyrene) particles. Ported PCL particles can therefore act as a cell delivery system and through their ability to induce adipogenesis, may also serve as a dermal bulking agent.

## Introduction

Dermal filling is a popular method for addressing trauma, disease and age related contour defects of the skin [1, 2]. The size of the USA dermal filler market in 2016 was estimated at 2.6 million doses per annum and increased by 2% from 2015. This market consists of a range of injectable liquids and suspended solids, including hyaluronic acid, calcium hydroxyapatite (Radiesse®) and polymethyl-methacrylate microspheres (Artefill®) [3]. In 2014, the dermal filler portfolio available in Europe was estimated to be exponentially larger than that in the USA [4].

There are at least three different classes of dermal fillers including absorbable products, slowly absorbable products and non-absorbable products [5, 6]. Absorbable products such as hyaluronic acid (HA) [7, 8], collagen fibres, calcium hydroxyapatite, and poly-α-ester [9] fillers last up to 24 months [6]. To maintain the filling effect from absorbable (non-permanent) products, patients need to go for regular filling sessions based on the longevity of the product. This has cost and discomfort implications for the patient; however, the safety of these non-permanent or bio-degradable fillers is arguably higher [1, 10, 11].

An ideal filler should be effective and long lasting, non-immunogenic, non-allergenic, non-carcinogenic, non-teratogenic, cost-effective and provide reproducible results [12]. None of the products on the market meet all these criteria, since dermal fillers can trigger a variety of adverse reactions including inflammation, thrombosis and fibrosis [12]. Polycaprolactone (PCL) is a semicrystalline polymer that is degraded within 2–3 years through slow hydrolysis of ester linkages [13], making it an ideal polymer for long term resorbable dermal fillers. The favourable resorption profile and biocompatibility of PCL has been extensively exploited in implantable medical devices *e*.*g*. a subdermal slow release contraceptive and various drug delivery and tissue engineering applications [14–16]. In 2013, a pilot study using PCL as a dermal filler for hand rejuvenation showed it to be safe, well-tolerated and effective [17].

**Fig 1:**
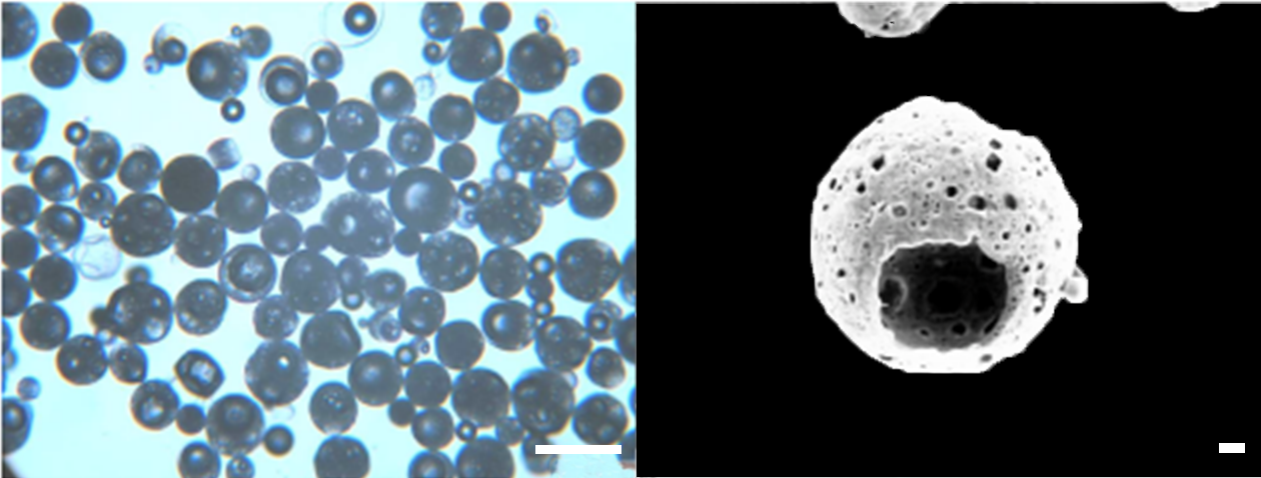
**A) Optical image of ported PCL ported particles; B) SEM image of a ported PCL particle.** A: scale bar = 100 μm and B: scale bar = 10 μm.

Cell-based therapies have great therapeutic promise. Numerous adult cell types have been proposed for the repair of damaged or defective tissues. However, the inability to deliver these cells to specific sites of injury as well as their lack of retention at the site are hurdles that need to be overcome. In order to address these issues the South African Council for Scientific and Industrial Research (CSIR) has developed novel ported PCL particles through a solvent evaporation process that has application in soft tissue bulking and more specifically in minimally invasive dermal filler procedures for wrinkle reduction (Fig 1) [18]. These hollow ported particles were designed to serve both as an anchor point and a “tissue harbour” for cells, which may provide advantages over both the hydrogel (*e*.*g*. HA) and the particulate products currently on the market for soft tissue bulking. “Tissue harbour” refers to the potential advantages conferred by the unique morphology/design of the particle: the hollow particles each have a large port providing a portal of access for cells, so that, subsequent to injection, space is provided for cells within the particles. The microporosity allows for efficient nutrient and oxygen/carbon dioxide exchange within the interior of the particles. The size range of the particles allows for injection of the particles through a 26-gauge needle that is appropriate for facial injection.

The aim of this project was to investigate various aspects of the behaviour of ported PCL particles *in vitro* using mouse embryonic fibroblasts (MEFs) and *in vivo* as cell delivery vehicles. Fibroblasts were used as they are able to generate collagen and thus facilitate dermal bulking and tightening. Morphology, cell growth, cell viability and the effects of the particles on the cell cycle were monitored on MEFs in order to assess whether ported PCL particles are cytocompatible. The *in vivo* effects of (a) the ported PCL particles, (b) MEFs, and (c) ported PCL particles plus MEFs (particles+MEFs), injected into the hypodermis of female BALB/c mice was monitored by observing systemic parameters (clot formation, white blood cell profiles and red blood cell morphology) as well as local histological changes. In order to assess whether the responses observed were due to ported PCL particles, non-ported PCL particles as well as non-resorbable polystyrene (PS) particles were included in a follow up *in vivo* study.

## Materials and methods

### Materials

Dulbecco’s minimum essential medium Eagle (DMEM), penicillin, streptomycin and fungizone were purchased from Highveld Biological (Pty) Ltd. (Sandringham, SA). Sterile cell culture flasks and plates were obtained through Lasec SA (Pty) Ltd. (Honeydew, Johannesburg, South Africa). Gibco® collagenase and hyaluronidase were purchased from Life Technologies (Johannesburg, Gauteng, South Africa). A lactate dehydrogenase cytotoxicity Assay Kit II kit from BioVision Inc. (Mountain View, California, USA) was supplied by BIOCOM biotech (Pty) Ltd. (Clubview, South Africa). All other chemicals of analytical grade, as well as heat-inactivated fetal calf serum (FCS) were purchased from Sigma Chemical Co. (St. Louis, MO, USA).

Ported PCL particles were manufactured according to a previously published protocol (18). Non-ported PCL particles (i.e. PCL particles with no ports) were manufactured as per the published protocol with the omission of the port forming reagents. In a previously unpublished study in rats, we observed that the biotoxicity of PCL particles *in vivo* is low to non-existent, and where present is reflective of a foreign body response (Figs A-E in S1 File and Table A in S2 File, all raw data is in S3 File). PS particles (mean particle diameter of 150 μm) were obtained from Corpuscular Inc. (Cold Spring, New York, USA).

### Animals

BALB/c mice were used for the isolation of MEFs, as well as *in vivo* studies. Approval for the use of female BALB/c mice was obtained from the Animal Use and Care Committee (AUCC), University of Pretoria (approval number H024-09). Animals were kept in conventional Type II mouse cages, females in groups of no more than five and males singly in their cages.

### Isolation of mouse embryonic fibroblasts (MEF)

Females were superovulated at 12h00 on day 0 with 0.2 ml pregnant mare's serum gonadotropin (PMSG) per female administered intraperitoneally (IP). Females were given a second IP injection of 0.2 ml human chorionic gonadotropin (HCG) after 48h at 12h00. They were then placed with a male for mating. Hormones were kept at -20°C until the day of use. Hormones were thawed at room temperature and kept at 4°C. The following morning females were inspected for a copulation plug and left with the male until embryos were flushed.

MEF were chosen as the cell type to be tested with the ported PCL particles. When implanted into syngeneic (BALB/c) mice, these cells would not be rejected. 10 to 12 day BALB/c embryos were first used for isolation purposes, but cell viability was low. Cultures from 15 day embryos were then used, which yielded higher cell numbers with increased viability and proliferation capacity. In addition to embryonic tissue, skin was harvested from the ears of adult BALB/c mice as a source of adult skin fibroblasts (ASF).

Mice were anaesthetised using isofluorane inhalation and terminated by cervical dislocation. Embryos and adult ear skin tissue were immediately isolated and washed several times with DMEM. If limbs or the head were visible, they were removed together with tissue debris. Tissues were then minced finely in DMEM and 5% antibiotics (500 U/ml penicillin G, 500 μg/ml streptomycin and 1250 μg/l fungizone). Resultant tissue fragments were transferred to a tube containing a collagenase (1 mg/ml) and hyaluronidase (1 mg/ml) solution and incubated for three to six hours at 37°C. After incubation, tissue fragments were separated from the digestion solution by low speed centrifugation (300 x *g*) and resuspended in 2.5 ml culture medium containing DMEM, 40% FCS and 1% antibiotics (100 U/ml penicillin G, 100 μg/ml streptomycin and 250 μg/l fungizone). The solution was then transferred into 25 cm^2^ cell culture flasks and incubated for three days in a humidified incubator and 5% CO_2_ at 37°C following which 3 ml culture medium containing DMEM and 1% antibiotics was added. Cultures were left undisturbed for seven days. Thereafter, medium was replaced with DMEM containing 10% FCS and 1% antibiotics every two to three days until 80% confluency was reached, at which point cells were split 1:3 following trypsin (0.25%) digestion, and expanded until enough cells had been obtained for testing purposes.

### *In vitro*: Cell culture

Ported PCL particles were sterilized by washing three times with 70% ethanol and dried under negative pressure. Ported PCL particles (5 mg/ml) were mixed with growth medium and left on a rotary shaker for 1 h at 37°C. A solution of growth medium and ported PCL particles was prepared before exposing MEFs to the particles. Cells were seeded into 96-well tissue culture plates (10 000 cells per well), into 24-well tissue culture plates (500 000 cells in 1 ml per well) or in autoclaved Kimble glass tubes (500 000 cells per 1 ml). Cells were incubated for 24 h to allow for attachment after which medium was removed and cells exposed to 5 mg/ml of ported PCL particles in DMEM containing 10% FCS and 1% antibiotics. Cells were harvested by trypsinization and counted [19]. Non-viable cells were excluded using the trypan blue staining procedure [20]. The number of viable cells per ml was determined as follows: cells/ml = average count of viable cells in the corner squares x dilution factor x 10^4^.

### *In vitro*: Cell growth analysis

Quantification of monolayer cell cultures was determined spectrophotometrically employing crystal violet as a DNA stain [21, 22]. Dye absorbance was measured at 570 nm. Exponentially growing MEF were seeded with ported PCL particles (5 mg/ml) in 96-well tissue culture plates at a cell density of 10 000 cells per well (100 μl/well). After 24 h, 48 h or 72 h medium was discarded and 100 μl of 1% glutaraldehyde (in PBS) was added to each well and incubated at room temperature for 15 min. Glutaraldehyde was discarded and 100 μl 0.1% crystal violet (in PBS) was added and left at room temperature for 30 min. Crystal violet was discarded and the microtitre plates washed under running tap water for 10 min and left overnight to dry. A 200 μl 0.2% Triton X-100 solution was added to solubilise the dye. Samples were then incubated at room temperature for 30 min before 100 μl of the solution was transferred to a clean microtitre plate. Absorbance was determined at 570 nm with an EL_x_800 Universal Microplate Reader from Bio-Tek Instruments Inc. (Vermont, USA).

### *In vitro*: Determination of cell viability

Quantification of plasma membrane damage provides an indication of cell viability. A lactate dehydrogenase (LDH) colorimetric assay [23] was utilized to measure cell viability. The LDH cytotoxicity assay kit utilizes 2-(2-methoxy-4-nitrophenyl)-3-(4-nitrophenyl)-5-(2,4-disulfophenyl)-2H-tetrazolium (WST-8) to react with NADH produced by lactate from released LDH. The intensity of the colour generated correlates directly with the amount of LDH released as a result of plasma membrane damage.

Exponentially growing MEF were seeded with and without ported PCL particles (5 mg/ml) in 24-well tissue culture plates or Kimble glass tubes at a density of 1 x 10^6^ cells per ml (final volume of 1 ml). The cells were incubated at 37°C, 5% CO_2_ for 24 h. Controls were included by adding only growth medium with ported PCL particles (5 mg/ml). A positive control for LDH production was included by adding 10 μl of lysis buffer 30 min before the end of the 24 h incubation period. After the 24 h incubation period, 10 μl of each sample was transferred to a 96-well plate and incubated for 30 min at room temperature with WST-8 reagent as indicated in the supplier’s manual. Absorbance was read at 450 nm with 630 nm as reference using an EL_x_800 Universal Microplate Reader from Bio-Tek Instruments Inc. (Vermont, USA). Cytotoxicity was calculated as follows:

Cytotoxicity(%)=(TS–BC)/(PC–BC)x100.(TS=testsample;BC=backgroundcontrol;PC=positivecontrol)

### *In vitro*: Cell cycle analysis

Flow cytometry was employed to analyse the influence of ported PCL particles (5 mg/ml) on MEF cell cycle progression. Propidium iodide (PI) was used to stain the nucleus in order to determine the stages of the cell cycle during cell division [24]. Exponentially growing MEF were seeded with or without ported PCL particles (5 mg/ml) in Kimble glass tubes at a density of 1 x 10^6^ cells per ml (final volume of 1 ml). Cells were incubated at 37°C, 5% CO_2_ for 24 h. The cells were then trypsinized and resuspended in 1ml growth medium. Cells (1x10^6^/ml) were centrifuged for 5 min at 300 x *g*. The supernatant was discarded and the cells resuspended in 200 μl ice-cold PBS containing 0.1% FCS. Ice-cold 70% ethanol (4 ml) was added in a dropwise manner on a vortex to avoid cell clumping. Cells were stored at 4°C for 24 h and subsequently centrifuged at 300 x *g* for 5 min. The supernatant was removed and the cells resuspended in 1 ml of PBS containing 40 μg/ml PI and 100 μg/ml RNase A. The solution was incubated at 37°C, 5% CO_2_ for 45 min. PI fluorescence (FL3) was measured using a Beckman Coulter South Africa (Pty) Ltd flow cytometer. Data from at least 10 000 cells were analysed with Cyflogic (CyFlo Ltd, Turku, Finland).

### *In vitro*: Morphology

Polarization-optical differential interference contrast (PlasDIC) light microscopy (Carl Zeiss (Pty) Ltd., Johannesburg, South Africa) [25] was employed to monitor the morphology of cells cultured alone or in combination with ported PCL particles. Images were captured using a Zeiss Axiovert-40 microscope (Göttingen, Germany). For fluorescence microscopy, acridine orange was used to stain the cytoplasm of the cells and PI to stain the DNA of cells with compromised cell membranes. Exponentially growing MEF were seeded with, as well as without ported PCL particles (5 mg/ml) in Kimble glass tubes at a density of 1 x 10^6^ cells per ml (final volume of 1 ml). Cells were incubated at 37°C in 5% CO_2_ for 24 h. After 24 h incubation, acridine orange and PI solutions were added to the medium to give a final concentration of 1 μg/ml and 12 μM respectively and incubated for 5 min at 37°C. After 30 min, the medium was removed and the cells carefully rinsed three times with PBS before being immersed in clean PBS (1 ml). Cells were examined with a Zeiss inverted Axiovert CFL40 microscope and photographed using a Zeiss Axiovert MRm monochrome camera using a Zeiss Filter 9 for acridine orange (green) stained cells and a Zeiss Filter 15 for PI (red) stained cells. In order to prevent fluorescent dye quenching, all procedures were performed in a dark room (Carl Zeiss (Pty) Ltd., Johannesburg, South Africa).

Scanning electron microscopy (SEM) was undertaken to determine surface features of the exposed- and control cells. Exponentially growing MEF were seeded with and without ported PCL particles (5 mg/ml) in Kimble glass tubes at a density of 1 x 10^6^ cells per 1 ml (final volume of 1 ml). Cells were incubated at 37°C, 5% CO_2_ for 24 h. After a 24 h incubation, cells were fixed in 2.5% glutaraldehyde in 0.075 M phosphate buffer (pH 7.4–7.6) for 1 h and rinsed three times for 5 min each in 0.075 M phosphate buffer. The cells were then fixed in 0.25% aqueous osmium tetroxide for 30 min and rinsed three times in distilled water in a fume cupboard. Samples were dehydrated in increasing concentrations of ethanol (30%, 50%, 70%, 90% and 3 x 100%), mounted in a chamber and dried using critical point drying. Liquid carbon dioxide (CO_2_) was incorporated into the chamber until it was filled. Ethanol was expelled from the chamber by opening a valve and thereby releasing CO_2_-dissolved ethanol. The valve was closed and the sample was left in liquid CO_2_ for an hour. The vessel was then warmed to 34°C to allow the CO_2_ to reach a gaseous state. Pressure was released slowly; the sample remained in its natural shape and was completely dry. Dried coverslips were mounted on a stub and sprayed with a thin layer of gold. Samples were viewed with a JEOL 840 scanning electron microscope [26].

### *In vitro*: Statistical analysis

Data obtained from three independent experiments (each conducted in six replicates) is shown as the mean ±SD and was statistically analysed for significance using the analysis of variance (ANOVA) single factor model followed by a two-tailed Student’s *t*-test. Means are presented in bar charts with T-bars referring to standard deviations. *P*-values < 0.05 were regarded as statistically significant. Cell number determination, LDH cytotoxic assay and cell cycle progression were analyzed quantitatively, while PlasDIC and transmission electron microscopy were analysed qualitatively.

### *In vivo*: Injection of ported PCL particles and MEFs

BALB/c female mice were used in these experiments. Using a sample micro-riffler, ported PCL particles were sieved in the 100–200 μm range and the sieved particles were aliquoted separately for implantation. Control mice, particle-injected mice, MEF-injected mice and particles+MEF-injected mice were divided into groups of 6, 10, 10 and 10 mice respectively (Table B in S2 File). On day zero, all mice were injected behind the neck at the level of the hypodermis with 0.2 ml of PBS only (control), a ported PCL particle suspension (5 mg/ml in sterile PBS), a MEF cell suspension (1 x 10^6^ cells per 1 ml), or a ported PCL particles+MEF cell suspension. The ported PCL particles+MEF cell suspension was prepared by seeding exponentially growing MEF cells with ported PCL particles (5 mg/ml) in Kimble glass tubes at a density of 1 x 10^6^ cells in 1 ml, and growing them for 24 h in order for the cells to adhere to the particles. The trial period was 59 days, after which all experimental mice were terminated. The injection area was shaved regularly throughout the trial period.

### *In vivo*: Collection of blood samples for fibrin and platelet analysis

On the day of termination, 100 μl to 500 μl of blood was drawn from each mouse and 11 μl citrate was added for every 100 μl of blood. Blood was centrifuged at 1000 rpm for 2 min to obtain platelet rich plasma (PRP). Thrombin (20 U/ml) was used to prepare fibrin clots. Thrombin was prepared in biological buffer containing 0.2% human serum albumin. Thrombin (10 μl) was added to 10 μl PRP. The PRP and thrombin were immediately transferred to a glass cover slip using a pipette tip to form a fibrin clot. The cover slip was placed in a tissue culture dish on filter paper dampened with PBS to create a humid environment and placed at 37°C for 10 min. A washing process followed during which fibrin clots on cover slips were placed in PBS which was stirred using a magnet for 120 min. This was done to remove any blood proteins trapped within the fibrin network.

### *In vivo*: Preparation of washed fibrin clots and whole blood smears for scanning electron microscopy

Washed fibrin clots and whole blood smears were fixed in 2.5% glutaraldehyde in PBS pH 7.4 for 1 h. Sample were rinsed three times for 5 min in PBS and fixed for 1 h in 1% osmium tetroxide (OsO_4_). They were then rinsed three times for 5 min in distilled water and dehydrated serially in increasing concentrations of ethanol (30%, 50%, 70%, 90% and 3 x 100%), dried, mounted and examined with a JEOL 6000F FEGSEM scanning electron microscope (JEOL, USA).

### *In vivo*: Leukocyte counts

Whole blood smears were prepared using 10 μl of whole blood from each animal. A smear was made from drop of blood placed on a glass slide and was dried on a slide warmer. Slides were stained using Giemsa for 5 min, rinsed in water, and dried on a slide warmer. A cover slip was placed on each slide using the mounting agent Entellan. Smears from each of the experimental groups were evaluated by counting 100 white blood cells on each slide; the relative number (percentage) of monocytes, lymphocytes, eosinophils, basophils and neutrophils was determined.

Data was analysed by means of descriptive statistics—means and standard deviations—by group and week for each of the leukocyte types (monocytes, neutrophils, eosinophils, lymphocytes and basophils). Given the small sample size, the analysis was conducted on rank-transformed data. ANOVA on ranks was conducted for each of the leukocyte types to test for a difference between groups and across weeks in terms of rank. A linear model was fit to each of the five leukocyte types. Each outcome (leukocyte type rank) was regressed against group and week to identify significant differences. Significance was set at *P* ≤ 0.05.

### *In vivo*: Preparation of resin-embedded sections for light microscopy

Tissue collected for histological investigation was fixed in 2.5% formaldehyde/glutaraldehyde, removed from the fixative and serially dehydrated in 70% and 90% ethanol, followed by three changes of absolute ethanol. The tissue was orientated, embedded in LR White resin and labelled accordingly. Sections were made on an ultramicrotome. LR White samples were stained with toluidine blue and viewed using a Nikon (Instech Co., Kanagawa, Japan) light microscope.

### *In vivo*: Follow up study with polystyrene particles

BALB/c mice were used in these experiments. Three classes of particles were tested to assess the possible effect of the pore-formation process on inducing systemic inflammation. These included non-ported PCL particles (size range of 100–200 μm), ported PCL particles (mean particle size 150 μm) and polystyrene (PS) particles (mean particle size 150 μm). For particle preparation/suspension prior to injection, particles were aliquoted dry into Eppendorf tubes. Particles were suspended in PBS at least 24 h before injection to allow them to settle and not float on top of the liquid. A pre-determined volume of PBS was added to a pre-prepared number/weight of particles which was dependent on the number of mice to be injected. A concentration of 1.3 mg/50 μl was used (*i*.*e*. 5.2 mg in 200 μl). The sides of the container were carefully rinsed with the PBS in order to ensure that the exact concentration of particles was used. Before injection, the particles were resuspended in the solution by shaking directly before drawing the suspension up through the needle. Each sample was made up in 200 μl to take into account the fact that there would be 100 μl waste in the syringe. 50 μl of PBS with or without particles was injected into the hypodermal region (and not into the dermis) in the nape of the neck by a qualified veterinarian using a 23 gauge needle. Each mouse received a single injection. Before injection, animals were anaesthetized with isoflurane to ensure that they remained still during the procedure. This also ensures that the animals are not uncomfortable.

Animals were euthanized after 0, 1, 2, 4 and 8 weeks and tissue was harvested to determine (1) the extent of inflammation, fibrin morphology and peripheral blood leukocyte counts, and (2) whether the particles could be identified. With regard to the latter, in a pilot experiment, two animals were euthanized 1 h after injection to determine whether particles could be identified. Tissue harvesting involved dissection into the adjacent muscle in order to facilitate orientation during microscopic observation, and full-thickness skin blocks were removed. Histology was also performed on a non-injected site to see whether systemic inflammation induces adipogenesis systemically. An overview of the animals, tests and procedures that were performed are provided in Tables D and E in S2 File.

## Results

### *In vitro*: Cell growth analysis

**Fig 2:**
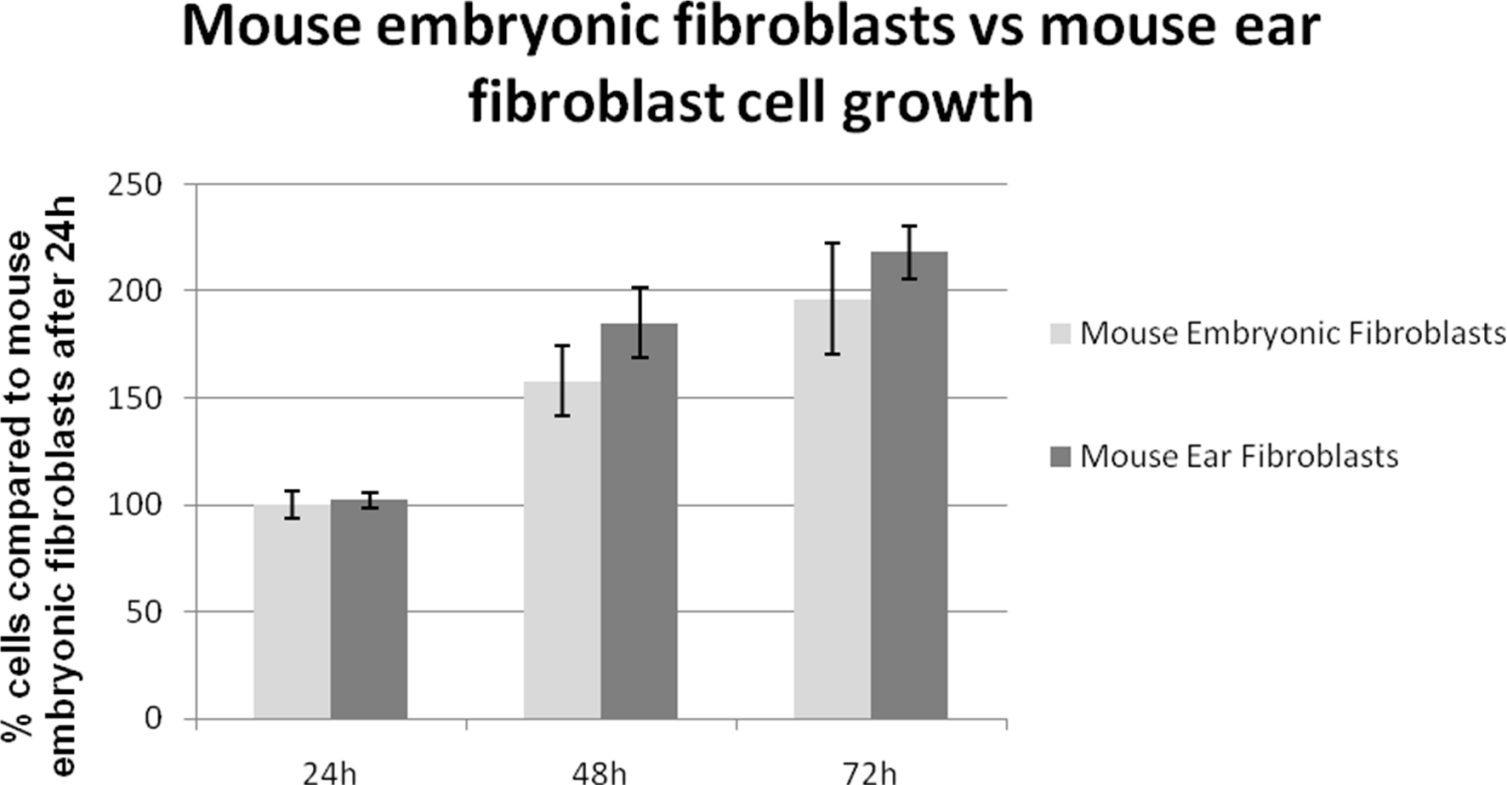
ASF (dark grey) cell numbers are expressed as a percentage of cells relative to the number of MEFs (light grey—100%) counted 24 h after seeding. No significant differences in cell growth were observed after 72 between the two cell types.

Cell growth was determined spectrophotometrically on fixed cell monolayers by employing crystal violet as a DNA stain. The growth rates of ASFs and MEFs were compared over 24 h, 48 h and 72 h (Fig 2). No statistically significant differences were observed indicating that the isolated MEFs had growth rates comparable to ASFs and thus were suitable for further studies.

### *In vitro*: Determination of cell viability

**Fig 3:**
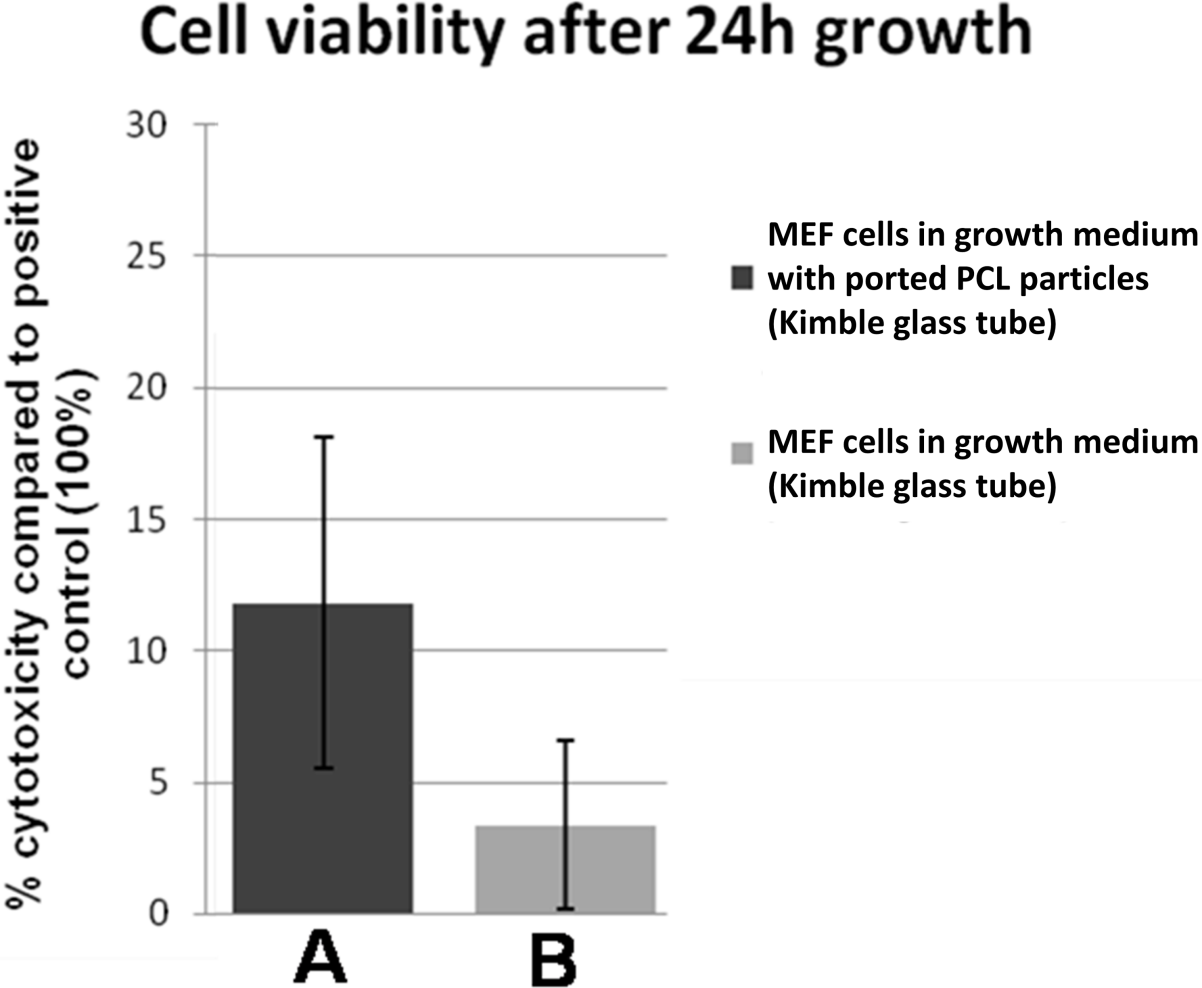
**Viability of MEFs cultured in Kimble glass tubes in growth medium (A) and growth medium together with ported PCL particles (B), 24 h after seeding.** No statistically significant difference was observed (*P*-value > 0.05).

2-(2-Methoxy-4-nitrophenyl)-3-(4-nitrophenyl)-5-(2,4-disulfophenyl)-2H-tetrazolium (WST-8) was used to investigate a possible cytotoxic effect of ported PCL particles on MEFs. WST-8 indirectly measures the amount of extracellular LDH as an indication of cell membrane damage by reacting with NADH produced by lactate from LDH. The intensity of the colour formed by the reaction correlates directly with the amount of LDH released and thus provides an indication of the relative extent of plasma membrane damage caused by a cytotoxic agent [23]. The ability of MEF culture supernatant from control cells or cells attached to ported PCL particles to reduce WST-8 to red formazan (an indication of cytotoxicity) was expressed as a percentage of the positive control (Fig 3). The viability of MEFs cultured in DMEM together with ported PCL particles was not statistically different from cells propagated in DMEM alone (Fig 3 and Table 1), indicating that the ported PCL particles have no major adverse effects on MEF viability.

**Table 1:** Cytotoxity in MEFs cultured in Kimble glass tubes in growth medium or growth medium together with ported PCL particles 24h after seeding.

	% MEF cytototoxicity in growth medium	% MEF cytototoxicity in growth medium with ported PCL particles	*P*-value (ANOVA)
**Kimble glass tube**	3.34	11.81	0.106201*

*No significant difference was observed in cytotoxicity between cells cultured in growth medium and cells cultured in growth medium together with ported PCL particles (*P*-value > 0.05).

### *In vitro*: Cell cycle analysis

**Fig 4:**
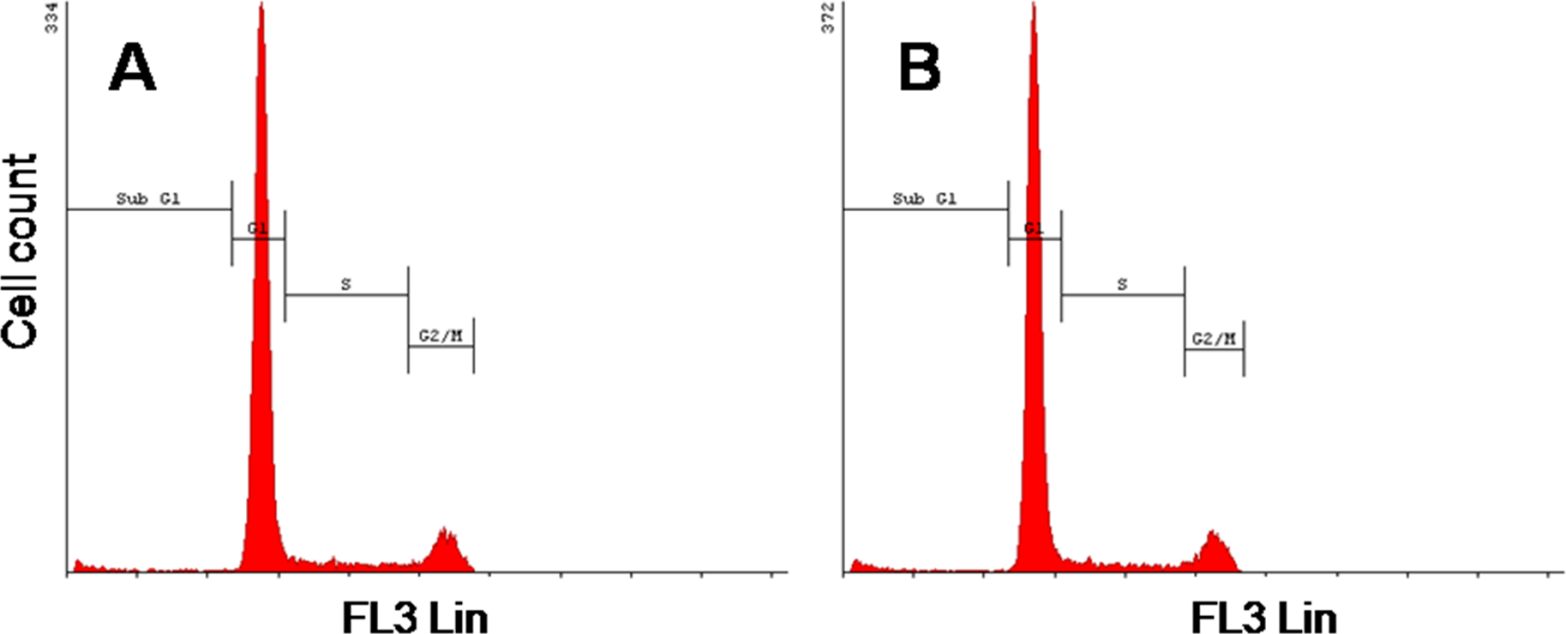
**Cell cycle histograms (FL3 Lin) of MEFs cultured in Kimble glass tubes in growth medium (A) and growth medium together with ported PCL particles (B), 24 h after seeding.** No significant differences were observed.

Cellular DNA content was measured as an indication of cells at the various stages of the cell cycle in order to determine the effect of the ported PCL particles on cell cycle progression. No statistically significant differences in cell division and mitotic activity or the sub G_1_ fraction were observed (Fig 4 and Table 2). The sub G_1_ fraction gives an indication of cell death, including apoptosis. Therefore, the data from the cell cycle analysis further supports the data from the viability assay, indicating that the ported PCL particles do not appear to have an adverse effect on MEFs.

**Table 2:** DNA content of MEFs cultured in growth medium and growth medium together with ported PCL particles at various stages of the cell cycle.

Cell cycle phase	MEF in growth medium	MEF in growth medium with ported PCL particles	*P*-value (ANOVA)
**Sub-G**_**1**_	1.39	2.02	0.168218
**G**_**1**_	79.42	80.77	0.267861
**S**	7.80	6.46	0.091445
**G**_**2**_**/M**	11.39	10.74	0.268161

### *In vitro*: Morphology

**Fig 5:**
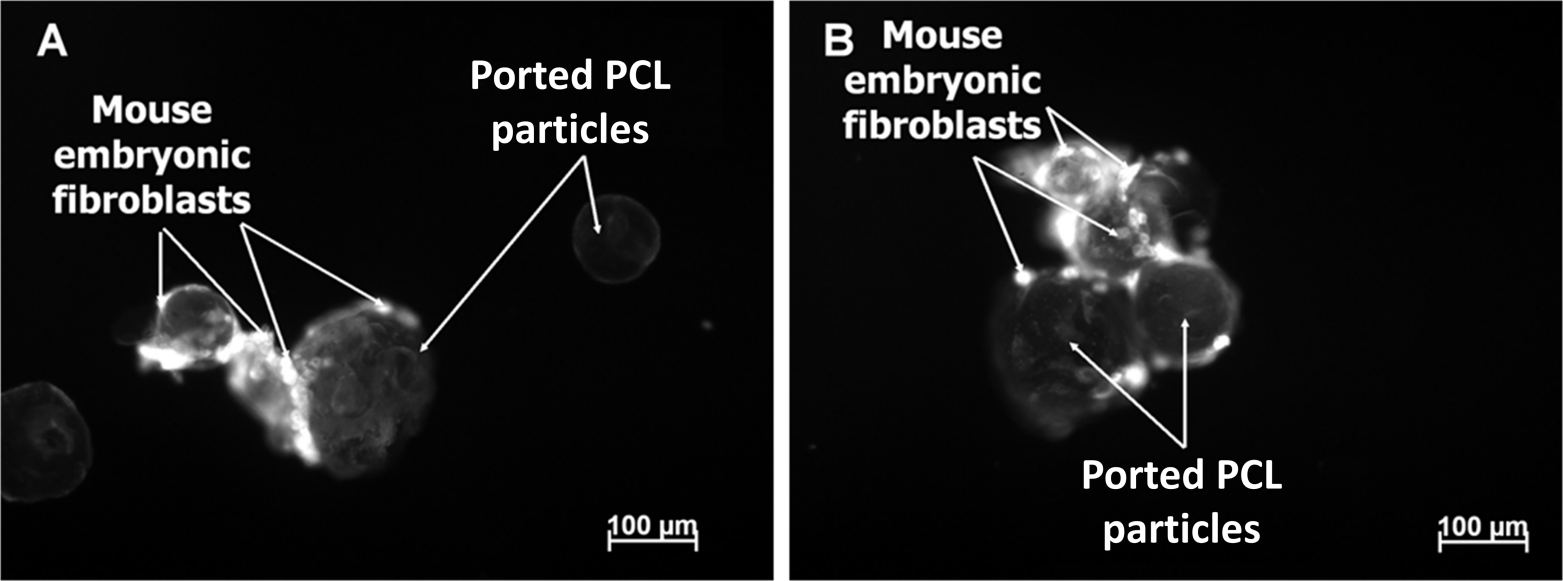
Acridine orange-stained MEFs attached to ported PCL particles. Cells were propagated in glass Kimble tubes together with particles (A and B) and stained 24 h after seeding. MEFs attached to particles as clumps.

Light microscopic analysis of MEFs and AMFs indicates that both cell types exhibit a spindle-shaped appearance after the third passage (third trypsinization step), which is characteristic of normal fibroblasts (data not shown). Acridine orange and PI were used to stain the cytoplasm of intact cells and the DNA of membrane-compromised cells respectively in order to monitor the adherence of the MEFs to the ported PCL particles. When MEFs were cultured in Kimble glass tubes together with ported PCL particles we observed adherence in good numbers of MEFs to the particles (Fig 5). Cells that adhered to the particles did not stain with PI, indicating that the membranes of these cells were intact and that the particles had no apparent adverse effects on MEFs.

**Fig 6:**
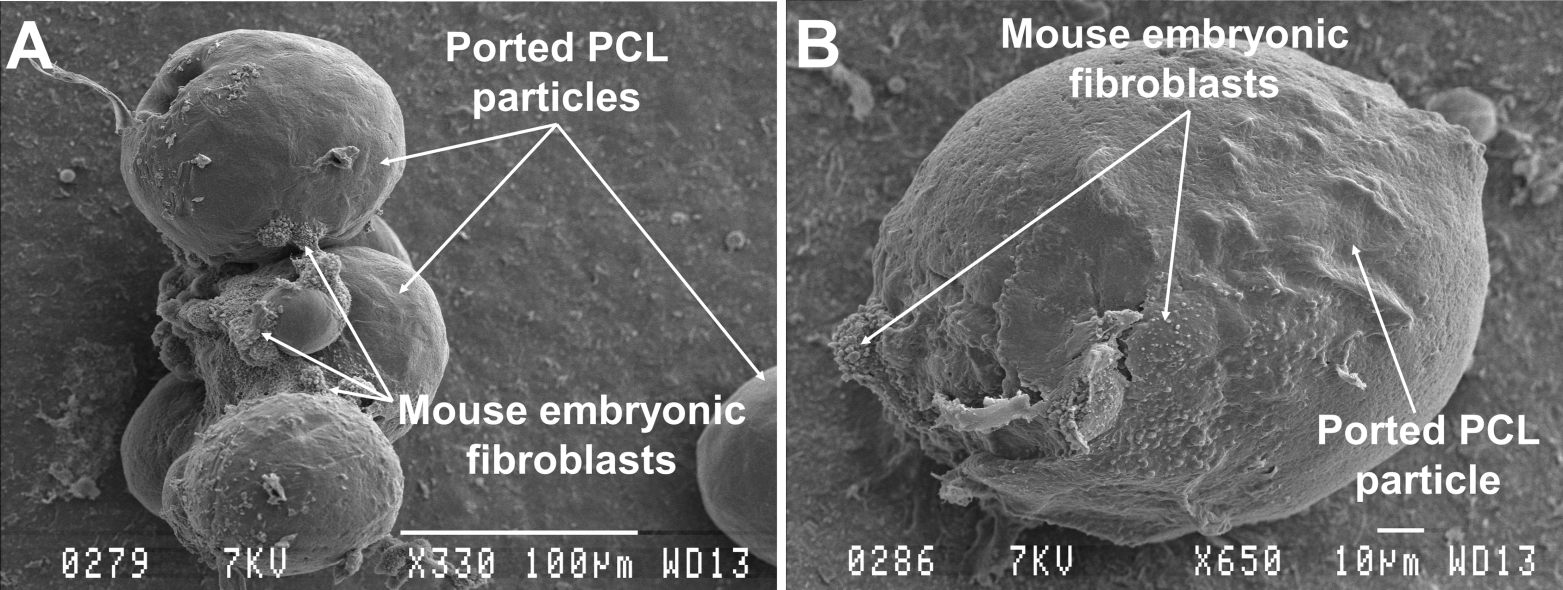
**Scanning electron micrographs of MEFs attached to ported PCL particles 24 h after seeding (A and B).** MEFs grew between particles causing the particles to clump together (A). A: scale bar = 100 μm and B: scale bar = 10 μm.

Scanning electron microscopy was performed to determine the surface features of the ported PCL particles and also of the MEFs that had adhered. MEFs aggregated in small groups between multiple particles (Fig 6), causing particles to clump together. Cells did not appear to migrate into or adhere to the hollow area inside the particles.

### *In vivo* study no. 1: Ported PCL particles +/- MEFs

Mice were observed continuously during the trial period. More than 50% of mice in each experimental group scratched at the site of injection on days 2–4 and wound healing occurred around day 5. On days 25 and 26 the MEF and particles+MEFs groups presented with some swelling at the site of injection (Table B in S2 File).

**Fig 7:**
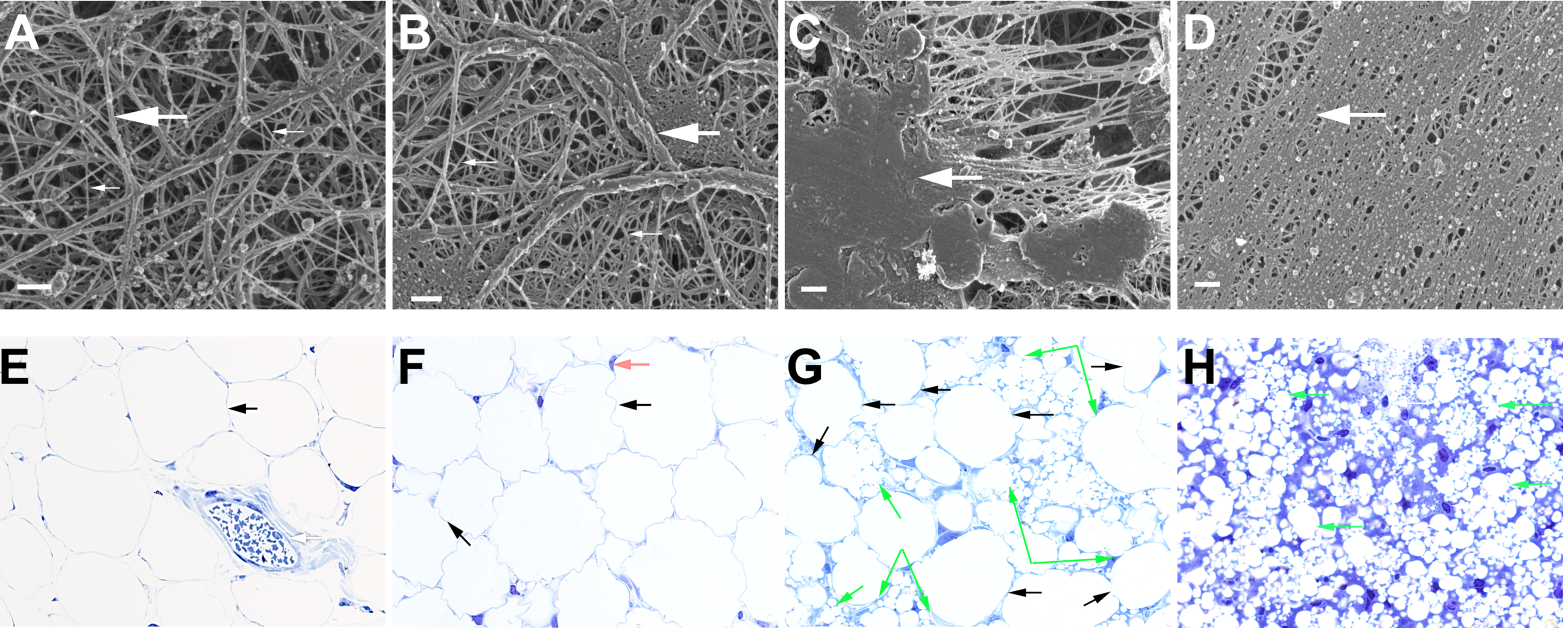
**(A-D) Fibrin network formation as assessed by scanning electron microscopy in BALB/c control (A) MEF-injected (B) ported PCL particle-injected (C) and ported PCL particle+MEFs injected animals (D). (E-H) Light microscopy of control (E), cell injected (F), PCL particle injected (G), and PCL particle+MEFs injected (H) hypodermal loose connective tissue.** Thick, white arrow = major, thick fibers; thin, white arrow = minor, thin fibers. Areas of typical fibrin morphology are present in the control and MEF-injected animals (A and B). No areas of typical fibrin morphology are present in ported PCL particle-injected (C) and ported PCL particle+MEFs injected animals (D). Large adipocytes (black arrow), blood vessels (white arrow) and fibroblasts (red arrow) are observed in control and cell injected animals (E and F). PCL particle injected specimens presented with large adipocytes (black arrows) surrounded by smaller adipocytes (green arrows) (G). PCL particle+MEFs injected specimens presented with smaller, but more numerous adipocytes (green arrows) and an infiltration of fibroblasts and/or white blood cells (H). Scale bar in A-D = 200nm; magnification in E, G and H = 40x; magnification in F = 100x.

The histology of the hypodermal region in control mice was as is typically seen in mice and humans i.e. large round adipocytes surrounded by loose connective tissue (Fig 7E). Particles were found amongst adipocytes in both the ported PCL particle and ported PCL particle+MEFs groups. Where only MEFs had been injected, minimal white blood cell infiltration was visible compared to the control (Fig 7F). Injected and resident fibroblasts could not be distinguished. It would have been interesting to determine whether the injected fibroblasts, although syngenic, were still present at the time of sacrifice. Further tests using GFP-labelled cells might allow the injected cells to be traced. In ported PCL particle-injected mice, numerous smaller sized adipocytes were observed in between the larger adipocytes (Fig 7G). Smaller sized adipocytes were also observed in the particles+MEFs treated mice together with the infiltration of white blood cells (Fig 7H). Ported PCL particles were located in the hypodermis.

TEM micrographs of skin biopsies two days after injection identified particles in skin biopsies of both ported PCL particles and particles+MEFs groups, indicating that the particles had successfully been injected in the hypodermis (S6 Fig in S2 File). SEM analysis of fibrin networks in control and MEF-injected mice showed typically thick, major fibers (thick white arrows) and thin, minor fibers (thin, white arrows) (Fig 7A and 7B) as previously observed in healthy mice [27]. In previous studies where inflammation occurred in BALB/c animals, altered fibrin morphology had been noted [28]. During inflammation, the thin, sparingly arranged minor fibrin fibers formed a thick net, intertwined with the major, thick fibers. In severe cases, thick plaques formed. This is also seen in humans during inflammation. In the current study, the morphology of all particle-injected experimental groups was altered, suggesting the presence of systemic inflammation (Fig 7C and 7D). A semi-quantitative estimate revealed the following gradation of severity: control < MEFs < ported PCL particles < ported PCL particles+MEFs.

Blood smears from mice in each of the four experimental groups were evaluated microscopically. Each slide was quantified in terms of monocytes, lymphocytes, eosinophils, basophils and neutrophils. Each slide was analyzed until 100 white blood cells had been counted. Statistical analysis was preformed by means of a one-way ANOVA to determine if any significant difference existed between the four experimental groups at the time of sacrifice (59 days). The ported PCL particle group had a significantly lower monocyte count when compared to the control and MEF groups, while the control and MEF groups had a significantly lower lymphocyte count when compared to the ported PCL particle group. No significant differences were observed between any of the groups for neutrophils, eosinophils or basophils (Table C in S2 File).

### *In vivo* study no. 2: Comparison between ported PCL, non-ported PCL and polystyrene particles

**Fig 8:**
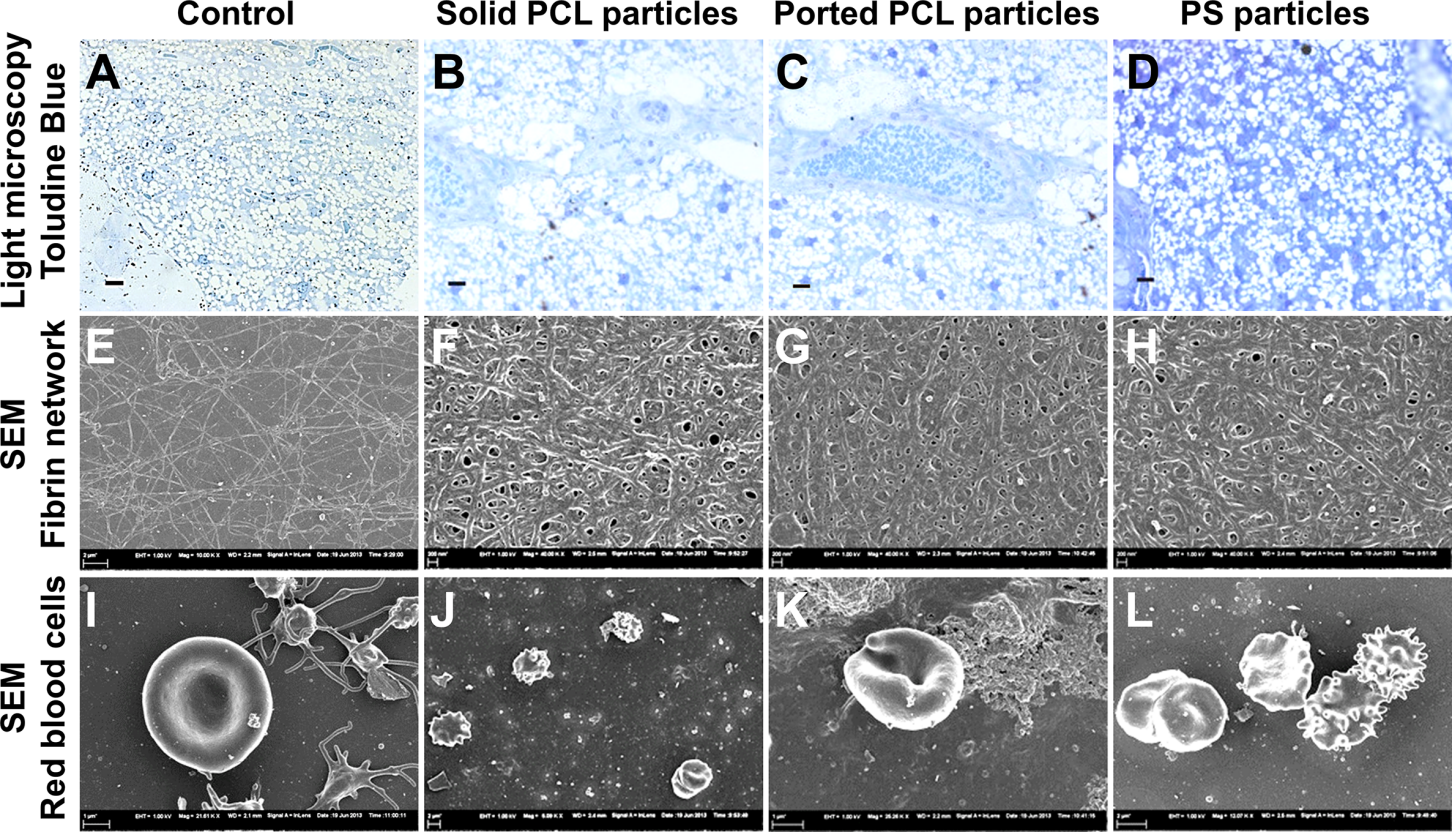
**Light microscopy images (A-D), scanning electron micrographs of fibrin networks (E-H) and red blood cells (I-L) for controls, non-ported PCL particles, ported PCL particles and PS particles in BALB/c mice after three weeks exposure.** Fibrous thickening, leukocyte infiltration and adipocyte formation occurred in the hypodermis in all animals that received particles when compared to controls (A-D). By SEM, a fine fibrin fibre network (E) and healthy red blood cells (I) were observed in controls. However, a thickened and matted fibrin network and changes in red blood cell morphology indicative of inflammation were observed in mice injected with non-ported PCL particles (F and J), ported PCL particles (G and K) and PS particles (H and L). Scale bar in A-D = 50 μm, and in E, J and L = 2 μm, F-H = 200 μm; and in I and K = 1 μm.

A follow-up study was conducted in order to determine whether the inflammation and presence of adipocytes were due to the ported PCL particles or merely the presences of a polymer particle. Light microscopy revealed fibrous thickening in the hypodermal region with infiltration of leukocytes and the presence of smaller adipocytes in all particle injected mice compared to controls (Fig 8A–8D). Fibrin fibers, created when thrombin is added to platelet rich plasma, typically form a fine fiber net (Fig 8E). When whole blood smears are made, discoid erythrocytes are visible with associated platelets (Fig 8I). When fibrin fibers are thickened and matted, this is indicative of inflammation. In whole smears, when inflammation is present, erythrocytes undergo shape changes and fibrin is spontaneously formed between erythrocytes. This is not seen in healthy mice. In the current experiments, from the first week of treatment, matted fibrin, as well as altered RBCs were visible in all animals and all treatment groups, including the inert PS particles Fig 8F–8H and Figs 8J–8L). This is an indication of an inflammatory profile and confirms the LM results.

**Fig 9:**
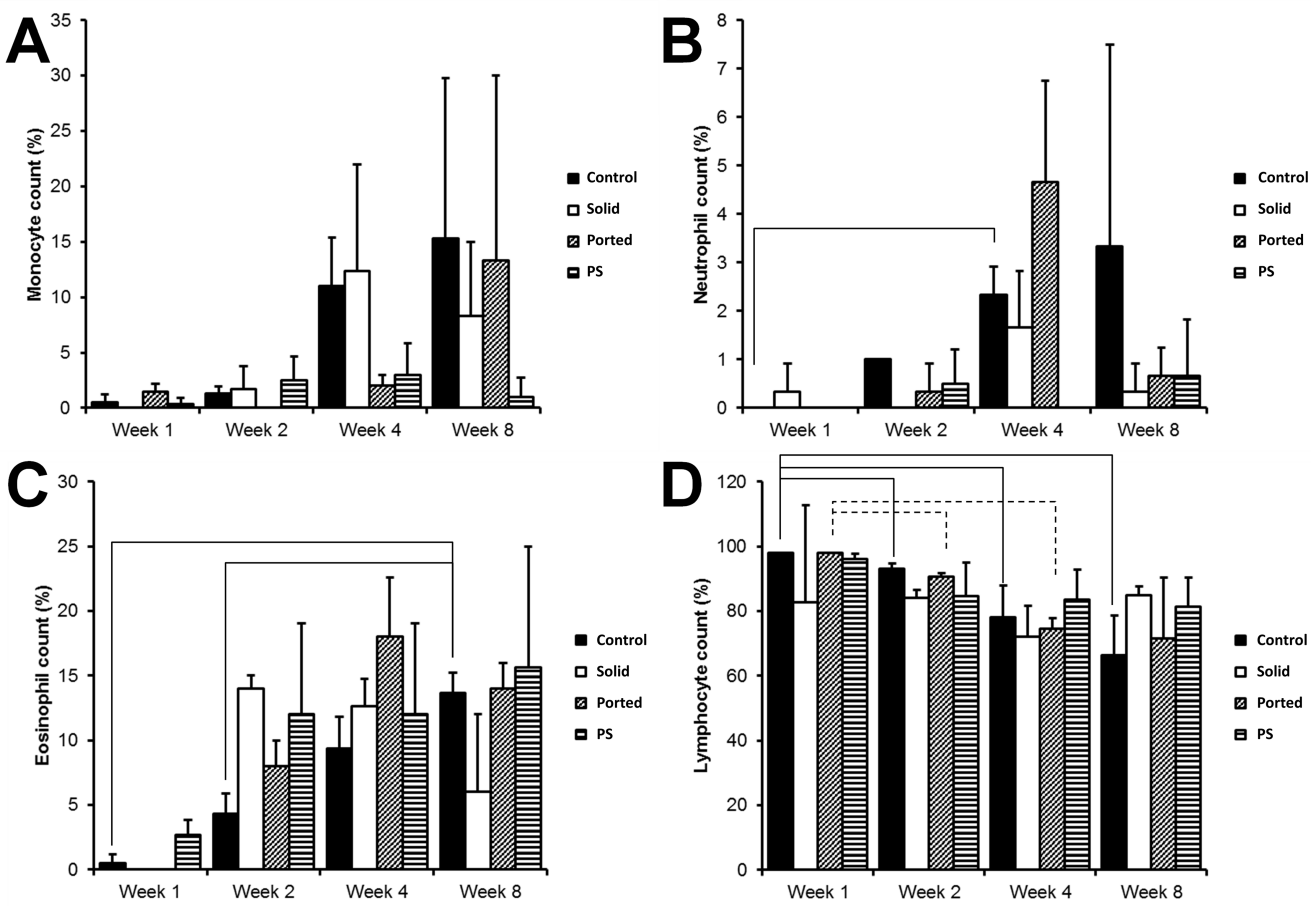
**Monocyte (A), neutrophil (B), eosinophil (C) and lymphocyte (D) profiles at 1, 2, 4 and 8 weeks in mice injected with non-ported PCL, ported PCL and PS particles**. No significant differences were observed between the test groups and the controls at 1, 2, 4 and 8 weeks. Increases in monocyte, neutrophil and eosinophil counts and decreases in lymphocyte counts were seen between week 1 and subsequent time points in all particle injected mice. Connecting lines indicate a *P*-value <0.05.

White blood cell types were recorded as a percentage and plotted over time (weeks 1, 2, 4 and 8). No significant differences were observed between the groups (control, non-ported (solid) PCL, ported PCL and PS) at any of the time points assessed (Fig 9A–9D; basophils not shown). Increases in monocyte, neutrophil and eosinophil counts were observed between week 1 and later weeks, with a decrease in lymphocyte counts; no trend was observed for basophils. Most of the changes did not reach statistical significance; those that did as well as the observed trends are shown in Fig 9.

The overall conclusion is that local and systemic inflammation were present from week 1 in all mice that received particles; this included both solid and ported PCL particles as well as the apparently inert PS particles. Inflammation was evident from light microscopy and also by scanning electron microscopy of fibrin clots and erythrocytes: thickened and matted fibrin mats and erythrocyte shape changes indicative of inflammation were observed.

## Discussion

Apart from its tissue-filling requirement, the ideal tissue filler needs to have minimal adverse effects. Thus, the most important aspect when dealing with tissue fillers is their safety and biocompatibility. Biocompatibility can be achieved with careful design, concentrating on chemical composition, surface structure, surface charge and particle size [29]. Cell-material interactions can be modulated. When an inert surface is designed, it does not allow for adsorption of proteins and adhesion of cells [30]. It aims to prevent immune response activation and any interaction between the material and the surrounding environment. Such a design is biocompatible; however, it applies to more permanent synthetic replacement of damaged tissues. For co-transplantation of cells with cell delivery vehicles, materials promoting cell attachment, migration, proliferation, differentiation, long-term viability and cell functioning are more appropriate. This kind of delivery vehicle aims at creating “hybrid bioartificial organs” for tissue engineering [30].

Ported PCL particles are made from the biodegradable polymer polycaprolactone which has a favourable resorption profile and is biocompatible. A benefit of ported PCL particles is their longer lasting resorbable tissue filler effect and their novel hollow ported architecture [18]. These hollow ports have been shown in a previous study to act as a sheltered environment, protecting cells from stress experienced during *in vivo* transplantation, as well as creating an anchor point which is conducive to cell expansion [16, 31]. Both fluorescent microscopy and SEM of ported PCL particles co-cultured with cells showed that cells do attach to these particles. Where attachment did occur, it resulted in clumping of the particles. However, in the present study we did not observe adhesion and/or migration of cells into the inside of the hollow particles which is required for the “tissue harbour” effect. This, together with the particle aggregation might be attributed to the incubation procedure used. Refinement of this procedure to ultimately obtain a monolayer of cells on the particle surface as well as cells within the tissue harbour remains a key objective.

In the series of experiments described herein, *in vitro* studies were conducted to evaluate the effect of ported PCL particles on co-cultured MEFs. Dermal fibroblasts have been used as an autogeneic filler; however embryonic fibroblasts may have the advantage of being younger and would therefore be expected to contribute to more efficient regenerative capacity *in vivo*. During initial cell culturing, both MEFs and ASFs demonstrated a healthy spindle-shaped morphology characteristic of normal fibroblasts. No significant differences in cell numbers/growth rates were observed between these two cell types. MEFs were chosen for all further experiments.

The issue under investigation is whether ported PCL particles are toxic to cells. The lack of PI staining, the absence of a statistically significant difference in LDH levels or an increase in sub-G_1_ MEFs cultured together with ported PCL particles suggests that the particles had no negative effects on MEFs. In addition, no significant difference was observed in cell division and mitotic activity between the cells only group and cells with particles group. As no major limitations were found *in vitro*, an *in vivo* study was initiated to assess the effect in mice injected subcutaneously with the different cell, particle and cell-particle preparations.

During the first *in vivo* experiment involving ported PCL particles and cells, visible symptoms of acute inflammation were generally not present on the skin surface although scratching at the site of injection was observed initially in more than 50% of the mice in each experimental group. Even the controls injected with sterile PBS scratched, suggesting that there was no correlation between scratching and inflammation-induced irritation. No pus was visible at the injection sites, but the MEF and particles+MEF groups presented with some swelling on the dorsal side. No major differences in white blood cell differential counts were observed.

During blood clotting, thrombin cleaves fibrinogen into two peptides called fibrinopeptides, which form fibrin monomers. It is the assembly of these monomers which forms the fibrin network, and this can be studied at the ultrastructural level [32]. A fibrin clot is induced experimentally by the addition of thrombin to platelet rich plasma, resulting in an expansive, fully coagulated layer of fibrin fibers. In previous studies, it was noted that inflammation alters fibrin network formation. These changes are visible by SEM. Typically, fibrin networks change to form thickened layers, have a netted appearance, or form thickened plates. The murine BALB/c asthma model is one model that has previously been used successfully for a number of *in vivo* immunological applications and for testing novel therapeutics [28]. These authors were the first to use this model to study fibrin ultrastructure and changes in platelet ultrastructure during asthma.

In the current study, fibrin networks displayed an altered morphology. The changes are similar to those seen in BALB/c mice where inflammation was induced [28]. During inflammation, the thin, sparingly arranged minor fibrin fibres formed a thick net, intertwined with the major, thick fibres. In severe cases, thick plaques are formed. This is also seen in humans during an ongoing inflammatory process. In the current study, the fibrin morphology of all experimental groups changed, from a neat expanse of thick and thin fibres to areas of matting, indicating the presence of inflammation. This suggests that the injection of foreign particles triggered an inflammatory state in the mice. The fact that these characteristic changes were seen when preparing fibrin clots from blood, suggests the trigger is systemic inflammation.

The particles were found to be located in the hypodermis and were surrounded by adipocytes. The control and MEF-injected animals showed a typical morphology with large round fat cells. A large number of particles were embedded among adipocytes in both the ported PCL particle and ported PCL particle+MEFs groups. Infiltration of leukocytes was observed in all particle-injected animals, confirming the presence of an inflammatory reaction. Numerous smaller sized adipocytes were observed in-between the larger adipocytes in PCL and PS particle injected animals. Fibrous thickening was also observed in the hypodermis in the PCL (solid and ported) and PS particle injected mice. These results indicate that all of the injected particle types induce inflammation at the site of injection and also induce adipocyte formation.

In conclusion, no significant effects were observed *in vitro* in terms of cell viability, growth and morphology of MEFs grown with ported PCL particles, indicating that the particles are likely to be suitable as cell delivery systems. *In vivo* studies revealed that the particles caused an inflammatory response and an increase in adipocyte numbers. Whether this is beneficial in the clinical setting remains to be determined; increased inflammation and associated adipogenesis might contribute to dermal filling and tightening. Future studies might benefit from using mesenchymal stromal/stem cells (MSCs) attached to the PCL particles. MSCs are able to differentiate into adipocytes and it is known that the activation and proliferation of adipocyte stem cells are important for the long term effects of tissue bulking agents [33]. Furthermore, MSCs are known to secrete a large spectrum of bioactive molecules that are immunosuppressive, and have been successfully applied in microsphere-based tissue engineering [34, 35].

## Supporting information

S1 File Figure A: Chronic inflammation in the test animals over the trial period. Figure B: Acute inflammation in the test animals over the trial period. Figure C: Tissue necrosis in the test animals over the trial period. Figure D: Fibrosis in the test animals over the trial period. Figure E: Granulomatous/foreign body response in the test animals over the trial period. Figure F: Representative TEMs of skin biopsies of particles group (A) and particles+MEFs group (B) in the in vivo experiment injecting particles+MEFs. Particles could be identified in skin biopsies of both the particles and particles+MEFs groups. The aim of the TEM investigation was to determine if any cells could be detected inside the particles. No cells were present inside the particles in either group. These results reflect the conclusion that was made after the light microscopy study, indicating that cells did not migrate into the ported PCL particles. Bar in A = 5μm and in B = 10μm.
(ZIP)

S2 File In vitro and in vivo data. Table A: Groups of rats used in the biotoxicity trial. Table B: Observations on mice in the *in vivo* experiment assessing the effect of ported PCL particles and cells. Table C: Statistical comparisons preformed between the various white blood cell types assessed from blood smears of experimental mice injected with ported PCL particles with or without MEFs. Table D: Schedule of the *in vivo* experiment assessing the effect of ported and non-ported PCL as well as polystyrene (PS) particles. Table E: Overview of the animals, tests and procedures performed in the *in vivo* experiment assessing the effect of ported and non-ported PCL as well as polystyrene (PS) particles in BALB/c mice.
(DOCX)

S3 File All data underlying the findings of the study. (ZIP)

## References

[1] Kulichova D, Borovaya A, Ruzicka T, Thomas P, Gauglitz GG. Understanding the safety and tolerability of facial filling therapeutics. Expert opinion on drug safety. 2014:1–12. Epub 2014/07/19. doi: 10.1517/14740338.2014.939168 . 25036318 10.1517/14740338.2014.939168

[2] Funt D, Pavicic T. Dermal fillers in aesthetics: an overview of adverse events and treatment approaches. Clinical, cosmetic and investigational dermatology. 2013;6:295–316. Epub 2013/12/24. doi: 10.2147/CCID.S50546 ; PubMed Central PMCID: PMCPmc3865975. 24363560 10.2147/CCID.S50546 PMC3865975

[3] ASPS. 2012 Plastic Surgery Procedural Statistics2012 15/07/2014. Available from: http://www.plasticsurgery.org/Documents/news-resources/statistics/2012-Plastic-Surgery-Statistics/full-plastic-surgery-statistics-report.pdf.

[4] Gilbert E, Calvisi L. Midface and perioral volume restoration: a conversation between the US and Italy. Journal of drugs in dermatology: JDD. 2014;13(1):67–74. Epub 2014/01/05. . 24385122

[5] De Boulle K. Management of complications after implantation of fillers. J Cosmet Dermatol. 2004;3(1):2–15. Epub 2006/12/14. JCD058 [pii] doi: 10.1111/j.1473-2130.2004.00058.x . 17163941 10.1111/j.1473-2130.2004.00058.x

[6] De Boulle K, Glogau R, Kono T, Nathan M, Tezel A, Roca-Martinez JX, et al A review of the metabolism of 1,4-butanediol diglycidyl ether-crosslinked hyaluronic acid dermal fillers. Dermatol Surg. 2013;39(12):1758–66. Epub 2013/08/15. doi: 10.1111/dsu.12301 . 23941624 10.1111/dsu.12301 PMC4264939

[7] Wollina U, Goldman A. Dermal fillers: facts and controversies. Clinics in dermatology. 2013;31(6):731–6. Epub 2013/10/29. doi: 10.1016/j.clindermatol.2013.05.010 . 24160278 10.1016/j.clindermatol.2013.05.010

[8] Ramos ESM, Fonteles LA, Lagalhard CS, Fucci-da-Costa AP. STYLAGE(R): a range of hyaluronic acid dermal fillers containing mannitol. Physical properties and review of the literature. Clinical, cosmetic and investigational dermatology. 2013;6:257–61. Epub 2013/11/05. doi: 10.2147/CCID.S35251 ; PubMed Central PMCID: PMCPmc3810198. 24187508 10.2147/CCID.S35251 PMC3810198

[9] Ammala A. Biodegradable polymers as encapsulation materials for cosmetics and personal care markets. International journal of cosmetic science. 2013;35(2):113–24. doi: 10.1111/ics.12017 23075204 10.1111/ics.12017

[10] Tran C, Carraux P, Micheels P, Kaya G, Salomon D. In vivo bio-integration of three hyaluronic acid fillers in human skin: a histological study. Dermatology (Basel, Switzerland). 2014;228(1):47–54. Epub 2014/02/08. doi: 10.1159/000354384 . 24503674 10.1159/000354384

[11] Huang X, Liang Y, Li Q. Safety and efficacy of hyaluronic acid for the correction of nasolabial folds: a meta-analysis. European journal of dermatology: EJD. 2013;23(5):592–9. Epub 2013/11/10. doi: 10.1684/ejd.2013.2151 . 24200955 10.1684/ejd.2013.2151

[12] Alijotas-Reig J, Fernandez-Figueras MT, Puig L. Inflammatory, immune-mediated adverse reactions related to soft tissue dermal fillers. Semin Arthritis Rheum. 2013;43(2):241–58. Epub 2013/05/07. doi: 10.1016/j.semarthrit.2013.02.001 S0049-0172(13)00021-8 [pii]. . 23642806 10.1016/j.semarthrit.2013.02.001

[13] Doppalapudi S, Jain A, Khan W, Domb AJ. Biodegradable polymers—an overview. Polymers for Advanced Technologies. 2014;25(5):427–35.

[14] Dash TK, Konkimalla VB. Poly-small je, Ukrainian-caprolactone based formulations for drug delivery and tissue engineering: A review. J Control Release. 2012;158(1):15–33. Epub 2011/10/04. doi: 10.1016/j.jconrel.2011.09.064 S0168-3659(11)00849-2 [pii]. . 21963774 10.1016/j.jconrel.2011.09.064

[15] Chen DR, Bei JZ, Wang SG. Polycaprolactone microparticles and their biodegradation. Polymer Degradation and Stability. 2000;67(3):455–9.

[16] Steynberg T, Visagie M, Mqoco T, Idicula A, Moolman S, Richter W, et al Qualitative assessment of smooth muscle cells propagated on 2D-and 3D-polycaprolactone polymers via scanning electron microscope. Biomedical Research (0970-938X). 2012;23(2).

[17] Figueiredo VM. A five-patient prospective pilot study of a polycaprolactone based dermal filler for hand rejuvenation. J Cosmet Dermatol. 2013;12(1):73–7. Epub 2013/02/27. doi: 10.1111/jocd.12020 . 23438145 10.1111/jocd.12020

[18] Naidoo K, Rolfes H, Easton K, Moolman S, Chetty A, Richter W, et al An emulsion preparation for novel micro-porous polymeric hemi-shells. Materials Letters. 2008;62(2):252–4.

[19] Freshney R. Animal cell cultures 3rd ed: Oxford: URL Press; 1995.

[20] Strober W. Trypan blue exclusion test of cell viability. Current protocols in immunology. 2001:A. 3B. 1–A. 3B. 2. 10.1002/0471142735.ima03bs21 18432654

[21] Gillies RJ, Didier N, Denton M. Determination of cell number in monolayer cultures. Analytical biochemistry. 1986;159(1):109–13. . 3812988 10.1016/0003-2697(86)90314-3

[22] Kueng W, Silber E, Eppenberger U. Quantification of cells cultured on 96-well plates. Analytical biochemistry. 1989;182(1):16–9. . 2604040 10.1016/0003-2697(89)90710-0

[23] Allen M, Millett P, Dawes E, Rushton N. Lactate dehydrogenase activity as a rapid and sensitive test for the quantification of cell numbers in vitro. Clinical materials. 1994;16(4):189–94. . 10150166 10.1016/0267-6605(94)90116-3

[24] Krishan A. Rapid flow cytofluorometric analysis of mammalian cell cycle by propidium iodide staining. The Journal of cell biology. 1975;66(1):188–93. 49354 10.1083/jcb.66.1.188 PMC2109516

[25] Danz R, Vogelgsang A, Käthner R. PlasDIC—a useful modification of the differential interference contrast according to Smith/Nomarski in transmitted light arrangement. 2004.

[26] Hayes TL, Pease RF. The scanning electron microscope: principles and applications in biology and medicine. Advances in biological and medical physics. 1968;12:85–137. . 4879503 10.1016/b978-1-4831-9928-3.50006-0

[27] Pretorius E, Ekpo O, Smit E. Comparative ultrastructural analyses of platelets and fibrin networks using the murine model of asthma. Experimental and Toxicologic Pathology. 2007;59(2):105–14. doi: 10.1016/j.etp.2007.02.011 17600694 10.1016/j.etp.2007.02.011

[28] Pretorius E, Ekpo OE, Smit E. Comparative ultrastructural analyses of platelets and fibrin networks using the murine model of asthma. Experimental and Toxicologic Pathology. 2007;59(2):105–14. doi: 10.1016/j.etp.2007.02.011 17600694 10.1016/j.etp.2007.02.011

[29] Laeschke K. Biocompatibility of microparticles into soft tissue fillers. Seminars in Cutaneous Medicine and Surgery. 2004;23(4):214–7. doi: 10.1016/j.sder.2004.09.005 15745227 10.1016/j.sder.2004.09.005

[30] Bacáková L, Filová E, Rypácek F, Svorcík V, Starý V. Cell adhesion on artificial materials for tissue engineering. Physiol Res. 2004;53 Suppl 1(0862–8408). 15119934

[31] Steynberg T. Comparison of the *in vitro* effect of two-dimensional and three-dimensional polycaprolactone polymers on cell morphology, viability and cytotoxici ty. Pretoria: University of Pretoria; 2010.

[32] Vine AK. Recent advances in haemostasis and thrombosis. Retina. 2009;29(1):1–7. doi: 10.1097/IAE.0b013e31819091dc 19050668 10.1097/IAE.0b013e31819091dc

[33] Kruglikov IL, Wollina U. Soft tissue fillers as non‐specific modulators of adipogenesis: change of the paradigm? Experimental dermatology. 2015;24(12):912–5. doi: 10.1111/exd.12852 26309229 10.1111/exd.12852

[34] Caplan AI. Adult mesenchymal stem cells for tissue engineering versus regenerative medicine. J Cell Physiol. 2007;213(2):341–7. Epub 2007/07/11. doi: 10.1002/jcp.21200 . 17620285 10.1002/jcp.21200

[35] Huang S, Lu G, Wu Y, Jirigala E, Xu Y, Ma K, et al Mesenchymal stem cells delivered in a microsphere-based engineered skin contribute to cutaneous wound healing and sweat gland repair. J Dermatol Sci. 2012;66(1):29–36. Epub 2012/03/09. doi: 10.1016/j.jdermsci.2012.02.002 nS0923-1811(12)00051-5 [pii]. . 22398148 10.1016/j.jdermsci.2012.02.002

