# Supplementary figures and images for: An in vitro and in vivo study on the properties of hollow polycaprolactone cell-delivery particles
Source: PLoS One. 2018 Jul 3;13(7):e0198248. doi: 10.1371/journal.pone.0198248 (PMC6029779; doi:10.1371/journal.pone.0198248)

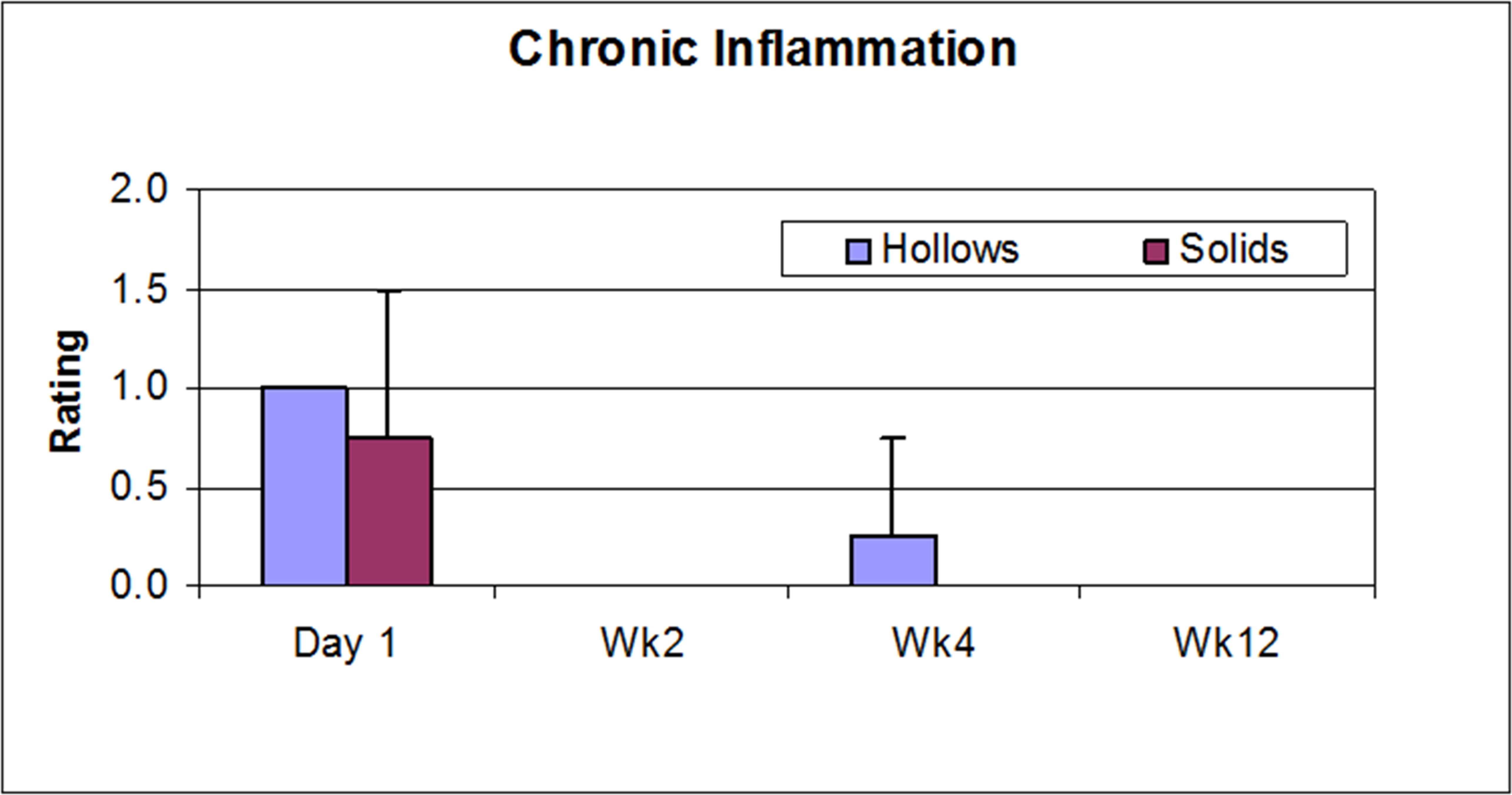

Supplement: S1 File — Figure A: Chronic inflammation in the test animals over the trial period. Figure B: Acute inflammation in the test animals over the trial period. Figure C: Tissue necrosis in the test animals over the trial period. Figure D: Fibrosis in the test animals over the trial period. Figure E: Granulomatous/foreign body response in the test animals over the trial period. Figure F: Representative TEMs of skin biopsies of particles group (A) and particles+MEFs group (B) in the in vivo experiment injecting particles+MEFs. Particles could be identified in skin biopsies of both the particles and particles+MEFs groups. The aim of the TEM investigation was to determine if any cells could be detected inside the particles. No cells were present inside the particles in either group. These results reflect the conclusion that was made after the light microscopy study, indicating that cells did not migrate into the ported PCL particles. Bar in A = 5μm and in B = 10μm. (ZIP) [file pone.0198248.s001.zip › Fig A.tiff]

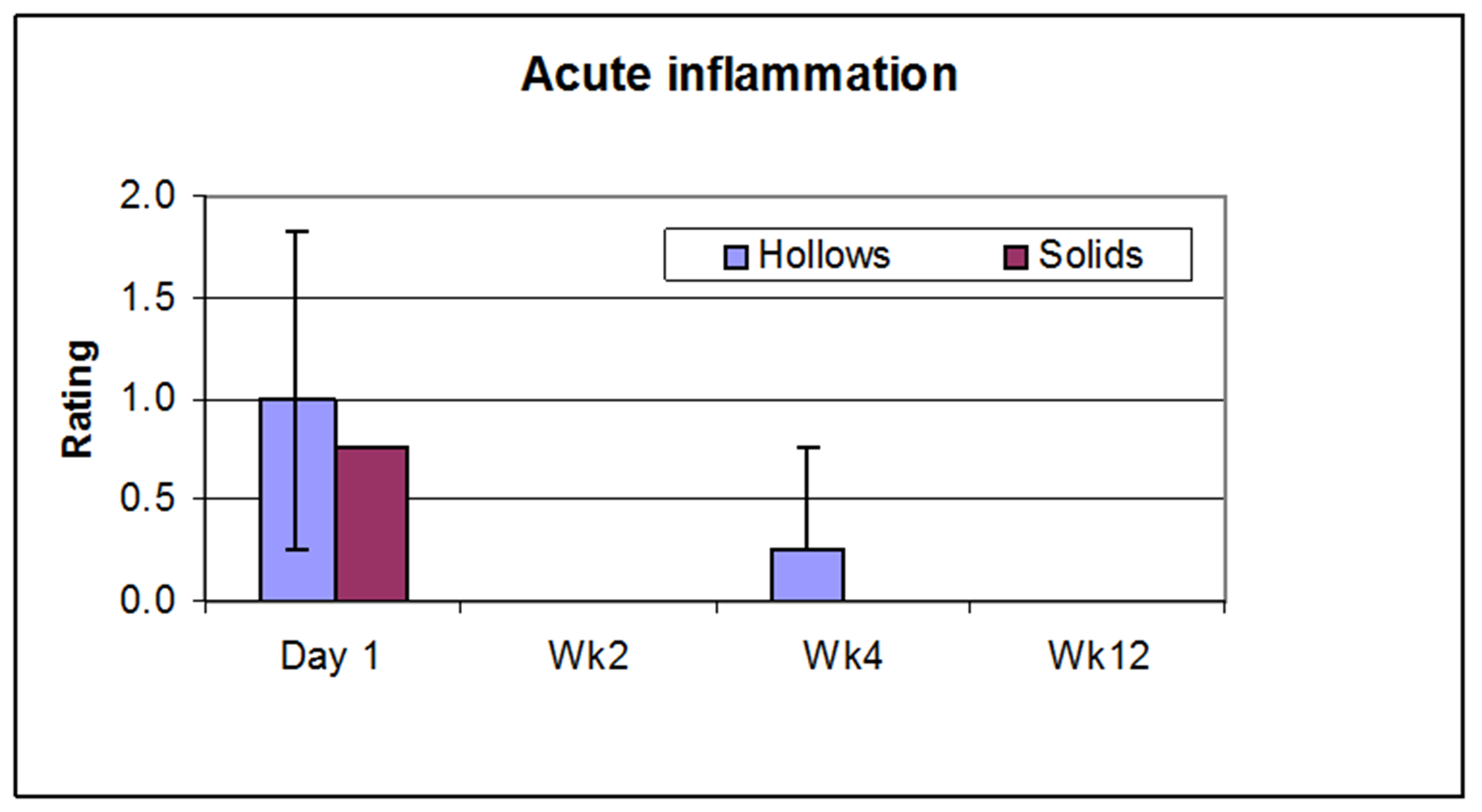

Supplement: S1 File — Figure A: Chronic inflammation in the test animals over the trial period. Figure B: Acute inflammation in the test animals over the trial period. Figure C: Tissue necrosis in the test animals over the trial period. Figure D: Fibrosis in the test animals over the trial period. Figure E: Granulomatous/foreign body response in the test animals over the trial period. Figure F: Representative TEMs of skin biopsies of particles group (A) and particles+MEFs group (B) in the in vivo experiment injecting particles+MEFs. Particles could be identified in skin biopsies of both the particles and particles+MEFs groups. The aim of the TEM investigation was to determine if any cells could be detected inside the particles. No cells were present inside the particles in either group. These results reflect the conclusion that was made after the light microscopy study, indicating that cells did not migrate into the ported PCL particles. Bar in A = 5μm and in B = 10μm. (ZIP) [file pone.0198248.s001.zip › Fig B.tiff]

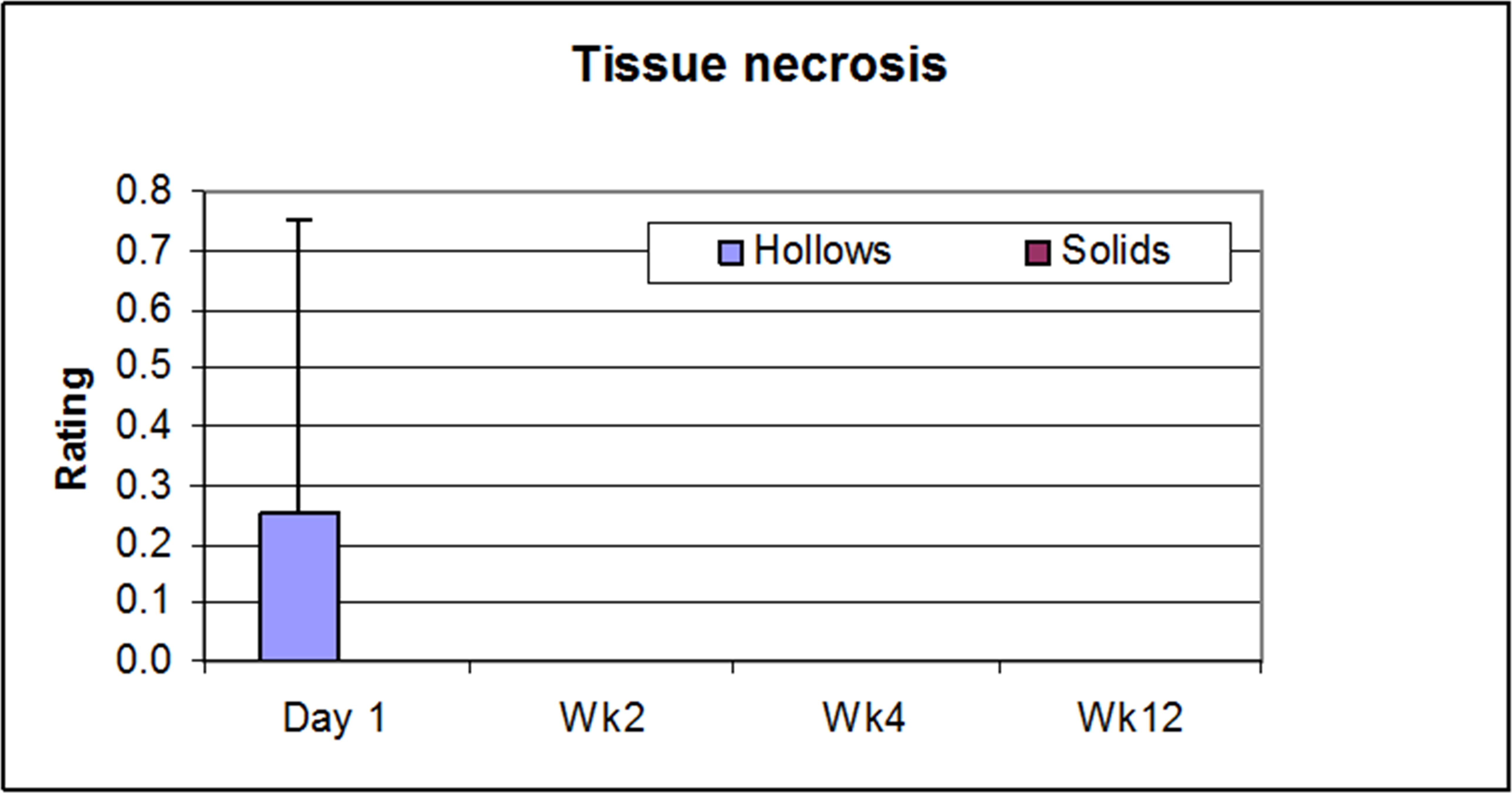

Supplement: S1 File — Figure A: Chronic inflammation in the test animals over the trial period. Figure B: Acute inflammation in the test animals over the trial period. Figure C: Tissue necrosis in the test animals over the trial period. Figure D: Fibrosis in the test animals over the trial period. Figure E: Granulomatous/foreign body response in the test animals over the trial period. Figure F: Representative TEMs of skin biopsies of particles group (A) and particles+MEFs group (B) in the in vivo experiment injecting particles+MEFs. Particles could be identified in skin biopsies of both the particles and particles+MEFs groups. The aim of the TEM investigation was to determine if any cells could be detected inside the particles. No cells were present inside the particles in either group. These results reflect the conclusion that was made after the light microscopy study, indicating that cells did not migrate into the ported PCL particles. Bar in A = 5μm and in B = 10μm. (ZIP) [file pone.0198248.s001.zip › Fig C.tiff]

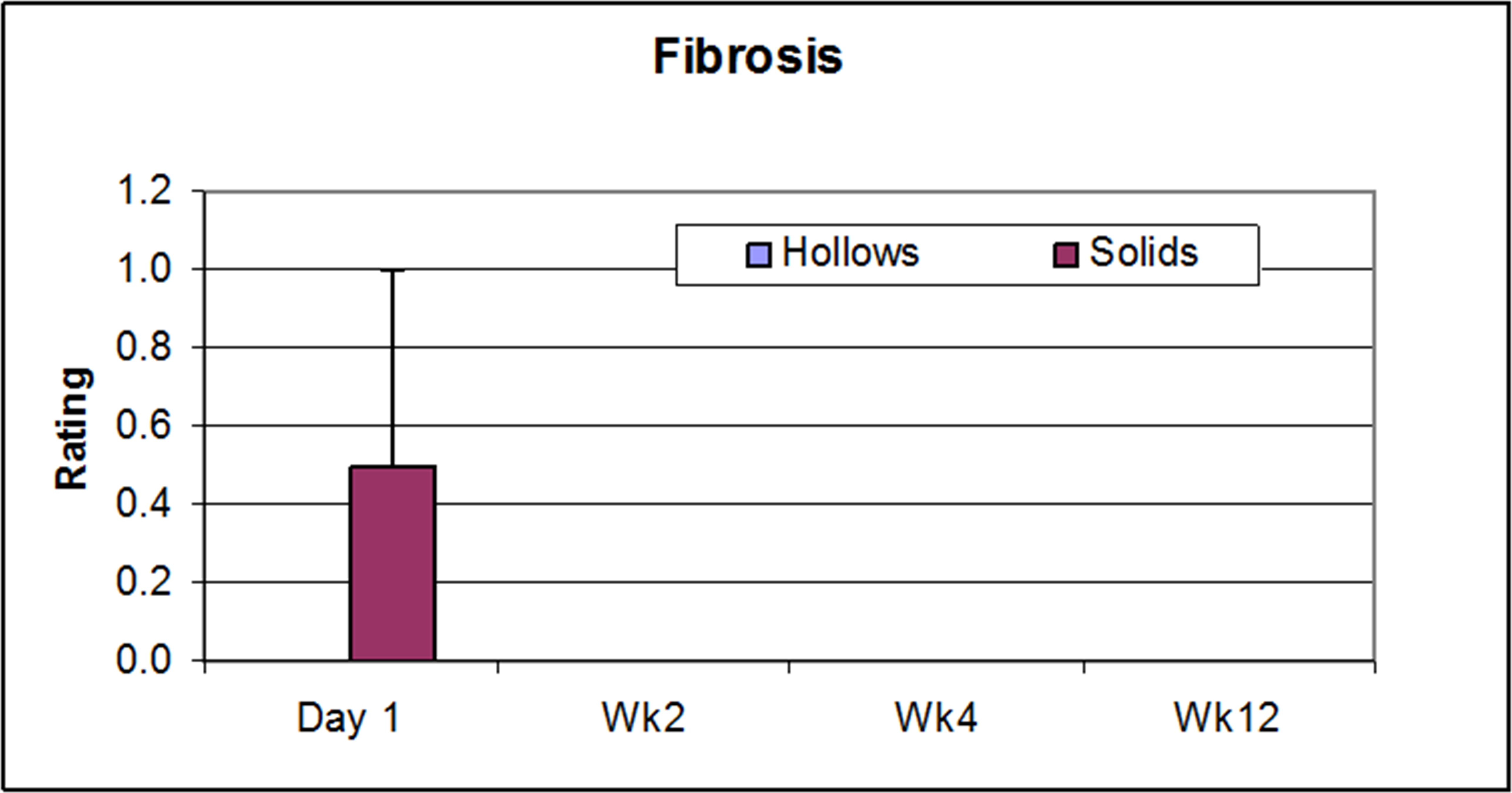

Supplement: S1 File — Figure A: Chronic inflammation in the test animals over the trial period. Figure B: Acute inflammation in the test animals over the trial period. Figure C: Tissue necrosis in the test animals over the trial period. Figure D: Fibrosis in the test animals over the trial period. Figure E: Granulomatous/foreign body response in the test animals over the trial period. Figure F: Representative TEMs of skin biopsies of particles group (A) and particles+MEFs group (B) in the in vivo experiment injecting particles+MEFs. Particles could be identified in skin biopsies of both the particles and particles+MEFs groups. The aim of the TEM investigation was to determine if any cells could be detected inside the particles. No cells were present inside the particles in either group. These results reflect the conclusion that was made after the light microscopy study, indicating that cells did not migrate into the ported PCL particles. Bar in A = 5μm and in B = 10μm. (ZIP) [file pone.0198248.s001.zip › Fig D.tiff]

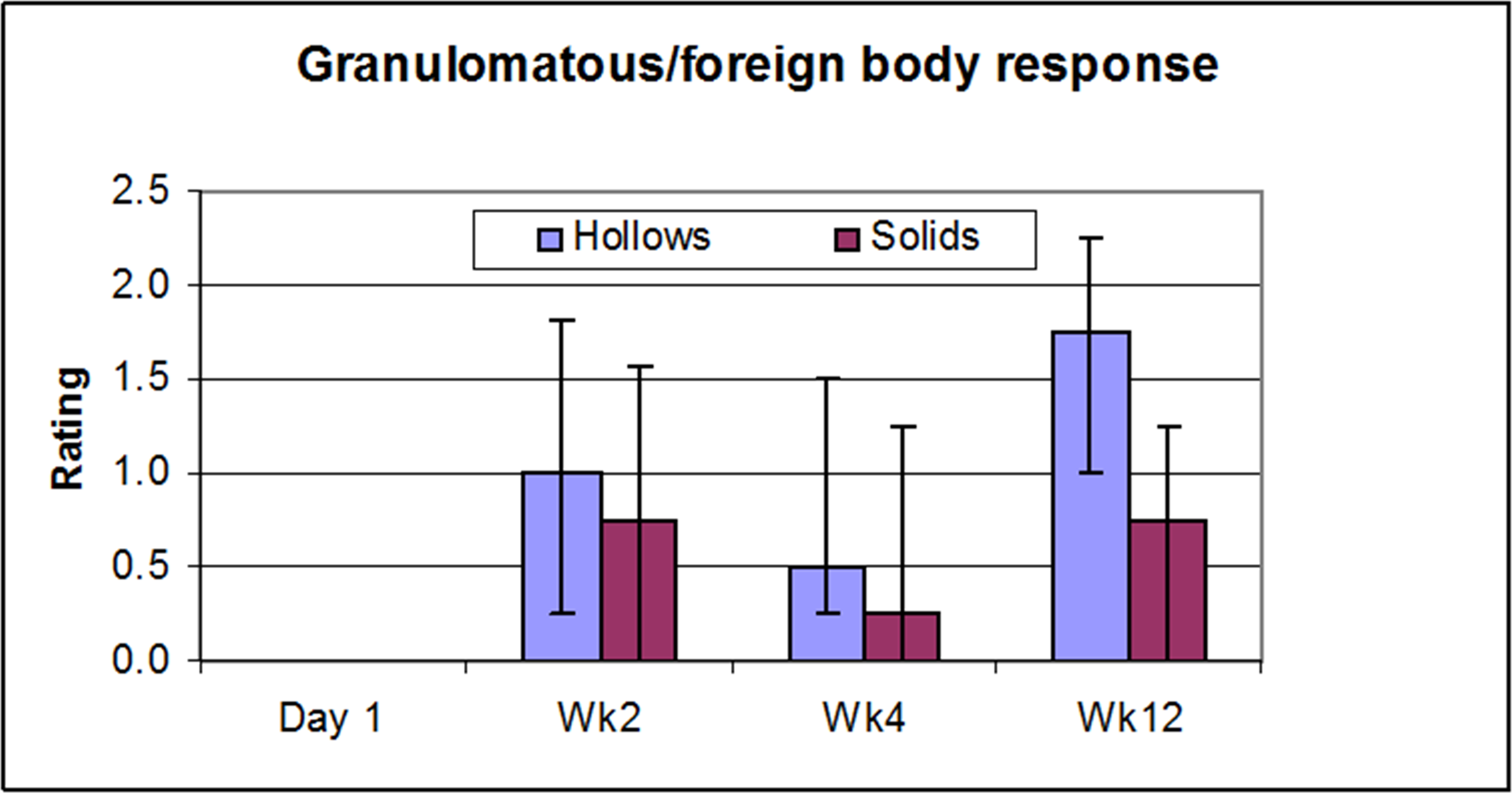

Supplement: S1 File — Figure A: Chronic inflammation in the test animals over the trial period. Figure B: Acute inflammation in the test animals over the trial period. Figure C: Tissue necrosis in the test animals over the trial period. Figure D: Fibrosis in the test animals over the trial period. Figure E: Granulomatous/foreign body response in the test animals over the trial period. Figure F: Representative TEMs of skin biopsies of particles group (A) and particles+MEFs group (B) in the in vivo experiment injecting particles+MEFs. Particles could be identified in skin biopsies of both the particles and particles+MEFs groups. The aim of the TEM investigation was to determine if any cells could be detected inside the particles. No cells were present inside the particles in either group. These results reflect the conclusion that was made after the light microscopy study, indicating that cells did not migrate into the ported PCL particles. Bar in A = 5μm and in B = 10μm. (ZIP) [file pone.0198248.s001.zip › Fig E.tiff]

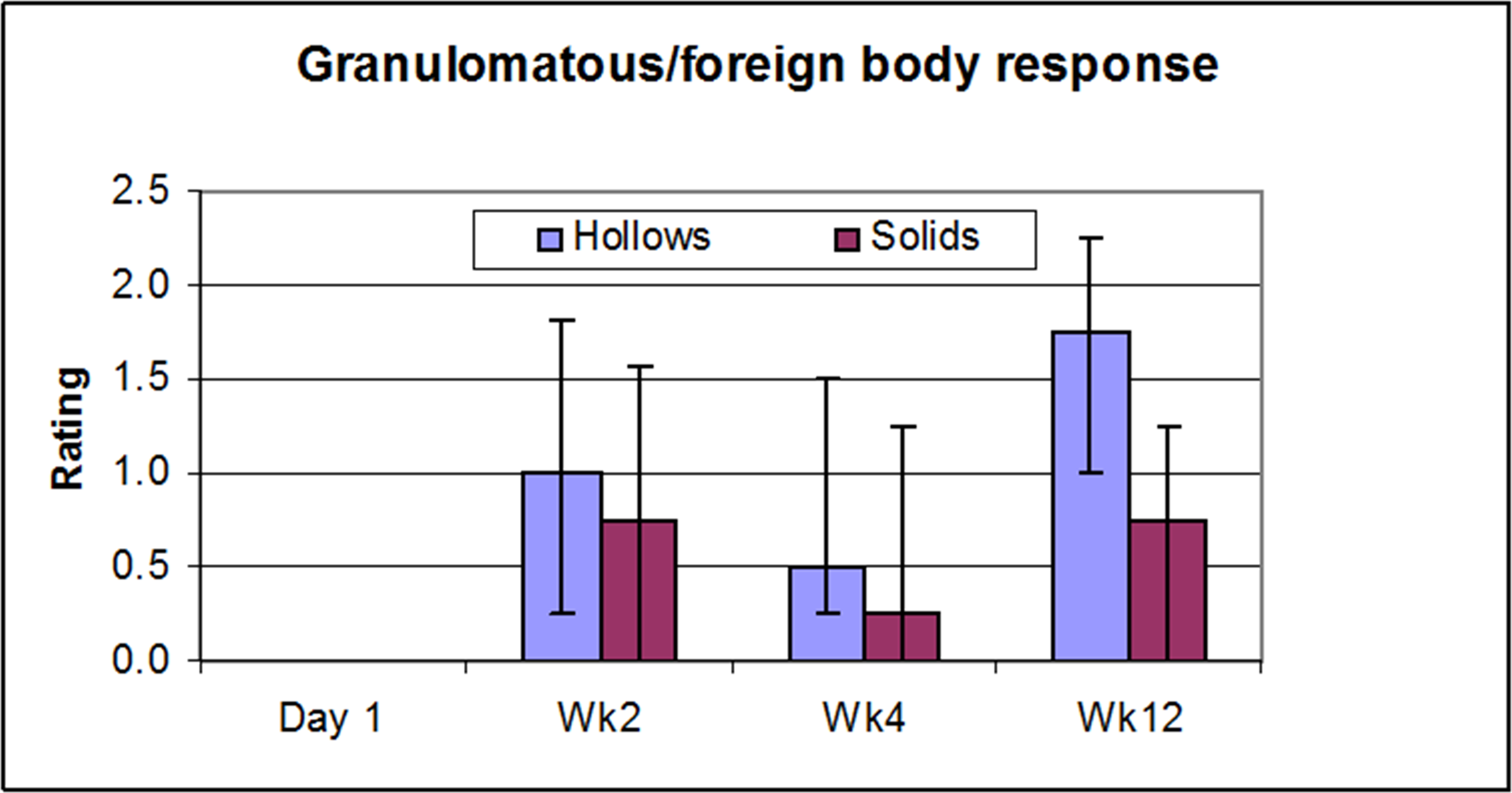

Supplement: S1 File — Figure A: Chronic inflammation in the test animals over the trial period. Figure B: Acute inflammation in the test animals over the trial period. Figure C: Tissue necrosis in the test animals over the trial period. Figure D: Fibrosis in the test animals over the trial period. Figure E: Granulomatous/foreign body response in the test animals over the trial period. Figure F: Representative TEMs of skin biopsies of particles group (A) and particles+MEFs group (B) in the in vivo experiment injecting particles+MEFs. Particles could be identified in skin biopsies of both the particles and particles+MEFs groups. The aim of the TEM investigation was to determine if any cells could be detected inside the particles. No cells were present inside the particles in either group. These results reflect the conclusion that was made after the light microscopy study, indicating that cells did not migrate into the ported PCL particles. Bar in A = 5μm and in B = 10μm. (ZIP) [file pone.0198248.s001.zip › Fig F.tiff]

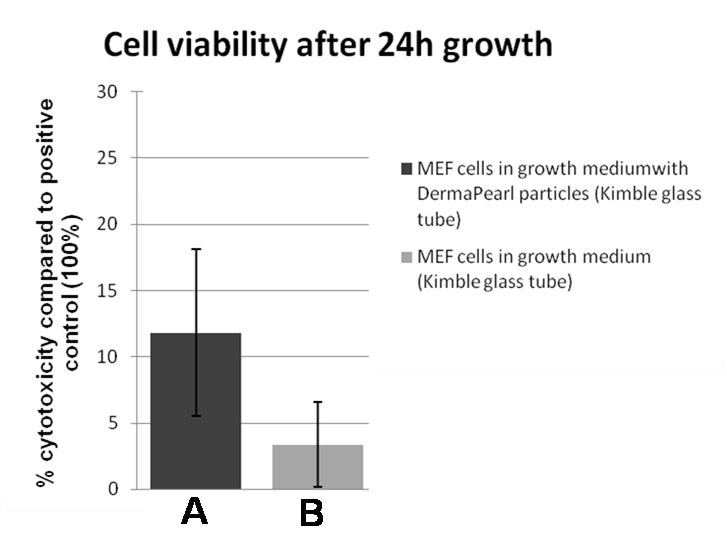

Supplement: S3 File — (ZIP) [file pone.0198248.s003.zip › In Vitro/LDH/LDH no 24-well.png]

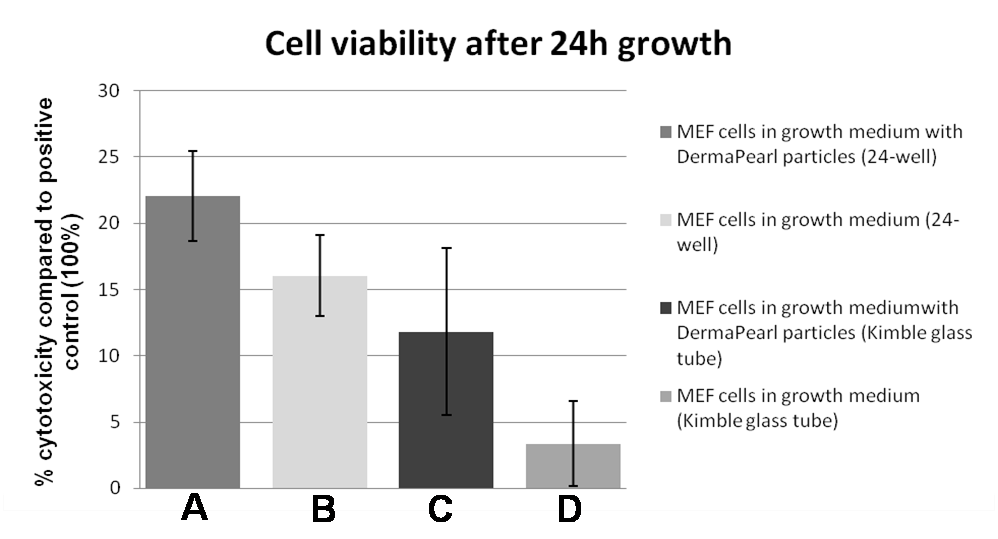

Supplement: S3 File — (ZIP) [file pone.0198248.s003.zip › In Vitro/LDH/LDH.png]
