# Supplementary material for: An in vitro and in vivo study on the properties of hollow polycaprolactone cell-delivery particles
Source: PLoS One. 2018 Jul 3;13(7):e0198248. doi: 10.1371/journal.pone.0198248 (PMC6029779; doi:10.1371/journal.pone.0198248)
Supplement: S2 File — Table A: Groups of rats used in the biotoxicity trial. Table B: Observations on mice in the in vivo experiment assessing the effect of ported PCL particles and cells. Table C: Statistical comparisons preformed between the various white blood cell types assessed from blood smears of experimental mice injected with ported PCL particles with or without MEFs. Table D: Schedule of the in vivo experiment assessing the effect of ported and non-ported PCL as well as polystyrene (PS) particles. Table E: Overview of the animals, tests and procedures performed in the in vivo experiment assessing the effect of ported and non-ported PCL as well as polystyrene (PS) particles in BALB/c mice. (DOCX) [file pone.0198248.s002.docx]

**S1 File**

**A. Rat biotoxicity trial**

**Objectives**

To assess histologically, the short term extent of inflammation and other changes.

**Materials and methods**

Model system

- Animal model: male, Sprague Dawley rats (>250g).
- PCL microparticles in saline carrier medium.

Experimental design

- An acute toxicity study was performed using 24 female Sprague Dawley rats (>250g).
- Each animal received 2 intramuscular injections (1cc) of either saline only (**control**) or PCL microparticles in saline carrier medium (**experiment**): one in the right hand side gluteal muscle tissue, and the other in the left hand side gluteal muscle tissue.
- The animals were divided into 6 groups of 4 rats each according to termination time and injected solution.
- Termination times of 1 day, 2 weeks, 4 weeks and 12 weeks were chosen to investigate both the short- and long-term processes and responses.
- Groups 1,2,4 & 6 are the **experimental** groups, groups 3 & 5 were the **control** groups. (S1 Table)
- **Table A: Groups of rats used in the biotoxicity trial.**

| **Group** | **Injected Solution** | **Termination Time** |
| --- | --- | --- |
| 1 | Left gluteal muscle: Solids in saline  Right gluteal muscle: Hollows in saline | 1 day |
| 2 | Left gluteal muscle: Solids in saline  Right gluteal muscle: Hollows in saline | 2 weeks |
| 3 | Left gluteal muscle: Saline (control)  Right gluteal muscle: Saline (control) | 2 weeks |
| 4 | Left gluteal muscle: Solids in saline  Right gluteal muscle: Hollows in saline | 4 weeks |
| 5 | Left gluteal muscle: Saline (control)  Right gluteal muscle: Saline (control | 4 weeks |
| 6 | Left gluteal muscle: Solids in saline  Right gluteal muscle: Hollows in saline | 12 weeks |

- ‘Solids’ and ‘Hollows’ refers to the particular configuration of the polymer particles that were suspended in the saline carrier medium. (These different particle configurations were included to investigate their effect on the local tissue and cellular responses.)

Methods

The animals were dosed by intramuscular injection with a 21G needle according to the study requirements. The animals were weighed twice a week and monitored for health, pain and distress (behavioural changes) at least daily.

The animals were terminated via Isoflurane overdose.

Observations / analytical procedures

Toxicity was determined by evaluation of reduced food and water intake resulting in weight loss (more than 20% in relative weight loss to the control group), observation of abnormal movement (particularly as it pertains to the ability of the animal to obtain food and water) and ease of breathing.

After termination, organ weights were determined, and histopathology of major organs and all organs showing abnormality was performed at Ampath laboratories in Pretoria. A daily log recording all scheduled and unscheduled events was kept.

Statistical analysis

A quantitative analysis of the histology data was conducted whereby a 0-3 scale was used to rate the implants with respect to the following parameters: fibrosis; necrosis; Granulomatous/foreign body response, acute and chronic inflammation. The rating scale used was as follows: 0 = absent, 1 = mild; 2 = moderate; 3 = severe/marked. Analysis of variance (ANOVA) was used to analyze data.

**Results and discussion**

Inflammation (acute and chronic) was typically the same for both solids and hollows. The inflammation on day 1 is to be expected and is most probably due to the act of injection. It was recorded as being mild. One rat out of the group (n=4) had a mild acute and chronic inflammation reaction at week 4 in the study, but this was statistically insignificant (S1 and S2 Figs).

No fibrosis (scar tissue) was visible with the hollow particles, but fibrosis was present with the solid particles on Day 1 (S3 and S4 Figs). Tissue necrosis was visible in 25% of the test animals with the hollow particles, and not in the solid particles. Both the fibrosis and tissue necrosis were only visible on the first day in statistically insignificant numbers.

**Granulomatous/foreign body response**

The increased foreign body response seen in week 12 could be due to degradation of the PCL resulting in the production of gamma-hydroxycaproic acid. Degradation of the total polymer takes approximately 24 months or more (Middleton and Tipton, 2000). This is due to enzymatic ester hydrolysis in the extra cellular matrix (ECM) (Piskin, 2002). Once enzymes are extracellular they have a chemotactic effect resulting in a foreign body response (Williams, 1992). Degradation data of PCL implanted in the backs of rats for four weeks obtained by Bei et al., (1997) indicated a significant drop in average weight. This resorption is expected and is one of the main reasons for choosing PCL as a polymer in our application. The foreign body response is still mild. The hollows have a higher response than the solids; this could be due to the higher surface area exposed to the surrounding tissue and ECM (S5 Fig).

**Conclusion**

This study indicated that biotoxicity of PCL *in vivo* is low to non-existent as predicted by literature, and where present is reflective of a foreign body response.

**B. Injection of ported PCL particles and MEFs *in vivo***

**Table B: Observations on mice in the *in vivo* experiment assessing the effect of ported PCL particles and cells.**

| **Day** | **Animal number*** | **Comments** |
| --- | --- | --- |
| **2 days prior to injection** | All animals | Weighed and ear clipped |
| **0** | All animals | Injected subcutaneously behind neck  (approximately 0.20 ml) |
| **2** | 1,3,4,8,10,12,13,14,15,16,17,18,19,20,  22,23,24,25,26,27,29,30,31,32,33,34,36 | Scratching behind neck was observed |
| **4** | 8,12,13,14,15,16,17,19,23,24,25,26,27,  29,30,32,33,35,34 | Animals scratched behind neck |
| **5** | 1,3,4,8,9,10,12,14,15,16,17,19,23,24,25, 26,27,29,30,32,33,34 | Wound healing observed |
| **7** | 13 | Scab formed behind neck, induced further scratching |
| **25** | 22,23,24,25,26,27,28,29,30,32,33,34,35,36 | Swollen on dorsal side |
| **26** | 19 | Swollen on dorsal side |
| **59** | All animals | Mice terminated |

* No. 1 - 6: control group receiving only PBS; No. 7 - 16: group receiving ported PCL particles; No. 17 - 26: group receiving MEFs; No. 27 - 36: group receiving ported PCL particles+MEFs

**Table C: Statistical comparisons preformed between the various white blood cell types assessed from blood smears of experimental mice injected with ported PCL particles with or without MEFs.**

| **White blood cell type** | **Test utilized** | **P value*** | **Difference between groups determined, where applicable, with the aid of Tukey-Kramer multiple-comparison test** |
| --- | --- | --- | --- |
| **Monocytes** | One-way ANOVA | 0.00579 | The ported PCL particle group had a significantly lower monocyte count when compared to the control and MEF groups |
| **Lymphocyte** | One-way ANOVA | 0.00353 | The control and MEF groups had a significantly lower lymphocyte count when compared to the ported PCL particle group |
| **Eosinophil** | One-way ANOVA | 0.0719 | No significant difference between any of the groups |
| **Neutrophil** | One-way ANOVA | 0.713 | No significant difference between any of the groups |
| **Basophil** | One-way ANOVA | 0.174 | No significant difference between any of the groups |

* Significance was set at a level of 0.05 or less

**C. Follow up study with polystyrene particles *in vivo***

**Table D: Schedule of the *in vivo* experiment assessing the effect of ported and non-ported PCL as well as polystyrene (PS) particles.**

| **Time** | **Number of mice** | **Group** | **Procedure** | **Date of sacrifice** | **Animal number** |
| --- | --- | --- | --- | --- | --- |
| **0-1 Hour** | 2 | Solid PCL (non- ported) | **09 April 2013 (Inject 56 mice & sacrifice 2)** | 09 April 2013 | # 55,56 |
| **Week 1. A** | 3 | Control (PBS) | Cardiac puncture & histology | 16 April 2013 | # 1,2,3 |
|  | 3 | Solid PCL (non- ported) | C. puncture & histology | 16 April 2013 | # 4,5,6 |
|  | 3 | PCL ported | C. puncture & histology | 16 April 2013 | # 7,8,9 |
|  | 3 | Inert | C. puncture & histology | 16 April 2013 | # 10,11,12 |
| **Week 2. B** | 3 | Control | C. puncture & histology | 23 April 2013 | # 16,17,18 |
|  | 3 | Solid PCL (non- ported) | C. puncture & histology | 23 April 2013 | # 19,20,21 |
|  | 3 | PCL ported | C. puncture & histology | 23 April 2013 | # 22,23,24 |
|  | 3 | Inert | C. puncture & histology | 23 April 2013 | # 25,26,27 |
| **Week 4. C** | 3 | Control | C. puncture & histology | 7 May 2013 | # 28,29,30 |
|  | 3 | Solid PCL (non- ported) | C. puncture & histology | 7 May 2013 | # 31,32,33 |
|  | 3 | PCL ported | C. puncture & histology | 7 May 2013 | # 34,35,36 |
|  | 3 | Inert | C. puncture & histology | 7 May 2013 | # 37,38,39 |
| **Week 8. D** | 3 | Control | C. puncture & histology | 4 June 2013 | # 40,41,42 |
|  | 3 | Solid PCL (non- ported) | C. puncture & histology | 4 June 2013 | # 43,44,45 |
|  | 3 | PCL ported | C. puncture & histology | 4 June 2013 | # 46,47,48 |
|  | 3 | Inert | C. puncture & histology | 4 June 2013 | # 49,50,51 |

Note: although 56 mice were used in the experiment shown here, 6 (#’s 13, 14, 15, 52, 53, 54) were used for assessing another cell delivery product unrelated to the current study. The total number used for the current study was therefore 50.

**Table E: Overview of the animals, tests and procedures performed in the *in vivo* experiment assessing the effect of ported and non-ported PCL as well as polystyrene (PS) particles in BALB/c mice.**

| **Time** | **Number of animals** | **Particles tested** | **Tests performed** | **Notes** |
| --- | --- | --- | --- | --- |
| 0 to 1 hour | 2 | Solid (PCL non-ported) | LM  Whole blood fibrin experiment | Determine whether injected particles can be visualized. Make sure the needle is inserted at 90°. Determine whether the fibrin experiment can be performed on whole blood. |
|  | | | | |
| 1 week | 3 | Control | Fibrin morphology  LM  TEM  PB counts | Blood obtained by cardiac puncture; syringe flushed with citrate. |
|  | 3 | Solid (PCL non-ported) |  |  |
|  | 3 | PCL ported |  |  |
|  | 3 | Inert (PS) |  |  |
|  | | | | |
| 2 weeks | 3 | Control | Fibrin morphology  LM  TEM  PB counts | Blood obtained by cardiac puncture; syringe flushed with citrate. |
|  | 3 | Solid (PCL non-ported) |  |  |
|  | 3 | PCL ported |  |  |
|  | 3 | Inert (PS) |  |  |
|  | | | | |
| 4 weeks | 3 | Control | Fibrin morphology  LM  TEM  PB counts | Blood obtained by cardiac puncture; syringe flushed with citrate. |
|  | 3 | Solid (PCL non-ported) |  |  |
|  | 3 | PCL ported |  |  |
|  | 3 | Inert (PS) |  |  |
|  | | | | |
| 8 weeks | 3 | Control | Fibrin morphology  LM  TEM  PB counts | Blood obtained by cardiac puncture; syringe flushed with citrate. |
|  | 3 | Solid (PCL non-ported) |  |  |
|  | 3 | PCL ported |  |  |
|  | 3 | Inert (PS) |  |  |

**References**

Bei, J-Z., Li, J-M., Wang, Z-F., Le, J-C., Wang, S-G. (1997). Polycaprolactone-Poly(ethylene-glycol) block copolymer. IV: Biodegradation behaviour *in vitro* and *in vivo*. Polymers for Advanced Technologies. 8:693-696.

Middleton, J.C., Tipton, A.J. (2000). Synthetic biodegradable polymers as orthopaedic devices. Biomaterials. 21: 2335-2346.

Piskin, E. Scott, G. (Ed). Chapter 10: Biodegradable polymers in Medicine.p321-378. *Degradable Polymer Principles and Applications*. (2002). Kluver Academic Publishers. Dordrecht, the Netherlands.

Williams, D.F., Cahn, R.W. (Ed.), Haasen, P. (Ed.), Kramer, E.J. (Ed). Chapter 1: Biofunctionality and Biocompatibility. p1-28. *Material Science and Technology: A comprehensive treatment*. WCH Publishers Inc., New York, NY (USA).
