# Supplementary material for: An in vitro and in vivo study on the properties of hollow polycaprolactone cell-delivery particles
Source: PLoS One. 2018 Jul 3;13(7):e0198248. doi: 10.1371/journal.pone.0198248 (PMC6029779; doi:10.1371/journal.pone.0198248)
Supplement: S3 File — (ZIP) [file pone.0198248.s003.zip › In vivo/Experiment 2/Control group.PDF]

UNIVERSITEIT VAN PRETORIA
UNIVERSITY OF PRETORIA
YUNIBESITHI YA PRETORIA

UNIVERSITY OF PRETORIA
Biomedical Research Centre
Onderstepoort

Document ID: All units document
Attachment: Att SOP 1100 (9)

Project Number	H003/12	ANIMAL NOTES FORM	Page No.	1
Animal ID/s	1-56		Group	ALL
			Number animals	56

Date	Description of observations and any treatments administered	Signature
2/4/13	Weigh + mark + Treat with Ivermectin orally	[illegible]
	Topically. 8 Extra mice (one male) M64	
	abscess a base of Tail - Report to Prof	
	Naidoo, sacrifice because of extra mice	
3/4/13	Fighting head in cages - seperate males in these	[illegible]
	cages	
4/4/13	Seperate all males (put in different cages (1 per	[illegible]
	cage.) Weigh all mice. Weight loss # 28 lost	
	0.5g #33 lost 0.3g #52 lost 0.4g	
8.4.2013	Weigh all mice. Nr 5, 19, 33, 31, 46, 52	[illegible]
	56 wounds healing. Nr 5, 26 weightloss.	
8/4/13	Shave all mice + weigh	[illegible]
9/4/13	Anestize mice with Isofo - place a stomach	
	a Inject soul volume of particels as planned	
	after cleaning skin with alchol.	
	Sacrifice # 55+56 while performing C. Puncture	
	to collect blood in Citrate blood tubes	
	Removed tissue of injection site by Prof	
	Pretorius.	
	USE Group forms Mheech →	

AnimalNotes

QA: [illegible] 10 APR 2013

UNIVERSITEIT VAN PRETORIA
UNIVERSITY OF PRETORIA
YUNIBESITHI YA PRETORIA

UNIVERSITY OF PRETORIA
Biomedical Research Centre
Onderstepoort

[redacted]ument ID: All units document
Attachment: Att SOP 1100 (9)

Project Number	H003/12	ANIMAL NOTES FORM	Page No.	1
Animal ID/s	~~1,2,3~~, ~~16,17,18~~, 28, 29, 30, 40, 41, 42	Group	CONTROL (PBS)	
		Number animals	12	

Date	Description of observations and any treatments administered	Signature
9/4/13	Anestitize mice using Isofor - Clean skin on	M
	back with alcohol. Inject PBS S.C and	
	mark site of Injection with Permanent Marker	
10/4/13	Weigh all mice and remark injection site with	M
	permanent marker. Weight loss # 16	
11/4/13	Observe all + remark	M
12/4/13	Weigh + Remark all mice no concerns	M
13.4.2013	Remark all	[illegible]
14.4.2013	Re-mark all	[illegible]
15/4/13	Weigh + remark + Shave mice	M
16/4/13	Remark all mice. Sac # 1, 2, 3. Cardiac Puncture	M
	Collect Blood an Tissue.	
17/4/13	#16 Barbering on the R side 2 sites near the	M
	hips ± 3mm on one site on the L side ±	
	3mm all hair short. Remark all mice	
18/4/13	Weigh + Remark all mice #16 Sites of Barbering	M
	still same as previous day.	
19/4/13	#16 Barbering stopped sites - hair longer	M
	Remark all sites of inoculations on mice	
20/4/13	Remark all mice #16 started Barbering again	M
21/4/13	#16 has now 6 sites of barbering on the	M
	back area of the mouse. Remark all mice	
22/4/13	Weigh + Shave + Remark all mice	M
	Weight loss #16 (still Barbering 6 sites). Weight loss	
	#18, 29	
23/4/13	Remark all mice. Sacrifice # 16, 17, 18	M
	C puncture Collect Blood + Tissue	
	Treat rest with Ivermectin Topically	
24/4/13	Remark all mice - NAD	M

Animal Notes

QA: [signature] [illegible] APR 2013

UNIVERSITEIT VAN PRETORIA
UNIVERSITY OF PRETORIA
YUNIBESITHI YA PRETORIA

UNIVERSITY OF PRETORIA [redacted]ument ID: All units document
Biomedical Research Centre
Onderstepoort
Attachment: Att SOP 1100 (9)

Project Number	H003/12	ANIMAL NOTES FORM		Page No.	2
Animal ID/s	~~28, 29, 30~~, 41, 40, 42	Group	CONTROL		
		Number animals	6 (was 12)		

Date	Description of observations and any treatments administered	Signature
25/4/13	Weigh + Remark all mice. Weightloss #30, 41	[illegible]
26/4/13	Remark all mice	[illegible]
27/4/13	Remark all mice	[illegible]
28.4.13	Remark all mice	[illegible]
29.4.2013	Nr 28 weightloss, clip hair & re-mark all	[illegible]
30/4/13	Remark all mice	[illegible]
1/5/13	Remark all mice - NAD	[illegible]
2/5/13	Weigh + Remark all mice. Weightloss #28, 40, 42	[illegible]
3/5/13	Remark all mice - no concerns	[illegible]
4/5/13	Remark all - no concerns	[illegible]
5/5/13	Remark all - No concerns	[illegible]
06/05/13	Shave + weigh ~~and~~ remark all - NAD	[illegible]
7/5/13	# 28, 29, 30 Sacrificed Using Isoflurane	[illegible]
	C. puncture, collect Blood in Citrate Blood	[illegible]
	Tubes, collect muscle @ injection site	
8/5/13	On instruction of Prof [illegible] Pretorius - no	[illegible]
	need to Remark the mice anymore!	
	No concerns!	
9/05/13	Weigh all mice - #41 lost 0.4g and	[illegible]
	#42 lost 0.1g. All active + alert	
	#40 - Changed cage in the afternoon.	
	Water bottle leaked, bedding wet.	[illegible]
10/5/13	No concerns	[illegible]
13/5/13	Weigh all mice no concerns	[illegible]
14/5/13	NAD	[illegible]
15/5/13	NAD	[illegible]
16/5/13	Weigh all mice #41 have the tip of the tail	[illegible]
	missing - and wound on the tail (suspected the	
	tail was stuck between grid and cage.) lost 1.1g	
	Weightloss # 40, 42	

Project Number	H003/12	ANIMAL NOTES FORM		Page No.	3
Animal ID/s	40, 41, 42	Group	CONTROL		
		Number animals	3		

Date	Description of observations and any treatments administered	Signature
16/05/13	#41: tail tip necrosis with some degloving	
	Rx with iodine for ~days.	
	#41 15:05 Treat with Podine ointment on tail	[illegible]
17/5/13	Weigh #41 and Treat tail with Podine ointment	[illegible]
	seem to be better - dry wounds. picked up	
	weight 0.7g Rest NAD	
18/5/13	Treat #41 Tail with Podine, wounds	[illegible]
	healing well.	
19/5/13	Treat #41 with Podine, healing well	[illegible]
20/05/13	Treat #41 with Podine ointment, wounds	[illegible]
	healing - lost 0.6g weight #40 lost 0.1g	
	inform Marlese	
21/5/13	No treatment, but clean wound present	[illegible]
	, no concerns	
22/5/13	No concerns	[illegible]
23/5/13	Weigh all mice #41 lost weight 0.1 g	
24/5/13	#41 on scab left at tip of Tail - but healing	[illegible]
	well.	
25/05/13	#41 scab still on at tip of the tail, AA Active	[illegible]
	+ alert	
26/05/13	#41 Scab off at tip of tail - healing very	[illegible]
	well.	
27/05/13	Weigh, shave all mice. No 41 biting his	[illegible]
	tail. No weightloss.	
28/5/13	#41 scabs on tail bitten of - wet wounds	[illegible]
	apply Podine on tail - seems very irritated	
	behaviour - Report to Prof Naidoo	
29/5/13	Wounds dry #41 - looking better treat with	[illegible]
	Podine on tail	

Animal Notes

UNIVERSITEIT VAN PRETORIA
UNIVERSITY OF PRETORIA
YUNIBESITHI YA PRETORIA

UNIVERSITY OF PRETORIA
Biomedical Research Centre
Onderstepoort

[redacted]ument ID: All units document
Attachment: Att SOP 1100 (9)

Project Number	H003/12	ANIMAL NOTES FORM	Page No.	3

Animal ID/s	40, 41, 42	Group	Control
		Number animals	3

Date	Description of observations and any treatments administered	Signature
30/5/13	Weigh all mice. weightloss #40, 42. #41	[illegible]
	Tail healing - dry wounds - apply Podine	[illegible]
	on tail	
31/5/13	#41 Wounds healing and smaller - apply Podine	[illegible]
	on tail	
1.6.13	# 41 Mark on tail red, but not wet	[illegible]
2.6.13	# 41 Mark on tail red, but not wet	[illegible]
08.06.13	Weigh and shave all. #41 tail healed. No	[illegible]
	inflammation. No weightloss.	
4/6/13	#41 Tail healing well Sacrifice all using	[illegible]
	Isofor. C. Puncture, collect Blood in citrate	
	blood tubes, Collect Tissue @ injection Site	

Animal Notes
