# Supplementary material for: An in vitro and in vivo study on the properties of hollow polycaprolactone cell-delivery particles
Source: PLoS One. 2018 Jul 3;13(7):e0198248. doi: 10.1371/journal.pone.0198248 (PMC6029779; doi:10.1371/journal.pone.0198248)
Supplement: S3 File — (ZIP) [file pone.0198248.s003.zip › In vivo/Experiment 2/INERT.PDF]

UNIVERSITEIT VAN PRETORIA
UNIVERSITY OF PRETORIA
YUNIBESITHI YA PRETORIA

UNIVERSITY OF PRETORIA
Biomedical Research Centre
Onderstepoort

Document ID: All units document
Attachment: Att SOP 1100 (9)

Project Number	H003/12	ANIMAL NOTES FORM	Page No. 1
Animal ID/s	~~10~~, ~~11~~, ~~12~~, 25, 26, 27, 37, 38, 39, 49, 50, 51	Group	Inert
		Number animals	~~12~~ (9) 6

Date	Description of observations and any treatments administered	Signature
9/4/13	Anestize all mice using Isofor. Clean skin with	[illegible]
	Alcohol. Inject 50ul sc per animal. Mark	
	site of Injection with a permanent Marker	
	Vial was marked with a (I)	
10/4/13	Weigh all mice and Remark site of Injection with	[illegible]
	permanent marker. Weightloss #12, 26, 38	
11/4/13	Observe + Remark all mice	[illegible]
12/4/13	Weigh + Remark all mice. Weightloss #27	[illegible]
13.4.2013	Re-mark all	[illegible]
14.4.2013	Re-mark all	[illegible]
15/4/13	Weigh + remark + Shave all mice	[illegible]
16/4/13	Remark all mice. Sacrifice #10, 11, 12. Cardiac	[illegible]
	Puncture. Collect Blood + Tissue	
17/4/13	Remark all mice.	
18/4/13	Weigh + Remark all mice #27 Weightloss 0.2g	[illegible]
	#38 Weightloss 0.1g	
19/4/13	Remark all mice	[illegible]
20/4/13	Remark all mice #37 very aggressive! trying	[illegible]
	to bite even after closing the cage Bencre!	
21/4/13	Remark all mice #37 very aggesive again	[illegible]
22/4/13	Weigh + Shave + Remark all mice. Weightloss #	[illegible]
	25, 26, 27, 49, 50	
23/4/13	Remark all mice. Sacrifice #25, 26, 27	[illegible]
	C. Puncture + Collect Blood. Treat Rest	
	with Ivermectin Topically	
24/4/13	Remark all mice #37 Very aggressive!	[illegible]
25/4/13	Weigh + Remark all mice - no concerns	[illegible]

Animal Notes

QA: [illegible] 10 APR 2013

UNIVERSITEIT VAN PRETORIA
UNIVERSITY OF PRETORIA
YUNIBESITHI YA PRETORIA

UNIVERSITY OF PRETORIA
Biomedical Research Centre
Onderstepoort

[redacted]ment ID: All units document
Attachment: Att SOP 1100 (9)

Project Number	H003/12	ANIMAL NOTES FORM	Page No.	2
Animal ID/s	~~25, 26, 27~~ (SAC 23/4/13) ~~37, 38, 39~~, 49, 50, 51	Group	INGT	
		Number animals	6 (was 12)	

Date	Description of observations and any treatments administered	Signature
26/4/13	Remark all mice	[signature]
27/4/13	Remark all mice	[signature]
28·4·13	Remark all mice	[signature]
29.4.2013	Nr 49 weightloss. Clip hair & re-mark all	[signature]
30/4/13	Remark all mice	[signature]
1/5/13	Remark all Mice - NAD #37 very aggresive	[signature]
2/5/13	Weigh + Remark all Mice, NAD	[signature]
3/5/13	Remark all mice - no concerns	[signature]
4/5/13	Remark all - no concerns	[signature]
5/5/13	Remark all - no concerns	[signature]
06/5/13	Weigh, shave and re-mark all - NAD	[signature]
7/5/13	Sacrificed # 37, 38, 39. Using Isofor. C Puncture	[signature]
	Collect Blood in Citrate Blood tubes. Harvest	
	Muscle @ Injection site	
8/5/13	On instruction by Prof Resiai Pretorius	[signature]
	no need to Remark the mice no concerns	
9/05/13	weigh all mice → #51 lost 0.6g.	[signature]
10/5/13	No concerns	[signature]
13/5/13	Weigh all mice - NAD	[signature]
14/5/13	NAD	[signature]
15/5/13	NAD	[signature]
16/5/13	Weigh all mice. Weightloss #50, 51	[signature]
17/5/13	NAD	[signature]
18/5/13	NAD	[signature]
19/5/13	NAD	[signature]
20/05/13	weigh and shave all mice - No weightloss	[signature]
21/5/13	NAD	[signature]
22/5/13	NAD	[signature]
23/5/13	Weigh all mice - no concerns	[signature]
24/5/13	NAD	[signature]

Animal Notes

UNIVERSITEIT VAN PRETORIA
UNIVERSITY OF PRETORIA
YUNIBESITHI YA PRETORIA

UNIVERSITY OF PRETORIA
Biomedical Research Centre
Onderstepoort

[redacted]ument ID: All units document
[redacted]ttachment: Att SOP 1100 (9)

Project Number	H003/12	ANIMAL NOTES FORM	Page No.	3
Animal ID/s	49, 50, 51		Group	INCLIT
			Number animals	3

Date	Description of observations and any treatments administered	Signature
25/05/13	NAD	
26/05/13	NAD	
27/05/13	weigh and shave all. #49 lost 0.2g	
28/5/13	NAD	
29/5/13	NAD	
30/5/13	Weig all mice, weigloss #51	
31/5/13	NAD	
1.6.13	NAD	
2.6.13	NAD	
03.06.13	weigh and shave all. NO weightloss	
4/6/13	Sacrifice all using Isofor C Puncture, collect	
	Blood in citrate Blood tubes. Harvest Tissue	
	at injection site	

Animal Notes

UNIVERSITEIT VAN PRETORIA
UNIVERSITY OF PRETORIA
YUNIBESITHI YA PRETORIA

UNIVERSITY OF PRETORIA
Biomedical Research Centre
Onderstepoort

[redacted]ment ID: All units document
[redacted]ttachment: Att SOP 1100 (9)

Project Number	H003/12	ANIMAL NOTES FORM	Page No.	3
Animal ID/s	49, 50, 51		Group	INGIT
			Number animals	3

Date	Description of observations and any treatments administered	Signature
25/05/13	NAD	
26/05/13	NAD	
27/05/13	weigh and shave all. #49 lost 0.2g	
28/5/13	NAD	
29/5/13	NAD	
30/5/13	Weigh all mice, weightloss #51	
31/5/13	NAD	
1.6.13	NAD	
2.6.13	NAD	
03.06.13	weigh and shave all. No weightloss.	
4/6/13	Sacrifice all using Isofor. C Puncture, collect Blood in citrate Blood tubes. Harvest Tissue at injection site	

Animal Notes
