# Supplementary material for: An in vitro and in vivo study on the properties of hollow polycaprolactone cell-delivery particles
Source: PLoS One. 2018 Jul 3;13(7):e0198248. doi: 10.1371/journal.pone.0198248 (PMC6029779; doi:10.1371/journal.pone.0198248)
Supplement: S3 File — (ZIP) [file pone.0198248.s003.zip › In vivo/Experiment 2/PCL -SOLID.PDF]

UNIVERSITEIT VAN PRETORIA
UNIVERSITY OF PRETORIA
YUNIBESITHI YA PRETORIA

UNIVERSITY OF PRETORIA
Biomedical Research Centre
Onderstepoort

Document ID: All units document
Attachment: Att SOP 1100 (9)

| Project Number | H003/12 | ANIMAL NOTES FORM | Page No. | 1 |
|---|---|---|---|---|
| Animal ID/s | 4,5,6, 14 19, 20, 21, 31, 32, 33 43, 44, 45 (55+56) | Group | PCC - SOLID | |
| | | Number animals | 12 (14) mice | |

| Date | Description of observations and any treatments administered | Signature |
|---|---|---|
| 9/4/13 | anestize all mice with Isofor, Clean skin | [illegible] |
| | with ~~eth~~ Alcohol. Inject 50ul S.C and | |
| | Mark site of Injetion with Permanent maker | |
| | Note: | |
| | PCC - Solid - not suspended: sticking to ~~the~~ | [illegible] |
| | needle and rubber of Syringe - did Report to | |
| | Prof Pretorius | |
| | # 56 + 55 was Anestized and Blood Collecting | |
| | via Cardiac Puncture in Citrate Blood tube | |
| | ~~two~~ ml [illegible]. after overdose tissue at site | |
| | of Injection was Removed and Taken | |
| | By Prof Pretorius | |
| 10/4/13 | Weigh all mice, and Remark injection site | [illegible] |
| | with a permanent marker. Weigloss # 44 | |
| 11/4/13 | Observe + remark | [illegible] |
| 12/4/13 | Weigh + Remark all mice # 20 Weigloss | [illegible] |
| 13.4.2013 | Re-mark all. | [illegible] |
| 14.4.2013 | Re-mark all | [illegible] |
| 15/4/13 | Weigh + Remark + Shave all mice | [illegible] |
| 16/4/13 | Remark all mice. Sacrifice # 4, 5, 6 Cardiac | [illegible] |
| | Puncture. Collect Blood + Tissue | |
| 17/4/13 | Remark all mice | [illegible] |
| 18/4/13 | Weigh + Remark all mice # 31 Weigloss 04g | [illegible] |
| 19/4/13 | Remark all sites on mice | [illegible] |
| 20/4/13 | Remark all mice | [illegible] |
| 21/4/13 | Remark all mice | [illegible] |
| 22/4/13 | Weigh + Shave + Remark all mice. Weigloss # | [illegible] |
| | 19, 45 | |
| 23/4/13 | Remark all mice. Sacrifice # 19, 20, 21. C Puncture | [illegible] |
| | + Collect Blood + Tissue. Treat Rest with | |

QA: [illegible] 10 APR 2013

Animal Notes

UNIVERSITEIT VAN PRETORIA
UNIVERSITY OF PRETORIA
YUNIBESITHI YA PRETORIA

UNIVERSITY OF PRETORIA
Biomedical Research Centre
Onderstepoort

Document ID: All units document
Attachment: Att SOP 1100 (9)

| Project Number | H003/12 | ANIMAL NOTES FORM | Page No. | 2 |
|---|---|---|---|---|
| Animal ID/s | ~~31, 32, 33~~, 43, 44, 45 (Sac ~~4, 5, 6, 19, 20, 21~~) | Group | PCC-SOLID | |
| | | Number animals | 6 (was 12) | |

| Date | Description of observations and any treatments administered | Signature |
|---|---|---|
| 23/4/13 | Ivermectin Topically | |
| 24/4/13 | Remark all mice - NAD | |
| 25/4/13 | Weigh + Remark all mice. Weightloss #33, 43 | |
| 26/4/13 | Remark all mice | |
| 27/4/13 | Remark all mice | |
| 28.4.13 | Remark all mice | |
| 29.4.2013 | Nr 43, 44, 45 weightloss. Clip hair and re- | |
| | mark all | |
| 30/4/13 | Remark all mice | |
| 1/5/13 | Remark all mice - NAD | |
| 2/5/13 | Weigh + Remark all mice. Weightloss #33, 44 | |
| 3/5/13 | Remark all mice no concerns | |
| 4/5/13 | Remark all - no concerns | |
| 5/5/13 | Remark all - no concerns | |
| 06/05/13 | Shave + weigh and remark all - #43 lost 0.5g. | |
| 7/5/13 | Sacrificed # 31, 32, 33 Using Isofor. C. Puncture | |
| | Collect Blood in Citrate Blood tubes. Harvest | |
| | Muscle at Injection Site | |
| 8/5/13 | On Instruction By Prof Rosa Pretorius no | |
| | need to Remark mice - no concerns | |
| 9/05/13 | weigh all mice - #43 lost 0.6g - | |
| 10/5/13 | No concerns | |
| 13/5/13 | Weigh all mice - NAD | |
| 14/5/13 | NAD | |
| 15/5/13 | NAD | |
| 16/5/13 | Weigh all mice, weightloss #43 | |
| 17/5/13 | NAD | |
| 18/5/13 | NAD | |
| 19/5/13 | NAD | |
| 20/05/13 | weigh and shave all mice - no weightloss. | |

Animal Notes

UNIVERSITEIT VAN PRETORIA
UNIVERSITY OF PRETORIA
YUNIBESITHI YA PRETORIA

UNIVERSITY OF PRETORIA
Biomedical Research Centre
Onderstepoort

Document ID: All units document
Attachment: Att SOP 1100 (9)

| Project Number | H003/12 | ANIMAL NOTES FORM | Page No. | 3 |
|---|---|---|---|---|
| Animal ID/s | 43, 44 45 | Group | – PCC – Saun | |
| | | Number animals | 3 | |

| Date | Description of observations and any treatments administered | Signature |
|---|---|---|
| 21/5/13 | NAD | [illegible] |
| 22/5/13 | NAD | [illegible] |
| 23/5/13 | Weigh all mice no concerns | [illegible] |
| 24/5/13 | NAD | [illegible] |
| 25/05/13 | NAD | [illegible] |
| 26/05/13 | NAD | [illegible] |
| 27/05/13 | Weigh and shave [illegible] – #45 lost 0.8g. | [illegible] |
| 28/5/13 | NAD | [illegible] |
| 29/5/13 | NAD | [illegible] |
| 30/5/13 | Weigh all mice, weights #44 All these | [illegible] |
| | mice – very active and movements, in | |
| | cage not normal according / compared | |
| | to other cages. | |
| 31/5/13 | #43, 44 abnormal repeated – swinging | [illegible] |
| | behavior observed again | |
| 1.6.13 | #44 chewing on metal bars of cage. | [illegible] |
| 2.6.13 | NAD | [illegible] |
| 03.06.13 | Weigh and shave [illegible] – NO [illegible]. | [illegible] |
| 4/6/13 | Sacrifice all using Isofo. C. Puncture, collects | |
| | blood in Citrate Blood tubes, Collect tissue at | |
| | Injection Site Seeing swinging movements again! | |
| | | |
| | | |
| | | |
| | | |
| | | |
| | | |
| | | |
| | | |
| | | |
| | | |

Animal Notes
