# Supplementary material for: An in vitro and in vivo study on the properties of hollow polycaprolactone cell-delivery particles
Source: PLoS One. 2018 Jul 3;13(7):e0198248. doi: 10.1371/journal.pone.0198248 (PMC6029779; doi:10.1371/journal.pone.0198248)
Supplement: S3 File — (ZIP) [file pone.0198248.s003.zip › In vivo/Experiment 2/PCL- PORTED.PDF]

UNIVERSITEIT VAN PRETORIA
UNIVERSITY OF PRETORIA
YUNIBESITHI YA PRETORIA

UNIVERSITY OF PRETORIA
Biomedical Research Centre
Onderstepoort

Document ID: All units document
Attachment: Att SOP 1100 (9)

Project Number	H003/12	ANIMAL NOTES FORM	Page No.	1
Animal ID/s	~~7, 8, 9~~, 22, 23, 24, 34, 35, 36, 46, 47, 48	Group	PCC - Ported	
		Number animals	12	

Date	Description of observations and any treatments administered	Signature
9/4/13	Anestize all mice with Isofor. Clean skin with	ʌ
	Alcohol. Inject 50ul of sample, and mark	
	Injection site with permanent marker	
	Sample was marked with a (P) on top of	
	vial	
	Note:	
	PCC - Ported was not suspended, partikels sticky	
	to the needle	
MEG ~~9~~ 10/4/13	Weigh all mice, and remark injection site with	ʌ
	a permanent marker. Weightloss #22, 34, 35	
11/4/13	Observe + Remark all	ʌ
12/4/13	Weigh + Remark all mice. Weightloss #22. #46	ʌ
	has a abscess on tail, ± 2mm in diameter	ʌ.
	and is yellow. Did Report to Prof Naidoo	
	Did inform Ilse to treat with Saline and	
	Bactroban if the absess does burst open	
	over the weekend.	
13.4.2013	Re-mark all. Nr 46 abscess still intact.	[illegible]
14.4.2013	Re-mark all. Nr 46 abscess still intact	[illegible]
15/4/13	Weigh + remark + Shave all mice. #46 Abscess	ʌ
	still intact but looks a lot more white and watery	
	Inject 26 G needle and aspirate. Flush with	
	Saline Weightloss 0.5g! Yellow puss coming out	
	Tail from abscess to tipp of tail turned blue	
	MEG ~~to~~ from artery than was MEG punctured.	
	14.32 Check mouse # 46 Tip pink from tip	ʌ
	to abscess - heamatone present at site of abscess	
	Mouse doing well. Apply Bactroban on site	
16/4/13	Remark all mice. Sacrifice # 7, 8, 9. Cardiac	ʌ
	Puncture Collect Blood + Tissue.	

Animal Notes

QA: [illegible] 10 APR 2013

Project Number	H003/12	ANIMAL NOTES FORM	Page No.	2
Animal ID/s	~~22, 23, 24,~~ 34, 35, 36 46, 47, 48	Group	PCL - PORTED	
		Number animals	12 now (9)	

Date	Description of observations and any treatments administered	Signature
16/4/13	#46 weigh + remark 1g weight gain, tail still swollen and heamatome at site of abscess but no puss present. Apply Bactoban 2x dayly	[illegible]
17/4/13	Remark all mice #46 seems to be healing still a bit swollen. Apply Bactroban 2x dayly	[illegible]
18/4/13	Weigh + Remark all mice #46 picked up weight did look at site if it might be more water or puss inside. Did open it up but nothing came out. Apply Bactroban 2x per day. #23 Weightloss 0.2g #34 weightloss 0.2g #35 weight loss 0.2g #48 weightloss 0.2g	[illegible]
19/4/13	Remark all sites on mice #46 Tail seems to be healing nicely, still a bit swollen	[illegible]
20/4/13	Remark all mice #46 part of tail still swollen	[illegible]
21/4/13	Remark all mice #46 Healing well at site of abscess still a bit swollen.	
22/4/13	Weigh + Shave + Remark all mice. Weightloss #23 (0.5g) #46 weightloss (0.2g) On one side of tail still a bit swollen.	[illegible]
23/4/13	Remark all mice. Sacrifice #22, 23, 24. C. Puncture Collect Blood + Tissue. Treat Rest with Ivernebi Topically #46 Tail healing well.	[illegible]
24/4/13	Remark all mice - tail a bump of 2mm on one side #46 - no concerns	
25/4/13	Weigh + Remark all mice. #46 no concerns one side still a bump of ± 2mm (Prof Naidoo checked)	[illegible]
26/4/13	Remark all mice	[illegible]
27/4/13	Remark all mice	[illegible]

Project Number	H003/12	ANIMAL NOTES FORM	Page No.	3
Animal ID/s	~~34, 35, 36~~ 46, 47, 48	Group	PCL - Perfeq	
		Number animals	6 (was 12)	

Date	Description of observations and any treatments administered	Signature
28.4.13	Remark all mice	[signature]
29.4.2013	Nr 34 weightloss, Clip hair and re-mark all	[signature]
30/4/13	Remark all mice	[signature]
1/5/13	Remark all mice - NAD	[signature]
2/5/13	Weigh + Remark all mice, weightloss #47	[signature]
3/5/13	Remark all mice - no concerns	[signature]
4/5/13	Remark all - no concerns	[signature]
5/5/13	Remark all - no concerns	[signature]
06/05/13	Weigh, Shave and re-mark - #48 lost 0.1g	[signature]
7/5/13	Sacrificed # 34, 35, 36 using Isofl. C. puncture, collect Blood in Citrate tubes Harvest muscle @ Injection site	[signature]
8/5/13	On instruction by Prof Vesia Pretorius no need to Remark mice - no concerns	[signature]
9/05/13	weigh all mice - no weightloss.	
10/5/13	No concerns	[signature]
13/5/13	Weigh all mice NAD	[signature]
14/5/13	NAD	[signature]
15/5/13	NAD	[signature]
16/5/13	Weigh all mice NAD	[signature]
17/5/13	NAD	[signature]
18/5/13	NAD	[signature]
19/5/13	NAD	[signature]
20/5/13	weigh and shave all mice - no weightloss	[signature]
21/5/13	NAD	[signature]
22/5/13	NAD	[signature]
23/5/13	Weigh all mice - no concerns	[signature]
24/5/13	NAD	[signature]
25/05/13	NAD	[signature]
26/05/13	NAD	[signature]

Archives

UNIVERSITEIT VAN PRETORIA
UNIVERSITY OF PRETORIA
YUNIBESITHI YA PRETORIA

UNIVERSITY OF PRETORIA
Biomedical Research Centre
Onderstepoort

Document ID: All units document
Attachment: Att SOP 1100 (9)

Project Number	H003/12	ANIMAL NOTES FORM		Page No.	4
Animal ID/s	46, 47, 48	Group		PCL-Pated	
		Number animals		3	

Date	Description of observations and any treatments administered	Signature
27/05/13	Weigh and shave all. #48 lost 0.2g !	[signature]
28/5/13	NAD	[signature]
29/5/13	NAD	[signature]
30/5/13	Weigh all mice - ~~NAD~~ MC4 #46, 47 weigloss	[signature]
31/5/13	NAD	[signature]
1.6.13	NAD	[signature]
2.6.13	NAD	[signature]
03,06,13	weigh and shave all - #46 lost 0.6g.	[signature]
4/6/13	At sacrifice #48 notice abscess on jaw	
	cut open. ± 2-3mm absess present. Sacrifice	
	all using Isofor. C Punctive collect blood	
	in citrate tubes. Harvest Tissue @ injection	
	site	

Animal Notes
