# Supplementary material for: An in vitro and in vivo study on the properties of hollow polycaprolactone cell-delivery particles
Source: PLoS One. 2018 Jul 3;13(7):e0198248. doi: 10.1371/journal.pone.0198248 (PMC6029779; doi:10.1371/journal.pone.0198248)
Supplement: S3 File — (ZIP) [file pone.0198248.s003.zip › In Vitro/Reports/Dermapearl Final Report 26 Feb 2011 Second draft.doc]

**Final Report**

Dermapearl cell-delivery particles

In vitro and in vivo studies

Project undertaken in the Faculty of Medicine, University of Pretoria in collaboration with the Council for Scientific and Industrial Research (CSIR)

University of Pretoria

Prof. Michael S. Pepper (Dept. of Immunology)

Prof Resia Pretorius (Dept. of Anatomy)

Prof. Annie Joubert (Dept. of Physiology)

Dr. Marnie Potgieter (Dept. of Immunology)

Mr. Andre Stander (Dept. of Physiology)

Ms. Karlien Kallemyer (Dept. of Immunology)

Ms. Fiona van Vollenstee (Dept. of Immunology)

CSIR

Dr. Sean Moolman

Dr. Lara Kotze

February 2011

**1. INTRODUCTION**

The field of dermal fillers is rapidly evolving with numerous commercially available products currently on the market. These include collagen, hyaluronic acid, calcium hydroxyapatite (CaHa), poly-_L_-lactic acid, and poly-methylmethacrylate (PMMA) microspheres (Bentkover, 2009). Traditionally the materials intended for co-transplantation with cells have been designed to provide the filler with appropriate physical and chemical properties to provide strength, an appropriate surface for cell attachment and favourable degradation rates. However, designs are starting to include biological criteria to enhance adhesion and control morphogens/cytokines by regulating cell signalling (Lutolf and Hubbell, 2005).

Generally there are four major types of fillers; synthetic, xenogeneic, homogeneic and autogeneic (Ell*is et a*l., 2001). Synthetic fillers are those made artificially; these include silicone, PMMA beads and CaHa. Xenograft fillers imply that the donor and recipient are from different species, and these include bovine collagen and hyaluronic acid (HA) derivatives. Homogeneic fillers, when the donor and recipient are from the same species, include agents derived from human cadaveric tissue. Autogeneic materials, when the donor and recipient is the same individual, include autologous tissues such as autologous fat, collagen and dermal fibroblasts. The common objective with all of these fillers is to provide the ideal filler to suit the needs of the individual patient. An ideal dermal filler will be characterized by, amongst other things, its biocompatibility with human tissue, versatility, stability at the implantation site, long-lasting and natural-appearing filler effects, minimal foreign body reactions, ability to escape phagocytosis and non-migration potential (Coleman, 2001, Lemper*le et a*l., 2004).

The CSIR has developed novel ported polycaprolactone particles (Dermapearl^TM^) through a solvent evaporation process that has application in soft tissue bulking but more specifically in minimally invasive dermal filler procedures for wrinkle reduction (Figure 1) (Naid*oo et a*l., 2008). The hollow ported particles are designed to serve both as an anchor point and a “tissue harbour”^[1]^ for cells, which provides significant advantages compared to both the hydrogel (e.g. HA) and the particulate products currently on the market for soft tissue bulking. The size range of the particles allow for injection of the particles through a 26 gauge needle that is appropriate for facial injection. Polycaprolactone is a well known resorbable polymer that has been used extensively in implantable medical devices *e.g.* a subdermal slow release contraceptive. This is due to its favourable resorption profile and biocompatibility (Ch*en et a*l., 2000).

The dermal filler market is currently serviced by products that offer temporary bulking ranging from three to six months as well as longer lasting products that provide bulking up to 24 months (particle based fillers). The particle based products are either permanent or resorbable but tissue regeneration ability has yet to be confirmed. To maintain the filling effect, patients need to go for regular filling sessions based on the longevity of the product. This has cost and discomfort implications for the patient. A longer lasting resorbable tissue regenerating product such as Dermapearl^TM^ would reduce the need for follow up procedures resulting in a cost/discomfort saving for the patient over the long run. The polycaprolactone particles are resorbed over a longer period compared to the typical hydrogel filler (*e.g.* HA). The particles are thus intended to increase the period over which the minimally invasive cosmetic treatment typically lasts, while being non-permanent, with the potential for permanent tissue regeneration.

**Figure 1:** From left to right: Optical image of the Dermapearl^TM^ ported microspheres; SEM (Scanning Electron Microscopy) image of ported microsphere; SEM image of SKUT-1 (human uterine mixed leiomyosarcoma) cells grown on ported microsphere after 72 hours

The size of the USA dermal filler market for 2009 was approximately 1.7 million doses per annum (2010 report on the 2009 statistics for the American Society of Plastic Surgeons). Approximately 30% of these procedures were micro particle based fillers and indications are that this figure will increase as patients realise the benefits of the longer lasting products.

**2. AIM**

The aim of this project was to investigate various aspects of the behaviour Dermapearl^TM^ particles *in vitro* in co-culture with various cell types and *in vivo* as cell delivery vehicles.

The purported benefit of using the hemi-shell particles lies in the proposed cell harbour concept. The combination of internal and external surface areas should allow for higher cell loadings when compared to a solid particle. The macroporosity of the particle should also allow for increased oxygen and nutrient transfer to cells within the cell harbour. One of the major potential benefits of this cell delivery system is the ability to anchor cells in specific locations in tissue.

**3. MATERIALS AND METHODS**

**3.1 In vitro experiments**

3.1.1 Isolation of mouse embryonic fibroblasts (MEF) and adult skin fiboblasts (ASF)

Mouse embryonic fibroblasts (MEF) were isolated from mouse embryonic tissue and were chosen as the cell type to be anchored onto the Dermapearl^TM^ particles. When implanted into syngeneic mice (BALB/c), these cells are not rejected.

Ethical approval for the use of female BALB/c mice was obtained from the Animal Use and Care Committee (AUCC), University of Pretoria. The animals were kept in Type II conventional mouse cages, females in groups of no more than five and males singly in their cages. Females were superovulated at 12 midday on day 0 with 0.2 ml PMSG per female were administered intraperitoneally (IP). Females were given a second IP injection of 0.2 ml HcG per female after 48 hours at 12 midday. They were then placed with a male for mating to occur. Hormones were kept at -20˚C until the day of use. Hormones were thawed at room temperature and kept in the fridge at 4˚C. The following morning females were inspected for a copulation plug and left with the male until embryos were flushed.

Embryos from five pregnant female BALB/c mice (per isolation) were first isolated at 10 to 12 days of age. Low cell viability was noted in these cultures. Fifteen day-old embryos were then used. Cultures from these tissues yielded higher cell numbers with increased viability and proliferation capacity.

In addition to embryonic tissue, skin was harvested from the ears of adult BALB/c mice as a source of adult skin fibroblasts (ASF).

3.1.2 Cell culture

Aseptic techniques were applied, with all work being conducted in a laminar flow cabinet from Labotec (Midrand, SA). All solutions were filter-sterilized (0.22 µm pore size) and all glassware and non-sterile equipment was sterilized by autoclaving (20 min, 120°C, 15 psi).

Animals were anaesthetised using isofor inhalation and terminated by cervical dislocation. Embryos and adult ear skin tissue were immediately isolated and washed several times with culture medium (DMEM, Sigma). If limbs or the head were visible on the embryos, they were removed together with tissue debris. Tissues were then minced finely in slicing medium (DMEM + 2.5% antibiotics). Resultant tissue fragments were transferred into a tube containing 0.1% collagenase/hyaluronidase solution and incubated for three to six hours.

After incubation, the tissue fragments were separated from the digestion solution by low speed centrifugation and resuspended in culture medium (DMEM + 40% FCS + 1% antibiotics). The solution was then transferred into cell culture flasks and incubated for three days before culture medium was added. Cultures were left undisturbed for seven days. Thereafter medium was replaced with DMEM containing 10% FCS and 1% antibiotics every two to three days until 80% confluency was reached. Following confluency, cells were passaged by trypsin digestion, split 1:3, and expanded until enough cells were yielded for test purposes.

For experiments, cells were seeded in 96-well tissue culture plates (10 000 cells per well), into 24-well culture plates (500 000 cells in 1 ml per well) or in autoclaved Kimble glass tubes (500 000 cells per 1 ml). Cells were incubated for 24h to allow for attachment after which medium was removed and cells were exposed to 5 mg/ml of Dermapearl^TM^ particles in DMEM containing 10% FCS and 1% antibiotics.

Dermapearl^TM^ particles were sterilized by washing three times with 70% ethanol and dried under negative pressure. A solution of growth medium and Dermapearl^TM^ particles was prepared before exposing MEFs to the particles. 5 mg/mL of Dermapearl^TM^ particles were mixed with growth medium and left on a rotary shaker for 1h at 37°C.

Cells were harvested by tripsinization as described above and were counted using a haemocytometer as previously described (Freshney, 1995). Non-viable cells were excluded using the Trypan blue staining procedure. Dead cells take up the dye and are consequently stained blue, which are then left uncounted. The number of viable cells per ml was determined as follows:

Cells/ml = Average count of viable cells in the corner squares x dilution factor x 10^4^

3.3.3 Light microscopy

Polarization-optical differential interference contrast (PlasDIC) light microscopy (Carl Zeiss (Pty) Ltd., Johannesburg, South Africa) (Da*nz et a*l., 2004) was employed to monitor cell morphology cells cultured alone or in combination with Dermapearl™ particles. Images were captured using a Zeiss Axiovert-40 microscope (Göttingen, Germany).

3.3.4 Cell growth analysis

Quantification of monolayer cell cultures was spectrophotometrically determined employing crystal violet as a DNA stain. Staining nuclei of cells fixed with crystal violet allows for rapid, accurate and reproducible quantification of cell number in cultures grown in 96-well plates (Gilli*es et a*l., 1986, Kue*ng et a*l., 1989). Dye absorbance was measured spectrophotometrically at 570 nm. Exponentially growing MEF were seeded with Dermapearl™ (5 mg/ml) particles in 96-well tissue culture plates at a cell density of 10 000 cells per well (100 µl/well). After 24h, 48h or 72h the medium was discarded and 100 µl of 1% glutaraldehyde (in PBS) was added to each well and incubated at room temperature for 15 min. Glutaraldehyde was discarded and 100 µl 0.1% crystal violet (in PBS) was added and left at room temperature for 30 min. The crystal violet was discarded and the microtitre plates were immersed under running tap water for 10 min and left overnight to dry. A 200 µl 0.2% Triton X-100 solution was added to solubilise the cells. The cells were then incubated at room temperature for 30 min before 100 µl of the solution was transferred to a clean microtitre plate. The absorbance was read at 570 nm with an EL_x_800 Universal Microplate Reader from Bio-Tek Instruments Inc. (Vermont, United States of America).

3.3.5 Fluorescence microscopy

A triple fluorescent dye staining method was developed utilizing acridine orange (green), Hoechst 33342 (blue) and propidium iodide (red) fluorescent dyes. Acridine orange is a lysosomotropic fluorescent compound that serves as a tracer for acidic vesicular organelles including autophagic vacuoles and lysosomes (Klions*ky et a*l., 2007). Cells undergoing autophagy will have an increased tendency for acridine orange staining when compared to viable cells, however, acridine orange is not a specific marker for autophagy and therefore other techniques are needed to verify the appearance of increased autophagic activity. Hoechst 33342 is a fluorescent dye that can penetrate intact cell membranes of viable cells and cells undergoing apoptosis, and stains the nucleus. Propidium iodide is a fluorescent dye that is unable to penetrate an intact membrane and therefore stains the nucleus of cells that have lost their plasma membrane integrity due to oncotic and/or necrotic processes. Exponentially growing MEF were seeded with, as well as without Dermapearl™ (5 mg/ml) particles in Kimble glass tubes at a density of 1 000 000 cells per 1 ml (final volume of 1 ml). Cells were incubated at 37°C in 5% CO_2_ for 24h.

After the exposure time, 0.17 ml of Hoechst 33342 solution (3.5 µg/mL in PBS) was added to the medium to provide a final concentration of 0.9 µM and incubated for 30 min at 37°C in a CO_2_ incubator. After 25 min, 0.17 ml of acridine orange solution (4 µg/ml) and 0.17 ml of propidium iodide solution (40 µg/ml in PBS) were added to the medium to give a final concentration of 1 µg/ml and 12 µM respectively and incubated for 5 min at 37°C. After 30 min, the medium was removed and the cells were carefully rinsed three times with PBS before being immersed in clean PBS (1 ml). Cells were examined with a Zeiss inverted Axiovert CFL40 microscope and Zeiss Axiovert MRm monochrome camera under a Zeiss Filter 2 (for Hoechst 33342 (blue)-stained), a Zeiss Filter 9 (for acridine orange (green)-stained) and a Zeiss Filter 15 (for propidium iodide (red)-stained cells). In order to prevent fluorescent dye quenching, all procedures were performed in a dark room (Carl Zeiss (Pty) Ltd., Johannesburg, South Africa).

3.3.6 Scanning electron microscopy

Scanning electron microscopy (SEM) was conducted to determine surface features of the exposed and control cells. SEM is a technique capable of producing high resolution images of a sample surface. The sample to be viewed is coated by a thin layer of an electron dense substance (*e.g.* gold). Electrons are emitted from a tungsten cathode and are accelerated towards an anode. After emission, low energy (<50 eV) secondary electrons originate within a few nanometers from the surface and are detected by a scintillator-photomultiplier device. The resulting signal is rendered into a two-dimensional intensity distribution that can be viewed and saved as a digital image (Hayes and Pease, 1968).

Exponentially growing MEF were seeded with and without Dermapearl™ (5 mg/ml) particles in Kimble glass tubes at a density of 1 000 000 cells per 1 ml (final volume of 1 ml). Cells were incubated at 37°C, 5% CO_2_ for 24h. After 24h incubation, cells were fixed in 2.5% glutaraldehyde in 0.075 M phosphate buffer (pH 7.4 - 7.6) for 1h and rinsed thrice for 5 min each with 0.075 M phosphate buffer. Thereafter cells were fixed in 0.25% aqueous osmium tetroxide for 30 min and rinsed three times in distilled water in a fume cupboard. The samples were dehydrated with increasing concentrations of ethanol (30%, 50%, 70%, 90% & 3 x 100%). Samples were dried utilizing critical point drying whereby the samples were mounted in a chamber. Liquid carbon dioxide (CO_2_) was incorporated into the chamber until it was filled. Ethanol was expelled from the chamber by opening a valve and thereby releasing CO_2_-dissolved ethanol. The valve was closed and the sample was left in liquid CO_2_ for an hour. The vessel was then warmed to 34°C to reach a gaseous stage of CO_2_. Pressure was released slowly; the sample remained in its natural shape and was completely dry. Dried coverslips were mounted on a stub and sprayed with a thin layer of gold. Samples were viewed with a Cryo-SEM (JEOL 840 with Cryostage).

3.3.7 Determination of cell viability

Quantification of plasma membrane damage provides an indication of cell viability (All*en et a*l., 1994). Lactate dehydrogenase (LDH) is present in all cell types and is rapidly released into the cell culture medium upon damage of the plasma membrane (All*en et a*l., 1994) An LDH colorimetric assay was utilized to measure the ability of extracellular LDH to oxidize lactate to reduce nicotinamide adenine dinucleotide (NAD) to reduced nicotinamide adenine dinucleotide (NADH). The LDH cytotoxicity assay kit utilizes 2-(2-methoxy-4-nitrophenyl)-3-(4-nitrophenyl)-5-(2,4-disulfophenyl)-2H-tetrazolium (WST-8) to react with NADH produced by lactate from LDH. The intensity of the generated colour correlates directly with the amount of released LDH as a result of a damaged plasma membrane.

Exponentially growing MEF were seeded with and without Dermapearl™ (5 mg/ml) in 24-well tissue culture plates, as well as Kimble glass tubes at a density of 1 000 000 cells per 1 ml (final volume of 1 ml). The cells were incubated at 37°C, 5% CO_2_ for 24h. Background-controls were included by adding only growth medium with DermaPearl™ (5 mg/mL) particles. A positive control for LDH production was included by adding 10 µl of lysis buffer 30 min before the end of the 24h incubation time. After the 24h incubation period, 10 µl of each sample was transferred to a 96-well plate and incubated for 30 min at room temperature with the 2-(2-methoxy-4-nitrophenyl)-3-(4-nitrophenyl)-5-(2,4-disulfophenyl)-2H-tetrazolium (WST-8 reagent) as indicated by the supplier’s manual. The absorbance was read at 450 nm with 630 nm as reference with an EL_x_800 Universal Microplate Reader from Bio-Tek Instruments Inc. (Vermont, USA). Cytotoxicity was calculated as follows: Cytotoxicity (%) = (TS – BC) / (PC – BC) x 100

(TS = test sample; BC = background control ; PC = positive control ).

3.3.8 Cell cycle analysis

Flow cytometry was employed to analyze the influence of Dermapearl™ (5 mg/mL) particles on cell cycle progression of MEF. Analysis was conducted by ethanol fixation and propidium iodide staining of cells. Propidium iodide was used to stain the nucleus in order to determine the amount of DNA present. The amount of DNA present correlates with the stages of the cell cycle during cell division. Exponentially growing MEF were seeded with, as well as without Dermapearl™ (5 mg/ml) particles in Kimble glass tubes at a density of 1 000 000 cells per 1 ml (final volume of 1 ml). Cells were incubated at 37°C, 5% CO_2_ for 24h. After 24h of incubation, cells were trypsinized and resuspended in 1ml growth medium. Cells (1x10^6^/ml) were centrifuged for 5 min at 300 x *g*. Supernatant was discarded and cells were resuspended in 200 µl of ice-cold PBS containing 0.1% FCS. Ice-cold 70% ethanol (4 ml) was added in a dropwise manner on a vortex to avoid cell clumping. Cells were stored at 4ºC for 24h and subsequently centrifuged at 300 x *g* for 5 min. Supernatant was removed and cells were resuspended in 1 ml of PBS containing 40 µg/ml propidium iodide and 100 µg/ml RNase A. The solution was incubated at 37ºC, 5% CO_2_ for 45 min. Propidium iodide fluorescence (FL3) was measured using the Beckman Coulter South Africa (Pty) Ltd flow cytometer. Data from at least 10 000 cells were analyzed with with Cyflogic (CyFlo Ltd, Turku, Finland).

3.3.9 Logistics

Experiments were conducted in the Department of Physiology of University of Pretoria in conjunction with the Faculty of Veterinary Science of the University of Pretoria. Electron microscopy was conducted at the Electron Microscopy Unit of the University of Pretoria. Flow cytometry analysis was conducted at the department of Pharmacology of the University of Pretoria.

3.3.10 Statistical analysis

Data obtained from three independent experiments (each conducted in six replicates) are shown as the mean ±SD and were statistically analyzed for significance using the analysis of variance (ANOVA)-single factor model followed by a two-tailed Student’s *t*-test. Means are presented in bar charts, with T-bars referring to standard deviations. *P*-values < 0.05 were regarded as statistically significant. Cell number determination, lactate dehydrogenase cytotoxic assay, cell cycle progression analysis were analysed quantitatively, while PlasDIC and transmission electron microscopy were analysed qualitatively.

**3.2 In vivo experiments**

3.2.1 Injection of particles and/or MEF

BALB/c mice were used in these experiments and divided into the following groups:

| **Sample Type** | **No. of animals** |
| --- | --- |
| Control | 6 |
| Particles | 10 |
| Cells | 10 |
| Particles and cells | 10 |

At day zero, all mice were injected subcutaneously behind the neck with 0.20 ml of PBS only, particle suspension (5 mg/ml in sterile PBS), cell suspension (5 mg/ml in sterile PBS), or particles with cell suspension according to the experimental group. The trial period was 59 days, after which all experimental mice were terminated. The injection area was shaved throughout the trial period.

3.2.2 White blood cell counts

Blood smears from mice for each of the four experimental groups were evaluated microscopically. Each slide was quantified in terms of monocytes, lymphocytes, eosinophils, basophils and neutrophils. Each slide was analyzed until 100 white blood cells had been counted. Statistical analysis was preformed with the aid of NCSS on the acquired data so to determine if any significant statistical difference existed between the four experimental groups in terms of their white blood cell species. Statistical assessment was conducted by means of a one-way ANOVA.

3.3.3 Preparation of fibrin clots

Blood was collected on the day of termination via cardiac puncture. Eleven microlitres of citrate was added for every 100 μl of blood drawn. Blood was then centrifuged at 1250 rpm for 2 minutes to obtain platelet rich plasma (PRP).

Human thrombin (provided by The South African National Blood Service) was used to prepare fibrin clots. The thrombin is 20 U/ml and is made up in biological buffer containing 0.2% human serum albumin. When thrombin is added to PRP, fibrinogen is converted to fibrin and intracellular platelet components *e.g.* transforming growth factor, platelet derived growth factor and fibroblastic growth factor are released into the coagulum.

Ten microlitres of mouse PRP was mixed with 10 μl of human thrombin. The PRP and thrombin mix was immediately transferred with a pipette tip to a 0.2 µm millipore membrane to form the coagulum (fibrin clot) on the membrane. This millipore membrane was placed in a Petri dish on filter paper dampened with PBS to create a humid environment and placed at 37ºC for 10 minutes. A washing process followed where the millipore membranes with the coagula were placed in PBS and magnetically stirred for 1h. This was done to remove any blood proteins trapped within the fibrin network (Pretori*us et a*l., 2006).

3.3.4 Preparation of washed fibrin clot for SEM

Washed fibrin clots were fixed in 2.5% glutaraldehyde in Dulbecco's Phosphate buffered saline (DPBS) buffer with a pH of 7.4 for 1h. Each fibrin clot was rinsed three times in phosphate buffer for 5 minutes before being fixed for 1h with 1% Osmium tetraoxide (OsO_4._) The samples were rinsed three times with DPBS for 5 minutes and dehydrated serially in 30%, 50%, 70%, and 90% and three times with 100% ethanol.

The scanning electron microscopy (SEM) procedures were completed by critical point drying of the material, mounting and coating with carbon and examination of the tissue with a ZEISS ULTRA plus FEG scanning electron microscope.

3.3.5 Tissue for histology

Skin samples (epidermis and dermis) for histological investigation were obtained by using a punch with a diameter of 5 mm. Tissue samples were fixed in 2.5% gluteraldehyde/formaldehyde solution. The tissue was removed from the fixative and rinsed three times in 0.075 M sodium potassium phosphate buffer (pH 7.4) for 15 min before being serially dehydrated in 30%, 50%, 70% and 90% ethanol, followed by three changes of absolute ethanol. The samples were then infiltrated in LR White over three days after which they were polymerized in gelatin capsules at 60°C for 48h. One micrometer sections were made with an ultramicrotome and stained with Toluidine blue. The samples were viewed with a Nikon Optiphod transmitted light microscope (Nikon Instech Co., Kanagawa, Japan).

3.3.6 TEM of skin biopsies

Skin biopsies were placed into fixative of 2.5% formaldehyde and 2.5% glutaraldehyde for at least one hour. Tissue samples were rinsed three times for 15 minutes each with 0.075M sodium phosphate buffer, after which the samples were placed in a 1% osmium tetraoxide (OsO_4_) secondary fixative for one hour. After a second round of rinsing three times for 15 minutes each in 0.075M sodium phosphate buffer, series dehydration was performed in 30%, 50%, 70%, 90% and three times absolute ethanol for a period of 10 minutes each. Once dehydrated, TEM tissue samples were infiltrated with a Quetol Epoxy resin in a 1:1 ratio with 100% ethanol for one hour, then changed and infiltrated in resin exclusively overnight. Samples were finally embedded with Quetol Epoxy resin in rubber moulds and allowed to polymerize in an oven at 60°C for approximately 39 hours for ultramicrotoming. Samples were subsequently cut at a thickness of approximately 70-100nm and placed onto copper grids. These were contrast stained using uranyl acetate and Reynolds lead citrate, rinsing the samples 16 times in triplicate with distilled water and dabbing on filter paper to dry after each staining step. Transmission electron microscopic analysis followed using a JEOL Transmission Electron Microscope (JEM 2100F).

**4. RESULTS**

**4.1 In vitro experiments**

4.1.1 Light microscopy

Primary MEF and ASF in culture are shown in Figures 2 and 3. Cells were used before the 3^rd^ passage (3^rd^ trypsinization step) after which cells was frozen at -80°C (10% FCS, 10% DMSO, 80% DMEM).

**Figure 2:** Mouse embryonic fibroblasts (A; 100x, B; 400x) 3 days after harvesting. Cells have a spindle-shaped appearance which is characteristic of normal fibroblasts.

**Figure 3:** Adult skin fibroblasts (A; 100x, B; 400x) 3 days after harvesting. Cells have a spindle-shaped appearance which is characteristic of normal fibroblasts.

4.1.2 Cell growth analysis

Growth rates of MEF and ASF were compared (Figure 4). No statistically significant differences were observed.

**Figure 4:** ASF (mouse ear fibroblasts) (dark grey) cell numbers are expressed as a percentage of cells relative to MEF (light grey - 100%) 24h after seeding. No statistically significant differences in cell growth were observed.

4.1.3 Cell viability

Fluorescence microscopy was employed to differentiate between viable, apoptotic, autophagic and oncotic cells. A triple fluorescent dye staining method was used composing of acridine orange (green), Hoechst 33345 (blue) and propidium iodide (red) fluorescent dyes. Acridine orange is a lysosomotropic fluorescent compound that serves as a tracer for acidic vesicular organelles including autophagic vacuoles and lysosomes (Klions*ky et a*l., 2007). Cells undergoing autophagy will have an increased tendency for acridine orange staining when compared to viable cells, however, acridine orange is not a specific marker for autophagy and if incubated for longer time intervals, it will react with lysosomes as well. As already mentioned Hoechst 33342 is a fluorescent dye that can penetrate intact cell membranes of viable cells and cells undergoing apoptosis and stains the nucleus. Propidium iodide is a fluorescent dye that is unable to penetrate an intact membrane and therefore stains the nucleus of cells that have lost their membrane’s integrity due to oncotic or necrotic processes. Hoechst staining was not specific for cells as it also stained the particles. Background fluorescence from the particles interfered with the identification of nuclei of cells. A modified protocol was conducted whereby the cells were stained with acridine orange for a prolonged period in order to stain all the lysosomal compartments of cells that were attached to the particles.

Cells that were cultured with Dermapearl™ particles in 24-well plates did not attach to particles in sufficient numbers after 24h to allow for further experiments (Figure 5). Cells cultured in Kimble glass tubes together with the Dermapearl™ particles revealed increased adherence when compared to cells cultured in 24-well plates (Figure 6). Subsequent studies were conducted in Kimble glass tubes. Cells that adhered to the particles did not stain with PI, indicating that the cell membranes of these cells were intact (data not shown).

**Figure 5:** Acridine orange-stained Dermapearl™ particles (A; 100x, B; 200x) of MEF propagated in 24-well plates together with particles 24h after seeding. Few cells attached to the particles.

**Figure 6:** Acridine orange-stained MEF attached to Dermapearl™ particles (200x) of cells propagated in glass Kimble tubes together with particles (A and B) 24h after seeding. MEF attached to particles as clumps.

4.1.4 Scanning electron microscopy

Scanning electron microscopy was performed to determine the surface features of the Dermapearl™ particles and also the MEF that had adhered to the particles. MEF aggregated in small groups between multiple particles (Figure 7A), causing particles to clump together. The surface of the particles is relatively smooth when compared to the small protrusion on the cell membrane of an adherent MEF (Figure 7B).

**Figure 7:** Scanning electron micrographs of MEF attached to Dermapearl™ particles 24h after seeding (A and B). MEF grew between particles causing the particles to clump together (A).

4.1.5 Determination of cell viability

2-(2-Methoxy-4-nitrophenyl)-3-(4-nitrophenyl)-5-(2,4-disulfophenyl)-2H-tetrazolium (WST-8) was used to investigate the possibility of a cytotoxic effect of Dermapearl™ particles on MEF. WST-8 indirectly measures the amount of extracellular LDH as an indication of cell membrane damage by reacting with NADH produced by lactate from LDH. The intensity of the colour formed by the reaction correlates directly with the amount of LDH released and thus provides an indication of the relative extent of plasma membrane damage caused by a cytotoxic agent. The ability of the extracellular supernatant of MEF in growth medium, as well as MEF in growth medium attached to Dermapearl™ particles to reduce WST-8 to red formazan (an indication of cytotoxicity) was expressed as a percentage of the positive control (Figure 8). Cell viability of MEF cultured in DMEM together with the Dermapearl™ particles appears on average to decrease when compared to cells propagated in DMEM only (Figure 8). However, the difference was not statistically significant (Table 2).

**Figure 8:** Cell viability of MEF cultured in cultured in Kimble glass tubes in growth medium (A) and growth medium together with Dermapearl™ particles (B) 24h after seeding. No statistically significant difference was observed in cytotoxicity between cells cultured growth medium and cells cultured in growth medium together with Dermapearl™ (*P*-value > 0.05).

**Table 1:** Measurement of cytotoxicity of MEFs cultured in Kimble glass tubes in growth medium (A) and growth medium together with Dermapearl™ particles (B) 24h after seeding

|  | **% Cytototoxicity of MEF in growth medium** | **% Cytototoxicity of MEF in growth medium with Dermapearl™ particles** | ***P*-value**  **(ANOVA)** |
| --- | --- | --- | --- |
| **Kimble glass tube** | 3.34 | 11.81 | 0.106201* |

*No statistically significant difference was observed in cytotoxicity between cells cultured growth medium and cells cultured in growth medium together with Dermapearl™ particles (*P*-value > 0.05).

# 4.1.6 Cell cycle analysis

DNA content of cells was measured as an indication of cells in the various stages of the cell cycle in order to determine the effect of the Dermapearl™ particles on cell cycle progression (Figure 9). No statistically significant differences in cell division and mitotic activity were observed (Table 2).

**Figure 9:** Cell cycle histograms (FL3 Lin) of MEF cultured in Kimble glass tubes in growth medium (A) and growth medium together with Dermapearl™ particles (B) 24h after seeding. No significant differences were observed.

**Table 2:** Measurement of DNA content of MEF cultured in 24-well plates in growth medium and growth medium together with Dermapearl™ particles, as well as cells cultured in Kimble glass tubes in growth medium and growth medium together with Dermapearl™ particles 24h after seeding as an indication of cells in various stages of the cell cycle

| **Cell cycle phase** | **MEF in growth medium** | **MEF in growth medium with Dermapearl™ particles** | ***P*-value**  **(ANOVA)** |
| --- | --- | --- | --- |
| **Sub-G_1_** | 1.39 | 2.02 | 0.168218 |
| **G_1_** | 79.42 | 80.77 | 0.267861 |
| **S** | 7.80 | 6.46 | 0.091445 |
| **G_2_/M** | 11.39 | 10.74 | 0.268161 |

**4.2 In vivo experiments**

4.2.1 Observation for adverse reactions

Mice were observed during the trial period. More than 50% of mice in each experimental group scratched at the site of injection during day two to four and wound healing occurred around day five. At day 24 and 26 the cell group and particles with cells group presented with some swelling on the dorsal side.

**Table 3:** The observations made for experimental mice during the 59 day trial

| ***Day*** | ***Animal number**** | ***Comments*** |
| --- | --- | --- |
| ***2 days prior to injection*** | All animals | Weighed and ear clipped |
| ***0*** | All animals | Injected subcutaneously behind neck  (approximately 0.20 ml) |
| ***2*** | 1,3,4,8,10,12,13,14,15,16,17,18,19,20,  22,23,24,25,26,27,29,30,31,32,33,34,36 | Scratching behind neck was observed |
| ***4*** | 8,12,13,14,15,16,17,19,23,24,25,26,27,  29,30,32,33,35,34 | Animals scratched behind neck |
| ***5*** | 1,3,4,8,9,10,12,14,15,16,17,19,23,24,25,  26,27,29,30,32,33,34 | Wound healing was observed |
| ***7*** | 13 | Scab formed behind neck, induced further scratching |
| ***25*** | 22,23,24,25,26,27,28,29,30,32,33,34,35,36 | Swollen on dorsal side |
| ***26*** | 19 | Swollen on dorsal side |
| ***59*** | All animals | Mice terminated |

* No. 1 - 6: control group receiving only PBS, No. 7 - 16: group receiving particles, No. 17 - 26: group receiving cells,

No. 27 - 36: group receiving particles with cells

4.2.2 White blood cell counts

The parametric one-way ANOVA test was utilized as all the situations considered met the necessary assumptions of the test. Table four provides the statistical tests run for each white blood cell species and the outcomes thereof.

**Table 4:** The statistical comparisons preformed upon the various white blood cell species derived from the blood smears of the experimental mice

| **White blood cell species** | **Test utilized** | **P value*** | **Difference between the compared groups determined, if applicable, with the aid of Tukey-Kramer Multiple-Comparison Test’s** |
| --- | --- | --- | --- |
| **Monocytes** | One-way ANOVA | 0.00579 | The particle group possessed a significantly lower monocyte count in comparison to both the cell and control groups |
| **Lymphocyte** | One-way ANOVA | 0.00353 | The control and cell group both possessed a significantly lower lymphocyte count in comparison to the particle group |
| **Eosinophil** | One-way ANOVA | 0.0719 | No significant difference existed between any groups eosinophil counts |
| **Neutrophil** | One-way ANOVA | 0.713 | No significant difference existed between any groups neutrophil counts |
| **Basophil** | One-way ANOVA | 0.174 | No significant difference existed between any groups basophil counts |

* Significance was set at a level of 0.05

**Figure 10:** Average lymphocyte counts for each of the experimental groups. Y-error bars represent standard deviation (only 6 slides)

**Figure 10:** Average lymphocyte counts for each of the experimental groups. Y-error bars represent standard deviation (all slides)

**Figure 11:** Average monocyte counts for each of the experimental groups. Y-error bars represent standard deviation (only 6 slides)

**Figure 11:** Average monocyte counts for each of the experimental groups. Y-error bars represent standard deviation (all slides)

**Figure 12:** Average neutrophil counts for each of the experimental groups. Y-error bars represent standard deviation (only 6 slides)

**Figure 12:** Average neutrophil counts for each of the experimental groups. Y-error bars represent standard deviation (all slides)

**Figure 13:** Average basophil counts for each of the experimental groups. Y-error bars represent standard deviation (only 6 slides)

**Figure 13:** Average basophil counts for each of the experimental groups. Y-error bars represent standard deviation (all slides)

**Figure 14:** Average eosinophil counts for each of the experimental groups. Y-error bars represent standard deviation (only 6 slides)

**Figure 14:** Average eosinophil counts for each of the experimental groups. Y-error bars represent standard deviation (all slides)

**Figure 15:** Average white blood cell counts for each of the experimental groups. Y-error bars represent standard deviation; ◼ - control; ◼ - particles; ◼ - cells and ◼ - particles and cells (only 6 slides)

**Figure 15:** Average white blood cell counts for each of the experimental groups. Y-error bars represent standard deviation; ◼ - control; ◼ - particles; ◼ - cells and ◼ - particles and cells (all slides)

4.2.3 SEM analysis of fibrin networks in controls, cells, particles and particles and cells

*Controls*

In controls, typically thick, major fibers (thick white arrows) and thin, minor fibers (thin, white arrows) are seen. In the current study this morphology was present. In previous research (both mouse and animal) controls show this ultrastructure (Pretori*us et a*l., 2007).

**Figure 16:** Fibrin network from control BALB/c animal. Thick, white arrow = major, thick fibers; thin, white arrow = minor, thin fibers. Scale = 200 nm

*Experimental groups*

During previous research, where inflammation was introduced in BALB/c animals, changed fibrin morphology has been noted (Pretori*us et a*l., 2007). During inflammation, the thin, sparingly arranged minor fibrin fibers form a thick net, intertwined with the major, thick fibers. In severe cases, thick plagues form. This is also seen in humans during inflammation.

In the current study, the morphology of all 3 experimental groups is changed, and it seems as if inflammation is present. This is seen in Figures 17 to 19. Severe inflammation is seen in particles/cells group.

*Cells*

**Figure 17:** Fibrin network from BALB/c animal injected subcutaneously with embryo cells. Areas of typical fibrin morphology are present. Thick, white arrow = major, thick fibers intertwined; thin, white arrow = minor, thin fibers. Scale = 200 nm

*Particles*

|  |  |  |
| --- | --- | --- |
| **Figure 18: (a & b)** Fibrin network from BALB/c animal injected subcutaneously with particles. No areas of typical fibrin morphology are present. Thick, white arrow = major, thick fibers forming a matted layer. Scale = 200 nm | | |

*Particles and cells*

|  |  |  |
| --- | --- | --- |
| **Figure 19: (a & b)** Fibrin network from BALB/c animal injected subcutaneously with particles. No areas of typical fibrin morphology are present. Thick, white arrow = major, thick fibers forming a matted layer. Scale = 200 nm | | |

4.2.4 Histology of subcutaneous hypodermis and fatty tissue

*Controls*

In control tissue, large, round fat cells sparingly surrounded by loose connective tissue are seen. This is typical hypodermal and fatty tissue morphology in mice and humans.

| 20a  | 20b  |
| --- | --- |
| 20c |  |
|  |  |
| **Figure 20: (a & b)** Control subcutaneous fatty tissue and loose connective tissue; black arrows = large, round fat cell; white arrow = blood vessel in loose connective tissue. Scale = 40x magnification. **(c)** Control subcutaneous fatty tissue and loose connective tissue; black arrows = large, round fat cell; white arrow = loose connective tissue. Red arrow = fibroblast; Scale = 100x magnification. | |

*Cells*

In the animals injected with MEF, the membranes of the fatty cells seem wavy. (Figure 21). A bit more white blood cell infiltration is visible than in the control micrographs.

| 21a | 21b |
| --- | --- |
|  |  |

**Figure 21: (a & b)** Subcutaneous fatty tissue and loose connective tissue of animals injected with embryonic cells; black arrows = large, round fat cell; white arrow = loose connective tissue. Red arrow = fibroblasts/white blood cells; Scale = 100x magnification

*Particles*

Particles in the size-range of 50-150 nm were found embedded amongst the fatty tissue cells.

| 22a   |  | 22b   |
| --- | --- | --- |
| **Figure 22: (a)** Subcutaneous fatty tissue and loose connective tissue of animals injected with particles; black arrows = periphery of large, round fat cells; two green arrows = mass of particles of different sizes; Scale = 40x magnification. **(b)** Subcutaneous fatty tissue and loose connective tissue of animals injected with particles; two black arrows = periphery of large, round fat cell; white arrow = loose connective tissue. Two green arrows = mass of particles of different sizes; Scale = 100x magnification. | | |

*Particles and cells*

Particles in the size-range of 50-150 µm were found embedded amongst the fatty tissue cells. Substantial infiltration of white blood cells is seen in figure 8.

| 23a |  | 23b   |
| --- | --- | --- |
| **Figure 23: (a)** Subcutaneous fatty tissue and loose connective tissue of animals injected with particles and cells; white arrows = substantial infiltration of white blood cells; green arrows = mass of particles of different sizes; Scale = 40x magnification. **(b)** Subcutaneous fatty tissue and loose connective tissue of animals injected with particles and cells; white arrows = substantial infiltration of white blood cells; green arrows = mass of particles of different sizes; Scale = 100x magnification | | |

4.2.5 TEM of skin biopsies

Particles could be identified in skin biopsies of both the particles and particles/cells groups. The aim of the TEM investigation was to determine if any cells could be detected inside the particles. In both these groups, no cells were present inside the particles. (Figures 24 and 25). These results reflect the conclusion that was made after the study of the light microscopy samples, stating that cells did not migrate to the inside of the particles.

| 24a   |  | 24b   |
| --- | --- | --- |
| **Figure 24** Representative TEM of skin biopsies of *particles* group. | | |

| 25a   |  | 25b   |
| --- | --- | --- |
| **Figure 25** Representative TEM of skin biopsies of *particles/cells* group. | | |

**5. DISCUSSION**

Apart from its tissue filling requirement, the ideal tissue filler needs to cause minimal adverse effects. Thus the most important aspect when dealing with tissue fillers is their safety and biocompatibility. Biocompatibility can be achieved with careful design, concentrating on chemical composition, surface structure, surface charge and particle size (Laeschke, 2004). Dermapearl^TM^ is made from the biodegradable polymer polycaprolactone, which has a favourable resorption profile and is biocompatible. A benefit of Dermapearl^TM^ is its longer lasting resorbable tissue filler effect and its novel hollow ported architecture. These hollow ports have been shown to act as a sheltered environment, protecting cells from stress experienced during *in vivo* transplantation as well as creating an anchor point which is conducive for cell expansion (Steynberg, 2010).

In the series of experiments described herein, the *in vitro* studies were conducted to evaluate the effect of Dermapearl^TM^ particles on co-cultured MEF and ASF. During initial cell culturing, both MEF and ASF demonstrated a healthy spindle-shaped morphology characteristic of normal fibroblasts (Figures 2 and 3). Initial attempts at isolating MEFs from 10 to 12 day embryos were unsuccessful, and it was found that cells from 15 day embryos could be cultured with success. No significant differences in cell numbers/growth rates were observed between MEF and ASF. MEF were chosen for all further experiments. Dermal fibroblasts have been used as autogeneic filler material; however embryonic fibroblasts may have the advantage of being younger, and would therefore be expected to contribute to more efficient regenerative capacity *in vivo*. The issue under investigation is whether Dermapearl^TM^ particles are toxic to cells.

Both fluorescent microscopy and SEM of Dermapearl^TM^ particles co-cultured with cells showed that few cells attach to these particles. However, where attachment did occur, it resulted in clumping of the particles (Figures 5, 6 and 7). Cell viability of MEFs cultured in medium together with particles appeared on average to decrease compared to cells propagated in medium only, however the difference was not significant (Figure 8, Table 2). Also, no significant difference in cell division and mitotic activity was observed in the cell only group and cells with particles group (Figure 9, Table 2).

Although the favourable polymer used in the particle design, the particle size range (between 50 to 150 µm which should prevent migration of particles within tissues), and its smooth surface and regular shape should make this a promising filler in basic design, the lack of MEF attachment to Dermapearl^TM^ particles and the clumping phenomenon may be a limiting factor. Cell and particle aggregation could be attributed to the incubation procedure used. Refinement of this procedure to ultimately obtain a monolayer of cells on the particle surface would be the first step needed to making this procedure more viable. Our *in vitro* findings also highlight the fact that *in vivo* delivery of co-cultured cells may not have achieved the intended purpose i.e. delivery of large numbers of cells from injected particles. It is possible that a larger needle will be necessary for sub-dermal delivery to prevent mechanically induced damage to the cells. It is possible that the use of an alternative cell type, *e.g.,* mesenchymal stem cells, may prevent this clustering effect in co-culture with Dermapearl^TM^. Particle size did vary greatly from too small to too big. A narrower size range should be considered. The literature suggests 80 to 120 µm to be an ideal size for a particle to fit the criteria to be used as a dermal filler (Laeschke, 2004). It is also disconcerting to note that both light and electron (TEM) microscopy of the injected particles revealed the complete absence of the so-called “port” which is required to provide the “tissue harbour” effect. The images in Figure 1 (source?) are not confirmed in any of the images of particles that we obtained *in vivo* (Figs 22-25), although the presence of a port may be visible from experiments conducted *in vitro* (Figs 6&7). Are the particles used *in vitro* and *in vivo* from two different batches? If not, had some transformation occurred *in vivo*?

Cell-material interactions can be modulated. When an inert surface is designed, it does not allow for adsorption of proteins and adhesion of cells. It aims to prevent immune response activation and any interaction between material and the surrounding environment. Such a design is biocompatible; however it applies to more permanent synthetic replacement of damaged tissues. For co-transplantation of cells with cell delivery vehicles, materials promoting cell attachment, migration, proliferation, differentiation, long-term viability and cell functioning are more appropriate. This kind of delivery vehicle entails tissue engineering which aims at creating “hybrid bioartificial organs” (Bacáko*vá et a*l., 2004). This implies a temporary structure (the three-dimensional scaffold) promoting tissue regeneration which can be resorbed by growing cells and gradually replaced by the newly formed extra cellular matrix (ECM) and differentiated cells. From the experimental observations, the mechanism of cell adhesion on Dermapearl^TM^ is most likely non-receptor mediated. This means that a non-specific interaction between cells and material is occurring. These interactions include weak chemical bonding such as hydrogen bonding, electrostatic, polar or ionic interactions occurring between the molecules on the cell membrane and the functional chemical groups on the polymer. A weak bonding interaction could have lead to less successful cell attachment and loss of cells during sample preparation (SEM and Fluorescence microscopy involve various steps). Further questions that should then be raised include, ‘how can the control over cell-material interactions be controlled?’ and, ‘is receptor-mediated binding through ECM molecules an option to consider?’

As no major problems, except for low cell attachment and clumping, were observed in *vitro,* an *in vivo* study was initiated to further test the inflammatory response of mice once subcutaneously injected with the different cell, particle and cell-particle preparations.

There are two types of immune response, the innate response and the adaptive response. The innate response is the first line of defence. It is a rapid immediate response and involves amongst others phagocytosis; the removal and engulfment of invasive microbial pathogens. The adaptive response involves T and B-lymphocyte activation. T-lymphocytes can be activated into CD4^+^ T-helper cells, responsible for the cell mediated immunity, and CD8^+^ T-cytotoxic cells, responsible for cytokine secretion. During an inflammatory response, the initial short term process which appears within minutes is called acute inflammation. It is mediated by granulocytes and at the onset of an infection activates and releases inflammatory mediators. Acute inflammation is recognized by vasodilation; an increase in blood flow causing redness and increasing heat, increased permeability of blood vessels; resulting in accumulation of fluid in tissue causing swelling, and migration and infiltration of leukocytes to site of injury. Chronic inflammation develops when the initial inflammatory response is unable to clear the infection or foreign body. It is mediated by mononuclear cells such as monocytes and lymphocytes (Janew*ay et a*l., 2005).

During the trial period, few visible symptoms of acute inflammation on the skin surface were noticeable. Initial scratching at the site of injection was observed in more than 50% of the mice in each experimental group (Table 3). Even the control injected with sterile PBS scratched, suggesting no correlation between scratching and irritation due to an immune response could be made. In all experimental animals, the hair was removed from the site of injection, by shaving with a clipper. It is likely that this procedure could contribute to the skin irritation observed. However, the hair was continuously shaved off during the trial period, yet scratching did not continue after day four, thus irritation from injection may have been the cause. No pus was visible at the injection sites, but the cell group and particle with cell group presented with some swelling on the dorsal side. It can be suggested that the cells themselves caused some type of reaction, perhaps due to unobserved alterations that could have occurred as a result of the *in vitro* culture procedures.

Discuss white blood cell counts

During blood clotting, thrombin interacts with fibrinogen, whereby thrombin cleaves the fibrinogen molecule into two peptides called fibrinopeptides. Ultimately these peptides form the fibrin monomers. It is the assembly of the fibrinogen monomers which forms the fibrin network that may be studied using ultrastructural techniques (Vine, 2009).

When studying a fibrin network in the laboratory, a fibrin clot must be induced, and this may be done by the external addition of thrombin to plasma, ensuing fibrin network formation. Therefore, when studying the ultrastructure of fibrin networks, the addition of thrombin is necessary to form an expansive, fully coagulated layer of fibrin fibers. In previous research, it has been noted that inflammation may cause a changed fibrin network. These changes are visible using SEM. Typically, the fibrin network changes to form thickened layers, have a netted appearance, or from thickened plates. The murine BALB/c asthma model is one such animal model and it has previously been used successfully for a number of *in vivo* immunological applications and for testing novel therapeutics. Pretorius and co-workers in 2007 were the first to use this model to study fibrin ultrastructure and changes in platelet ultrastructure during asthma (Pretori*us et a*l., 2007).

In the current study, fibrin networks also show a changed morphology. The changes are similar as seen in BALB/c animals where inflammation was induced (Pretori*us et a*l., 2007). During inflammation, the thin, sparingly arranged minor fibrin fibers form a thick net, intertwined with the major, thick fibers. In severe cases, thick plaques formed. This is also seen in humans during an ongoing inflammatory process. In the current study, the fibrin morphology of all three experimental groups changed, from a neat expanse of thick and thin fibers to areas of matting occurring, indicating the presence of inflammation. This is seen in Figures 17 to 19. Severe changes in fibrin ultrastructure are seen in particles/cells group. This might suggest that the injection of foreign particles may have triggered an acute inflammatory state in the animals. The fact that the inflammation is seen when preparing fibrin clots from blood, may suggest a trigger of systemic inflammation.

The particles were found to be located in the hyperdermis and fatty tissue (Figure 22 and 23). The control group showed a typical morphology with large round fat cells (Figure 20). Where only cells were injected, the membranes of the fatty cells became wavy with more white blood cell infiltration visible compared to the control (Figure 21). This wavy appearance may be an artefact resulting from the staining procedures. A problem faced was the identification of the injected fibroblasts. New fibroblasts and resident fibroblasts could not be distinguished. It would have been interesting to determine whether the injected fibroblasts, although autologous, were rejected and removed by the immune system. Further tests using GFP-labelled cells would make the injected cells traceable. A large number of particles were embedded amongst the fatty tissue in both the particle and particle with cells groups. The particle with cells group showed substantial white blood cell infiltration and the cell group had some white blood cell infiltration, while the particle group had no visible white blood cell infiltration. Thus, the cells may have been the cause of the inflammation and not necessarily the particles.

All the experimental information, both *in vitro* and *in vivo*, highlighted some important findings.

Cell-based therapies show great promise therapeutically. Numerous adult cell types have been proposed for the repair of damaged or defective tissues. However, two challenges faced include the ability to deliver these cells to the respective sites of injury, as well as their ability to be retained at the site. The purpose of CSIR was to address these issues with their Dermapearl^TM^ particle design. Their novel hollow ported particles seem to create a sheltered environment for cells, but in reality there were no experimental observations made of cells settling into these ports. In fact very few cells were seen to attach to the external surface to the particles, and the hollow ported system was not visible by light microscopy or TEM *in vivo* (skin biopsies). The results seemed to imply using these particles on their own rather than in a co-cultured combination with cells. A concern with using Dermapearl^TM^ is the risk of inflammation. The extent of inflammation which occurred was difficult to determine without baseline observations throughout the trial run. It did seem as if the particles caused some type of immune response, but whether this was beneficial or detrimental remain for future investigations.

In summary, this first round of *in vitro* and *in vivo* experiments using Dermapearl^TM^ particles has provided the following information:

1. MEF do attach to the particles, although attachment is limited and clumping is observed.
2. Some degree of toxicity from Dermapearl^TM^ particles *in vitro* in co-cultures is observed, although this did not reach statistical significance in our experiments. Perhaps significance would be obtained if more experiments were done.
3. When the cells and/or particles were injected *in vivo*, a systemic inflammatory response was noted. A semi-quantitative estimate suggests the following gradation of severity:

control < cells < particles < cells + particles

**6. RECOMMENDATIONS**

Based on the observations from this first round of *in vitro* and *in vivo* experiments using Dermapearl^TM^ particles and MEF, we would like to recommend the following:

1. The CSIR has developed novel ported polycaprolactone particles (Dermapearl^TM^) through a solvent evaporation process. The following issues need to be addressed with regard to particle synthesis and analysis:
   a. Lack of cell attachment and clumping
   b. Apparent toxicity of the particles *in vitro*
   c. Apparent pro-inflammatory nature of the particles/solvent *in vivo*
   d. Lack of ported morphology and tissue harbour effect
   e. Particle size
2. Cells used in these experiments. We have used autologous MEF in all our experiments for the following reasons:
   a. The need for primary cells (as opposed to cell lines/transformed cells)
   b. The need for “young” cells with a greater proliferative capacity than adult cells
   c. The need for autologous/histocompatible cells to avoid rejection upon introduction into syngeneic mice

In future experiments we may wish to try adult primary cells since MEF alone appeared to induce a pro-inflammatory state.

3. We may wish to alter other parameters in vivo including cell number, kinetics etc. in order to refine our observations.
4. Labelling of cells with GFP (lentivirus-mediated) may assist in tracking the injected cells.

It is clear from this report that the UP team has invested a very significant amount of time and energy into this project. One of the requirements in an academic setting is that we are accountable for the time (and resources) we spend on a project such as this. In its present state, the data can (and possibly should) be published, but this may not be acceptable to the CSIR. Additional experiments would therefore probably be needed to satisfy all parties. However, given the fact that our attempts to raise funding for this project from non-CSIR sources have not been successful, further funding from the CSIR would be required to take this forward.

A series of experiments was proposed in October 2010 to address all of the issues raised above. This is included in Annexe A below.

**7. REFERENCES**

Allen, M., Millett, P., Dawes, E. & Rushton, N. (1994) Lactate dehydrogenase activity as a rapid and sensitive test for the quantification of cell numbers in vitro. *Clin Mater,* 16**,** 189-94.

Bacáková, L., Filová, E., Rypácek, F., Svorcík, V. & Starý, V. (2004) Cell adhesion on artificial materials for tissue engineering. *Physiol Res,* 53 Suppl 1.

Bentkover, S. H. (2009) The Biology of Facial Fillers. *Facial plast Surg,* 25**,** 73-85.

Chen, D. R., Bei, J. Z. & Wang, S. G. (2000) Polycaprolactone microparticles and their biodegradation. *Polymer Degradation and Stability,* 67**,** 455-459.

Coleman, S. R. (2001) Structural fat grafts: the ideal filler? *Clin Plast Surg,* 28**,** 111-9.

Danz, R., Vogelgsang, A. & Käthner, R. (2004) PlasDIC - a useful modification of the differential interference contrast according to Smith/Nomarski in transmitted light arrangement.

Ellis, D. A., Makdessian, A. S. & Brown, D. J. (2001) Survey of future injectables. *Facial Plast Surg Clin North Am,* 9**,** 405-11.

Freshney, R. (1995) *Animal cell cultures,* Oxford, URL Press.

Gillies, R. J., Didier, N. & Denton, M. (1986) Determination of cell number in monolayer cultures. *Anal Biochem,* 159**,** 109-13.

Hayes, T. L. & Pease, R. F. (1968) The scanning electron microscope: principles and applications in biology and medicine. *Adv Biol Med Phys,* 12**,** 85-137.

Janeway, C. A., Travers, P., Walport, M. & Shlomchik, M. J. (2005) Immuno Biology, the immune system in health and disease. 6th ed. Oxon, Garland Science Publishing.

Klionsky, D. J., Cuervo, A. M. & Seglen, P. O. (2007) Methods for monitoring autophagy from yeast to human. *Autophagy,* 3**,** 181-206.

Kueng, W., Silber, E. & Eppenberger, U. (1989) Quantification of cells cultured on 96-well plates. *Anal Biochem,* 182**,** 16-9.

Laeschke, K. (2004) Biocompatibility of microparticles into soft tissue fillers. *Seminars in Cutaneous Medicine and Surgery,* 23**,** 214-217.

Lemperle, G., Morhenn, V. B., Pestonjamasp, V. & Gallo, R. L. (2004) Migration Studies and Histology of Injectable Microspheres of Different Sizes in Mice. *Plastic and Reconstructive Surgery,* 113**,** 1380-1390 10.1097/01.PRS.0000112764.22839.7A.

Lutolf, M. P. & Hubbell, J. A. (2005) Synthetic biomaterials as instructive extracellular microenvironments for morphogenesis in tissue engineering. *Nat Biotech,* 23**,** 47-55.

Naidoo, K., Rolfes, H., Easton, K., Moolman, S., Chetty, A., Richter, W. & Nilen, R. (2008) An emulsion preparation for novel micro-porous polymeric hemi-shells. *Materials Letters,* 62**,** 252-254.

Pretorius, E., Briedenhann, S., Marx, J. & Franz, R. C. (2006) Structural changes in the fibrin network of a pretoria family with dysfibrinogenemia: a scanning electron microscopical study. *Ultrastruct Pathol,* 30**,** 167-76.

Pretorius, E., Ekpo, O. E. & Smit, E. (2007) Comparative ultrastructural analyses of platelets and fibrin networks using the murine model of asthma. *Experimental and Toxicologic Pathology,* 59**,** 105-114.

Steynberg, T. (2010) Comparison of the *in vitro* effect of two-dimensional and three-dimensional polycaprolactone polymers on cell morphology, viability and cytotoxicity. *Physiology.* Pretoria, University of Pretoria.

Vine, A. K. (2009) Recent advances in haemostasis and thrombosis. *Retina,* 29**,** 1-7.

**Annexe A**

A. In vivo experiments

1. We will need ethics approval from the Animal Ethics Committee

2. Cells will have to be labelled with GFP in order to be able to track them in vivo

a. Carlo/Renier to assist

b. Will need to establish conditions needed for visualization of GFP in vivo

3. Cell types

a. Mouse embryo fibroblasts (MEF)

b. Mouse ear fibroblasts (MEaF)

c. Adipose-derived mesenchymal stem cells (MSCs)

4. Particles

Need to try out 2 doses to respond to Lara’s question about systemic inflammation being related to the number of particles

5. Time

a. 4-7 days to see acute inflammation

b. 8 weeks (does this correspond with the time in the experiment we have just completed?)

6. Morphology

a. Choice of fixative (including cryopreservation)

b. GFP visualization

c. CD45 IHC for leukocytes

7. Mice

a. Adult Balb/C

b. If done in triplicate, approximately 150 mice needed

8. Variables to be discussed (if we need to make the experiment smaller)

a. Cell types (may be able to use only 2)

b. Acute imflammation?

B. In vitro experiments

Need to repeat all the experiments that Andre/Annie did using MSCs

**Overview of in vivo experiments**

|  | **Fixative 1** | |  |  | **Fixative 2** | |
| --- | --- | --- | --- | --- | --- | --- |
| **Time** | **4-7 days** | **8 weeks** |  | **Time** | **4-7 days** | **8 weeks** |
| **Cell type** | **Particles** | **Particles** |  | **Cell type** | **Particles** | **Particles** |
|  |  |  |  |  |  |  |
| No cells | None | None |  | No cells | None | None |
|  | Dose1 | Dose1 |  |  | Dose1 | Dose1 |
|  | Dose2 | Dose2 |  |  | Dose2 | Dose2 |
|  |  |  |  |  |  |  |
| MEF | None | None |  | MEF | None | None |
|  | Dose1 | Dose1 |  |  | Dose1 | Dose1 |
|  | Dose2 | Dose2 |  |  | Dose2 | Dose2 |
|  |  |  |  |  |  |  |
| MEaF | None | None |  | MEaF | None | None |
|  | Dose1 | Dose1 |  |  | Dose1 | Dose1 |
|  | Dose2 | Dose2 |  |  | Dose2 | Dose2 |
|  |  |  |  |  |  |  |
| MSC | None | None |  | MSC | None | None |
|  | Dose1 | Dose1 |  |  | Dose1 | Dose1 |
|  | Dose2 | Dose2 |  |  | Dose2 | Dose2 |
|  |  |  |  |  |  |  |

**Note**

The following information will be needed to guide us in the planning of the next round of experiments:

1. Finalized description of work done to date in this project
2. Careful review of all work previously done with these particles by other groups in SA – UJ, Ampath (provided by Lara) and Annie and student
3. Thorough review of existing literature in this area (some PDFs already provided by Lara)

1. “Tissue harbour” refers to the advantages conferred by the unique morphology/design of the particle: the hollow particles each have a large port providing access for cells to their interiors, so that, subsequent to injection, the particles provide space for cells to proliferate and for nutrient exchange. The microporosity allows for efficient nutrient and oxygen/carbon dioxide exchange within the interior of the particles.
